# Homozygous MESP1 knock-in reporter hESCs facilitated cardiovascular cell differentiation and myocardial infarction repair

**DOI:** 10.7150/thno.42347

**Published:** 2020-05-23

**Authors:** Lin Wang, Fengzhi Zhang, Fuyu Duan, Rujin Huang, Xi Chen, Jia Ming, Jie Na

**Affiliations:** Center for Stem Cell Biology and Regenerative Medicine, School of Medicine, Tsinghua University, Beijing, 100084, China.

**Keywords:** Human embryonic stem cells, MESP1, Cardiomyocyte differentiation, Myocardial infarction, Transplantation

## Abstract

Different populations of cardiovascular progenitor cells have been shown to possess varying differentiation potentials. They have also been used to facilitate heart repair. However, sensitive reporter cell lines that mark the human cardiovascular progenitors are in short supply.

**Methods:** MESP1 marks the earliest population of cardiovascular progenitor cells during embryo development. Here, we generated a homozygous MESP1 knock-in reporter hESC line where mTomato gene joined to the MESP1 coding region via a 2A peptide, in which both MESP1 alleles were preserved. We performed transcriptome and functional analysis of human MESP1+ cardiovascular progenitor cells and tested their therapeutic potential using a rat model of myocardial infarction.

**Results:** MESP1-mTomato knock-in reporter faithfully recapitulated the endogenous level of MESP1. Transcriptome analysis revealed that MESP1+ cells highly expressed early cardiovascular genes and heart development genes. The activation of MESP1 relied on the strength of canonical Wnt signaling, peak MESP1-mTomato fluorescence correlated with the window of canonical Wnt inhibition during in vitro differentiation. We further showed that MESP1 bound to the promoter of the WNT5A gene and the up-regulation of WNT5A expression suppressed canonical Wnt/β-CATENIN signaling. Moreover, induced MESP1 expression could substitute the canonical Wnt inhibition step and promote robust cardiomyocyte formation. We used a configurable, chemically defined, tri-lineage differentiation system to obtain cardiomyocytes, endothelial cells, and smooth muscle cells from MESP1+ cells at high efficiency. Finally, we showed that the engraftment of MESP1+ cells repaired rat myocardial infarction model.

**Conclusions:** MESP1-mTomato reporter cells offered a useful platform to study cardiovascular differentiation from human pluripotent stem cells and explore their therapeutic potential in regenerative medicine.

## Introduction

Better understanding the mechanism of human cardiovascular progenitor cell fate decisions could provide important insights for heart and blood vessel regeneration. Mesoderm posterior 1 (*Mesp1*) is a helix-loop-helix homeobox transcription factor only transiently present during the early stages of heart development 1,2. It is initially expressed in the primitive streak, then in the lateral mesoderm that migrated anteriorly and formed the heart 1,2. *Mesp1* null embryos died around E10.5 due to severe defects in heart tube formation 1. Lineage tracing experiments demonstrated that *Mesp1* lineage cells contributed to multiple mesoderm lineages, including the heart, thymic mesenchymal cells, cranial skeletal muscles and hematopoietic stem cells (HSCs) 1,3-5.

Human pluripotent stem cells (hPSCs), including embryonic stem cells (ESCs) and induced pluripotent stem cells (iPSCs), can self-renew for long term in culture and differentiate to all types of cells in the body, thus provided an *in vitro* system to study the events during early human embryo development. We generated a homozygous MESP1 knock-in reporter hESC line where mTomato gene joined to the MESP1 coding region via a 2A peptide. Different from a previously reported MESP1^mCherry/w^/Nkx2-5^eGFP/W^ dual reporter hESC line, where one allele of MESP1 was replaced by the mCherry cassette 6,7, both MESP1 alleles were preserved in our MESP1-mTomato hESC line. The homologous knock-in MESP1-mTomato cells showed a sensitive response to the mesoderm induction signal and faithfully recapitulated the endogenous MESP1 expression. MESP1 can inhibit the canonical Wnt/β-CATENIN signaling by directly upregulating *WNT5A* expression. Using a chemically defined and monolayer differentiation system, and through the enrichment of MESP1^+^ cells, we can achieve highly efficient cardiomyocyte (CM), endothelial cell (EC) and smooth muscle cell (SMC) differentiation. Moreover, upon engraftment into the rat model of myocardial infarction (MI), MESP1^+^ cells differentiated to ECs and CMs, and significantly improved heart function. In summary, our work provided new insights about cardiovascular differentiation from hPSCs and offered a useful tool to explore the regeneration potential of hPSC derived cardiovascular progenitor cells.

## Methods

### hESC culture

H9 hESCs (WiCell Institute) were maintained on inactivated mouse embryonic fibroblast (MEF) cells in standard hESC medium at 37 ºC in a humidified atmosphere of 5% CO_2_ in the air 8. They were passaged with 1 mg/mL collagenase IV (Invitrogen) and seeded onto a 25 cm^2^ flask that had been previously coated with 0.1% gelatine solution (Sigma-Aldrich). For feeder-free culture, hESCs were grown for more than 3 passages in the absence of feeders in TeSR^TM^-E8^TM^ medium (STEMCELL Technologies).

### Generation of MESP1-mTomato knocking-in reporter cell line

A transcription activator-like effector nuclease (TALEN) pair was designed using online tool (http://boglabx.plp.iastate.edu/TALENT/). Tandem arrays of TALE repeats were synthesized by ViewSolid Biotech (http://www.v-solid.com) and joined to heterodimeric Fok I endonuclease. The homologous recombination donor vector consists of the following elements: the *MESP1* left arm, T2A fused with a membrane-bound tdTomato (mTomato), PGK promoter driving puromycin resistance gene (PGK-Puro), *MESP1* right arm and MC-1 promoter driving TK gene. H9 cells were electroporated with TALEN and donor vectors using Neon microporator (Invitrogen). After puromycin selection, individual undifferentiated colonies were picked and expanded for characterization. Detailed verification methods were described in Supplemental Methods.

### RNA isolation, Quantitative PCR (Q-PCR) and RNA sequencing

Undifferentiated hESCs, differentiation day 3 and day 5 cells were collected. mTomato^+^ and mTomato^-^ cells were sorted by Aria III flow cytometer (Becton Dickinson). Total RNA was extracted using the RNeasy Plus Mini Kit (Qiagen) and treated with RNase free DNase. 1 μg RNA of each sample was used for reverse transcription with Superscript III (Invitrogen). Q-PCR reactions were done using GoTaq qPCR Master Mix (Promega) in a CFX96 Real-Time System (Bio-Rad). The sample input was normalized against the Ct (Critical Threshold) value of the house-keeping gene GAPDH. Relative quantification of each gene was performed using the Bio-Rad CFX Manager program. Primer sequences are listed in Table S1.

For high-throughput sequencing, the cDNA library was prepared using TruSeq™ RNA Sample Preparation kit (Illumina), and high-throughput sequencing was done at the Biopic sequencing facility of Peking University (http://biopic.pku.edu.cn/english/). Clean reads were mapped to the human genome (hg19) using Tophat2 (Version 2.0.8) software with the Refseq annotation. Gene expression values were represented as FPKM (Fragments Per Kilobase Million) calculated by Cufflinks (version 2.1.1). Differentially expressed genes (DEGs) were analyzed by Cuffdiff (version 2.1.1) with the parameters “-p 4, -c 10”. Genes satisfied the threshold “mini alignment count ≥10, |Fold change| ≥2, *p* < 0.05, FDR < 0.05” were considered significant DEGs. DEGs were then subjected to principal component analysis (PCA), hierarchical cluster, and gene set enrichment analysis. PCA was performed using the “prcomp” function of R and drawn by “gplots” package of R. The heatmaps were made using the heatmap2 function of 'gplots' R package with a hierarchical clustering method. Gene set enrichment analysis (GSEA) (http://www.broadinstitute.org/gsea/index.jsp) was performed using the 'Signal2Noise' ranking metric. The enrichment of Gene ontology (GO) and Kyoto Encyclopedia of Genes and Genomes (KEGG) pathways was analyzed using DAVID (http://david.abcc.ncifcrf.gov/).

### Cardiovascular differentiation, growth factors and small molecules

For monolayer based cardiomyocyte differentiation, we employed a protocol published by Lian et al. 9 with slight modifications. In brief, H9 hESCs maintained on Matrigel (Growth factor reduced, BD Biosciences) in E8 medium were dissociated into single cells with Accutase (Invitrogen), then seeded onto Matrigel-coated tissue culture dishes at a density of 5 × 10^4^ cells/cm^2^ and cultured in E8 medium for 4 days. For the signaling pathway test, after 4 days of culturing in E8 medium, they were switched to RPMI-1640 medium supplemented with 1 × B-27 minus insulin (Invitrogen). Growth factors BMP4 (R&D) 25 ng/mL; bFGF (Peprotech) 5 ng/mL and chemical inhibitors PD0325901 (Tocris) 2 μmol/L; LY294002 (Tocris) 5 μmol/L; Retinoic Acid (RA) (Sigma) 2 μmol/L were added at desired concentration and time window. For CM differentiation, cells were first cultured in RPMI-1640 medium supplemented with 1 × B27 minus insulin (Invitrogen), CHIR or CHIR + BMP4 was added during the first 24 h of differentiation then removed. 48 h later, 5 μM IWP2 was added for 48 h. After IWP2 removal, cells were cultured in RPMI-1640/B-27 minus insulin medium for another 2 days before switching to RPMI-1640 / B-27 medium until the appearance of beating areas. For EC differentiation, day 3 MESP1^+^ cells were dissociated with Accutase for 10 min and plated onto Matrigel-coated culture dishes at a density of 2 × 10^4^ cells/cm^2^ in RPMI-1640/B-27 medium supplemented with 25 ng/mL VEGF and 10 ng/mL FGF2 (both Sino Biological) for 5 days. For SMC differentiation, PDGF-BB 10 ng/mL (Sino Biological) was added. For the vascular tube formation assay, 1 × 10^5^ ECs were plated into one well of 24-well plates coated with Matrigel (BD) and incubated at 37 °C for 12 h. For the DiI-ac-LDL uptake assay, ECs were incubated with 20 mg/mL of DiI-Ac-LDL (Yeasen) at 37 °C for 6 h, washed with phosphate buffer saline (PBS), and stained with Hoechst 33258 (Dojindo).

### Antibodies, immunostaining, western blot, and Fluorescent-activated cell sorting (FACS) analysis

For immunostaining, cells were fixed with 4% paraformaldehyde, permeabilized in 0.5% Triton X-100 (Sigma), blocked in 10% normal goat serum (Origene) and then incubated with primary antibodies against cTnT (Thermo), CD31 (Santa Cruz), hCD31(human specific CD31, ab76533, Abcam), αSMA (ZSGB-Bio http://www.zsbio.com/), hNA (Anti-human Nuclei Antibody, clone 235-1, MAB1281, Merck_millipore) in 4 °C overnight and detected by DyLight 488- or 549-conjugated secondary antibodies (Thermo). Nuclei were stained with DAPI (Sigma). A Nikon Ti-U fluorescence microscope was used for image acquisition. For western blot, cells were lysed in RIPA buffer (Applygen*,* applygen.com.cn) with Protease Inhibitor Cocktail (Roche). Total proteins were separated by 12% SDS/PAGE, transferred to nitrocellulose membrane (Whatman). The membrane was blocked with 5% non-fat dry milk in TBST and then incubated with primary antibodies against MESP1 (APR39374_P050, AVIVA Systems Biology), Flag-tag (M2, Sigma), β-CATENIN (Cell signaling technology), phosphorylated-β-CATENIN (Bioworld) and ACTIN (Proteintech). After washing, the membrane was incubated with anti-mouse or rabbit peroxidase-conjugated secondary antibodies (ZSGB-Bio http://www.zsbio.com/). Specific bands recognized by antibodies were revealed by ECL reagent (Pierce). Quantification of the western blot was performed by Image J. For FACS analysis, cells were first dissociated with 0.05% Trypsin in 0.2% EDTA and PBS, then washed and stained with CD56 (eBioscience), CD13 (Biolegend), E-Cadherin (Cell Signaling Technology), PDGFRα (R&D), VE-Cadherin/CD144 (Cell Signaling Technology) antibodies followed by DyLight 488- or 549-conjugated secondary antibodies (Thermo). FACS was performed on a BD FACSCalibur™ flow cytometer (Becton Dickinson).

### Doxycycline (Dox) inducible MESP1 and WNT5A expression hESC line construction

The Dox inducible MESP1 and WNT5A H9 hESC line were generated using PiggyBac transposon system. Plasmids containing the PiggyBac (PB) transposon terminal repeats and HyperPB transposase were general gifts from Sanger Institute, Cambridge, UK. A plasmid with PB 5ʹ and 3ʹ terminal repeat flanking a CAG promoter driving rtTA gene followed by an internal ribosomal entry site (IRES) and puromycin resistant gene was engineered. The PB-TRE-MESP1-Flag plasmid was constructed from pPB-Ubi.eGFP-neo (Sanger) by replacing Ubiquitin promoter driving GFP cassette with TRE3G promoter (ClonTech) driving cDNA encoding MESP1-3×Flag tag fusion protein. The two plasmids were transfected into H9 hESCs together with HyperPB mRNA using Neon (Invitrogen). Dox inducible MESP1 clones were selected on DR4 MEF feeders in the hESC medium. Several single colonies were picked, expanded, and tested for induced expression of MESP1-Flag by Dox. For Dox inducible WNT5A, PB-TRE-WNT5A-neo plasmid was constructed by replacing the MESP1-Flag fragment in pPB-TRE-MESP1-Flag plasmid with the coding region of human WNT5A.

### Chromatin immunoprecipitation (ChIP)-Q-PCR

For MESP1 ChIP-Q-PCR, 5 × 10^6^ cells were fixed in 1% formaldehyde for 10 min at room temperature and quenched in 0.125 M glycine. Then cells were scraped, resuspended in lysis buffer (50 mM Tri-Cl, pH = 8.1, 10 mM EDTA, 0.5% SDS, and protease inhibitors) and incubated on ice for 10 min. Then cells were sonicated to generate fragments about 200 bp in length. Samples were pre-cleared for 30 min with Protein A/G UltraLink Resin (Thermo), followed by immunoprecipitation with M2 antibody (Sigma) against Flag tag overnight at 4 °C. After incubation, the immunocomplexes were incubated with Protein A/G UltraLink Resin at 4 °C for 4 h and subsequently washed with low-salt wash buffer (20 mM Tris HCl pH = 8, 150 mM NaCl, 1 mM EDTA, 0.1% SDS, 1% TritonX-100, 0.1% Na-deoxycholate and protease inhibitors), high-salt wash buffer (20 mM Tris HCl pH = 8, 500 mM NaCl, 0.1% SDS, 1 mM EDTA, 1% Triton X-100, 0.1% Na-deoxycholate and protease inhibitors), LiCl wash buffer (10 mM Tri-Cl, pH 8.1, 250 mM LiCl, 1 mM EDTA, 0.5% Deoxycholic acid, 0.05% IGEPAL-CA630) and TE (10 mM Tri-Cl, pH = 8.1, 1 mM EDTA) buffer. Immunocomplexes were eluted in elution buffer (1% SDS, 100 mM NaHCO3, and protease inhibitors), and cross-linking was reversed at 65°C overnight. Samples were treated with proteinase K, extracted with phenol/chloroform, and precipitated with ethanol. The negative control for MESP1 ChIP was Flag-tag antibody ChIP from non-induced cells. Q-PCR primers used to detect related regions were list in Table S2.

### The rat model of MI, cell transplantation, electrocardiography, and histology analysis

All procedures in this section were approved by the Internal Review Board, Laboratory Animal Research Center, Tsinghua University. Acute MI in Sprague-Dawley rats (250 ± 10 g) was induced by ligation of the left coronary artery (6-0 Prolene suture) in sodium pentobarbital-anesthetized (30 mg/kg) male as described 10-12. Ten minutes later, the infarct region was confirmed by myocardial blanching. Injections made along the border zone of the infarcted area at 3 sites (below the left atrium, in the middle portion of the left ventricle, and at the apex) with a total volume of 100 μL using a 28-gauge needle. Rats were divided into 3 groups: (1) Sham group (*n* = 6) underwent thoracotomy and cardiac exposure with no coronary ligation; (2) Control group (*n* = 10) injected with 100 μL PBS, (3) MESP1 group (*n* = 7), 1 × 10^7^ MESP1^+^ cells in PBS (100 μL) were injected. All rats were immunosuppressed by daily administration of cyclosporine A (10 mg/kg/day) and tacrolimus (8 mg/kg/day) from 3 days before transplantation until euthanasia. Echocardiography was carried out 28 days after transplantation using a Vevo 2100 system (Visualsonic, Toronto, Canada). Using M-mode tracings, the following parameters were measured: left ventricle end-diastolic dimension (LVEDD), left ventricle end-systolic dimension (LVESD), and left ventricle ejection fraction (LVEF). Also, the left ventricle fractional shortening (LVFS) was calculated according to the following equation: LVFS = [(LVEDD - LVESD) /LVEDD] × 100. The left ventricular remodelling index (LVRI) was obtained by calculating the ratio of left ventricular mass (LVM) to left ventricular end-diastolic volume (LVEDV) as described in 13. 28 days after cell transplantation, rats were anesthetized, and their hearts were first perfused with 50 mL PBS then washed with 0.1 mol/L KCl using a 25G butterfly needle inserted into the left ventricle via the apex. Hearts were removed and fixed at 4 ºC in 4% PFA overnight. After embedding in paraffin, the samples were sectioned and processed for Masson's trichrome staining, β-CATENIN (Beyotime) staining and human CD31 staining according to standard procedures. Images were captured with a slide scanner microscope Axio Scan. Z1 (Carl Zeiss, Gottingen, Germany) and ZEN software (Carl Zeiss). The myocardial infarct size and immunostainings were measured using Image J software. The vessel density was analyzed in the myocardial infarction area using AngioTool 14.

### Statistical analysis

Data are presented as mean ± standard error of the mean (SEM). Statistical significance was determined by Student's *t*-test (two-tail) for two groups or one-way ANOVA for multiple groups using OriginPro 8 software, *p* < 0.05 was considered significant.

## Results

### Generation of MESP1-mTomato reporter hESC by TALEN facilitated homologous recombination

**Figure 1:**
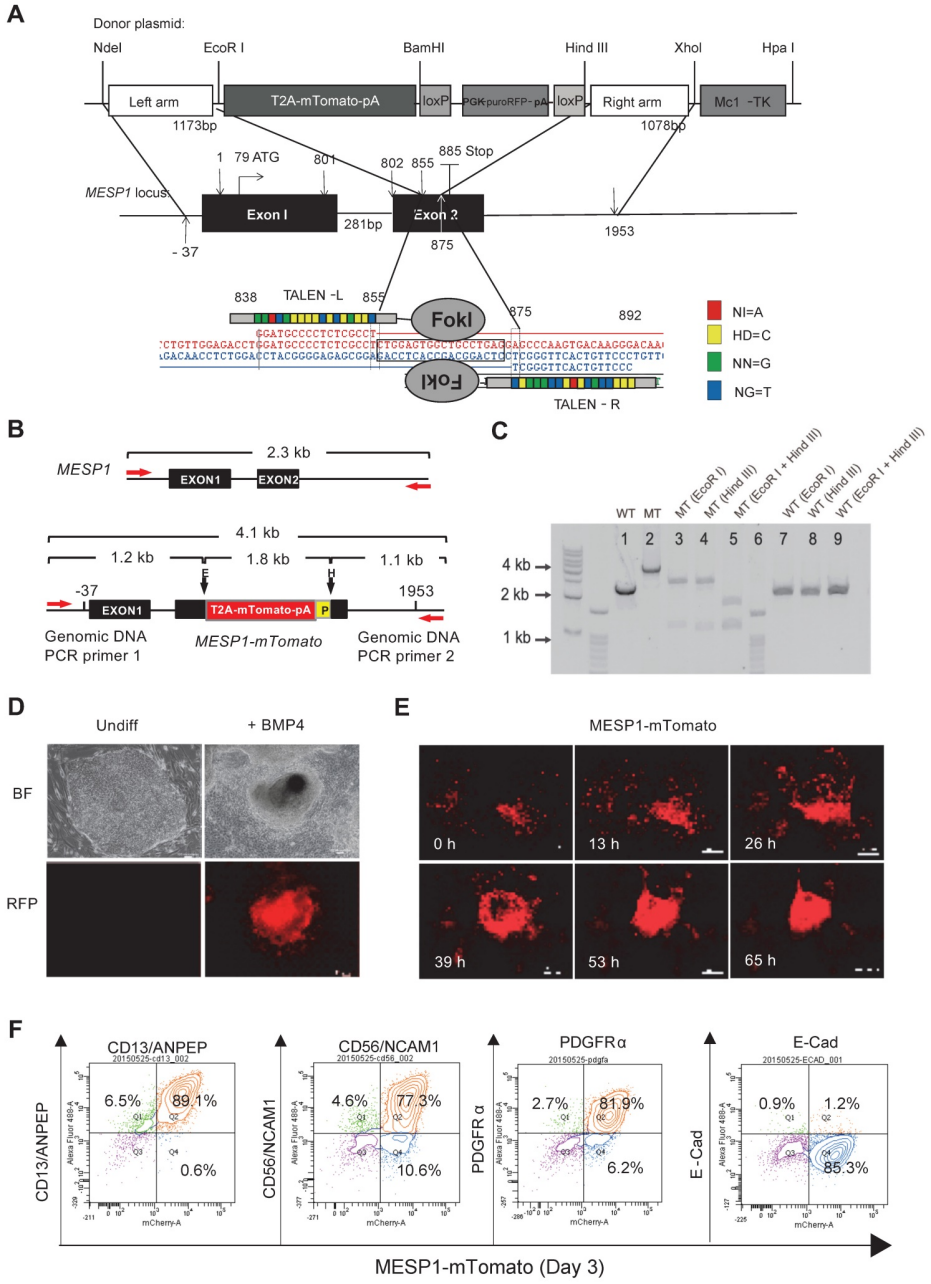
** Generation of *MESP1* knock-in H9 hESC.** (**A**) Schematic view of *MESP1* targeting strategy. Donor plasmid: T2A-mTomato-pA is a T2A self-cleaving peptide sequence joint with mTomato and a polyadenylation sequence (pA); PGK, phosphoglycerate kinase promoter; puroRFP, puromycin resistance gene fused with red fluorescence protein (RFP) gene; loxP, loxP sites for Cre recombinase-mediated PGK-puroRFP removal; MC1-TK, MC1 promoter driving TK gene for negative selection. White arrow indicates the cutting site of the *MESP1* TALEN pair. Below, *MESP1* TALENs and their recognition sequence. The DNA binding specificity of the repeat variable di-residue (RVD) is: NI = A (red), HD = C (yellow), NN = G (green), NG = T (blue). (**B**) Schematic view of MESP1-mTomato knock-in locus and positions of genomic DNA PCR primers (red arrows). The wild type (WT) fragment is 2.3 kb, and the knock-in locus (MT) fragment is 4.1 kb. EcoR I (E) digestion of the MT fragment generates two fragments of 2.9 kb and 1.2 kb. Hind III (E) digestion generates two fragments of 1.1 kb and a 3 kb. P (loxP). (**C**) Genomic DNA PCR confirming correct targeting. Lane 1: WT fragment (2.3 kb), lane 2: MT fragment (4.1 kb), lane 3-5: EcoR I or Hind III or together cut MT fragment to produce bands of the expected size, lane 6: marker, lane 7-9: WT fragment cannot be cut by EcoR I or Hind III. (**D**) Undifferentiated MESP1-mTomato cells (Undiff) did not have red fluorescence, BMP4 induced strong mTomato expression. Bright-field images (BF), fluorescence images (RFP). Scale bars, 100 µm. (**E**) Selected frames from a time-lapse movie of MESP1-mTomato^+^ cell emergence. Time points are as indicated, scale bars: 250 µm. See also Movie S1. (**F**) Flow cytometry analysis showing MESP1-mTomato^+^ cells highly expressed mesoderm surface markers CD13/ANPEP, CD56/NCAM1 and PDGFRα, but did not express epithelial cell marker E-Cadherin (E-Cad). *n* > 3.

We utilized transcription activator-like effector nuclease (TALEN) mediated homologous recombination to knock in the mTomato gene after the last amino acid codon of the *MESP1* gene, connected by a self-cleaving 2A peptide sequence and just before the 3ʹ untranslated region (UTR) of *MESP1* (Figure 1A). Upon MESP1-2A-mTomato expression, the 2A-mTomato will be cleaved off, leaving the endogenous MESP1 protein intact. Moreover, both MESP1 alleles, including UTRs, were preserved, and the expression of the endogenous protein will not be affected.

Genomic DNA PCR confirmed the correct targeting and the removal of the PGK-puro-RFP cassette (Figure 1B-C, and Supplementary Figure S1A-D). We chose one clone where mTomato was correctly targeted into both alleles of *MESP1* for experiments in this study. MESP1-mTomato reporter cells had a normal 46 XX diploid karyotype (Figure S1E), and they were able to self-renew and differentiate into neuroectoderm and endoderm derivatives *in vitro* (Figure S1F) and formed fully differentiated teratoma in nude mice (Figure S1G).

Next, we tested the responsiveness of MESP1-mTomato reporter. No red fluorescence can be detected in undifferentiated cells (Figure 1D). Upon adding BMP4, red fluorescence started to appear after 24 h and reached a peak at day 3 (Figure 1E). The mTomato fluorescence lasted for another two days and decreased quickly afterward. No red fluorescence can be seen when cells were induced with Activin A to form endoderm or treated with BMP inhibitor LDN193189 to form neuroectoderm (data not shown). Time-lapse imaging revealed that mTomato^+^ cells first appeared in a scattered manner, then migrated actively, displayed stronger red fluorescence, and converged together to form bigger clumps (Figure 1E and Movie S1). This behavior resembles lateral mesoderm cells migrating anteriorly to form the heart crescent.

Finally, we analyzed mesoderm surface marker expression by flow cytometry. Differentiation day 3 MESP1^+^ cells highly expressed mesoderm specific surface protein CD13 (89.1%), CD56 (77.3%), and PDGFRα (81.9%) but not E-Cadherin (1.2%) which is an epithelial marker on hESC surface (Figure 1F). We also verified that once differentiated, MESP1-mTomato^+^ cells do not have tumorigenicity. Five million sorted mTomato^+^ cells were injected subcutaneously into nude mice. After 4 weeks, no teratoma was observed in any of the mice injected (*n* = 4) (Figure S1H).

Sum together, MESP1-mTomato reporter cells represented a useful system to monitor the early events of human cardiovascular development *in vitro*.

### MESP1-mTomato reporter is a sensitive indicator of cardiovascular mesoderm differentiation

**Figure 2:**
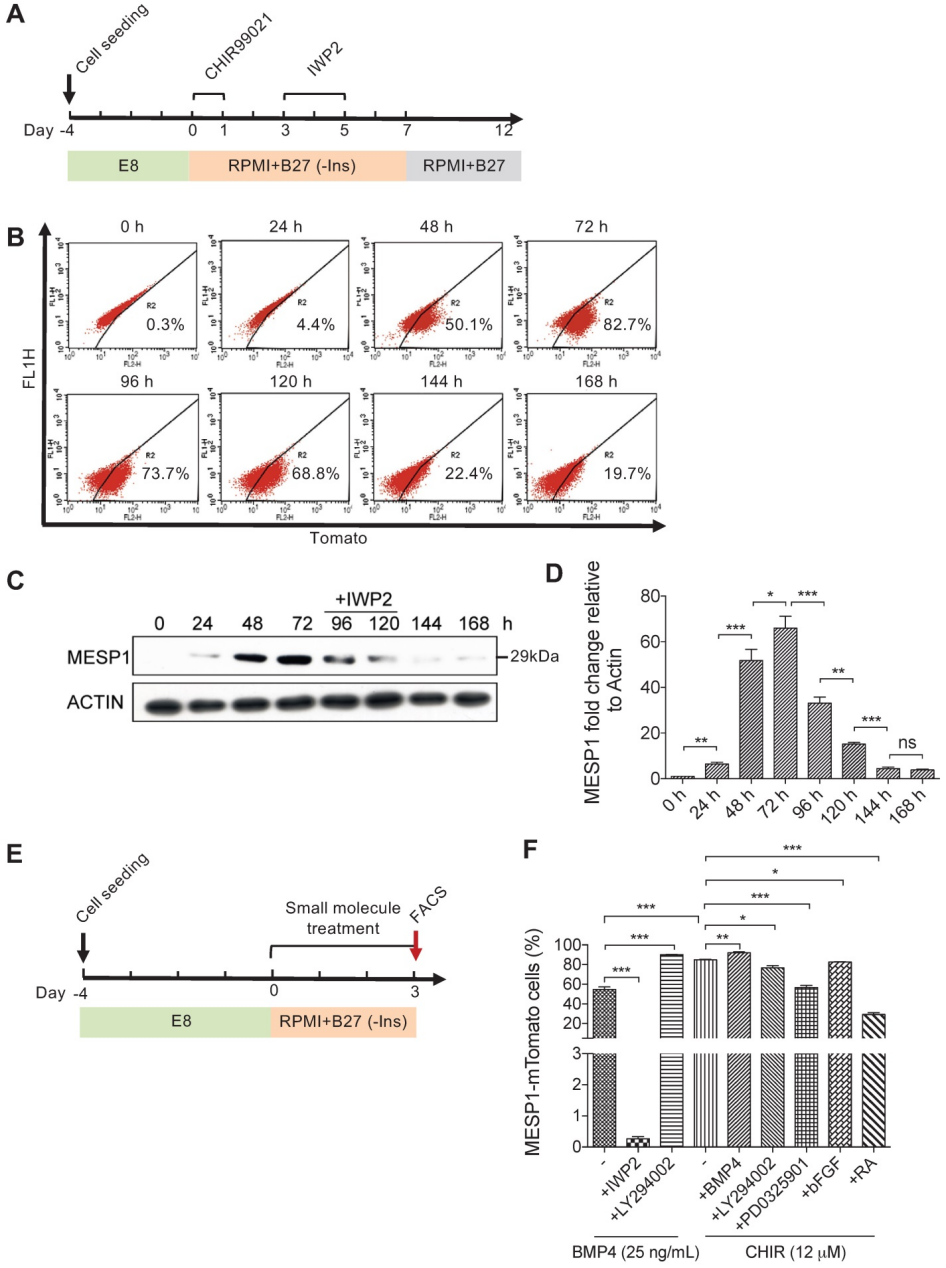
** Canonical Wnt signaling controls the induction of MESP1-mTomato^+^ cells.** (**A**) Cartoon depicting the CM differentiation protocol. (**B**) Flow cytometry analysis of mTomato fluorescence in cells during 168 h (7 days) of CM differentiation (*n* = 3). (**C**) Western blot showing endogenous MESP1 protein levels during CM differentiation (*n* = 3). (**D**) Quantification of MESP1 protein level based on the western blot. (**E**) Experiment timeline for signaling pathway test: 4 days after replating and culturing in E8 medium, MESP1-mTomato reporter cells were treated with small molecules activating or inhibiting various signaling pathways for 72 h, then harvested for flow cytometry analysis. (**F**) Bar graph quantification of MESP1-mTomato^+^ cell percentage after growth factor or small molecule treatment (as listed at the bottom) (*n* = 3). “-” no small molecule added. Data is shown as mean ± SEM, *p < 0.05, **p < 0.005, ***p < 0.001, Student's *t*-test.

To study the signals that affect the formation of cardiovascular progenitor cells, we first monitored the dynamic change of MESP1-mTomato fluorescence during cardiovascular differentiation. In this series of experiments, we used the differentiation system described by Lian et al. 15,16, which only employed small molecules to modulate canonical Wnt signaling. Undifferentiated MESP1-mTomato cells were dissociated as single cells and seeded on Matrigel. After 4 days of culture in E8 medium, when they grew to appropriate confluency, the medium was switched to RPMI 1640 supplemented with B27 minus insulin (hereafter referred to as RPMI/B27-I). GSK3 inhibitor CHIR99021 was added during the first day to induce mesoderm differentiation, then the Wnt inhibitor IWP2 was added during day 3-5. After 7 days, the medium was replaced with RPMI/B27 (Figure 2A). Flow cytometry analysis was carried out to quantify the percentage of mTomato^+^ cells every 24 h. The percentage of mTomato^+^ cells increased dramatically at 48 h, and by 72 h, 82.7% of cells were mTomato^+^ (Figure 2B). Interestingly, during the IWP2 treatment on day 4 and 5, mTomato^+^ cells maintained at relatively high levels (73.7% and 68.8%, respectively) (Figure 2B). On day 6 and 7 after IWP2 withdraw, the percentage of mTomato^+^ cells decreased to 22.4% and 19.7% (Figure 2B). We also performed western blot followed by quantification of endogenous MESP1 protein in the reporter cells (Figure 2C-D). A 29 kDa band corresponding to the size of MESP1 protein became evident after 48 h of differentiation (Figure 2C). MESP1 protein level peaked at 72 h and decreased gradually during 72-120 h, then quickly disappeared (Figure 2C). Above results confirmed that the MESP1 homozygous knock-in reporter faithfully reflected the expression of endogenous *MESP1* gene. We next tested how different signaling pathways may affect MESP1^+^ cell percentage. The same differentiation system was used, and cells were analyzed by flow cytometry on day 3 (Figure 2E). BMP4 is a classic mesoderm inducing growth factor and 25 ng/mL BMP4 alone can induce nearly 60% of cells to express mTomato in RPMI/B27-I medium (Figure 2F). Wnt inhibitor IWP2 completely abolished the induction of mTomato fluorescence by BMP4, suggesting that BMP4 functions through inducing endogenous Wnt ligands (Figure 2F). PI3K inhibitor LY294002 further increased the percentage of mTomato^+^ cells to nearly 100% after BMP4 treatment (Figure 2F). PI3K can activate AKT, which favors neural differentiation 17. This result is consistent with the mechanism of removing insulin. A high concentration of CHIR99021 (12 μM) alone induced more than 80% mTomato^+^ cells. Under this condition, blocking AKT or ERK with LY294002 or PD0325901, slightly but significantly reduced the percentage of mTomato^+^ cells (Figure 2F). The addition of Retinoic Acid (RA) decreased the percentage of mTomato^+^ cells to about 30% (Figure 2F). RA signaling had been shown to restrict the cardiac progenitor pool in zebrafish 18. It is possible that RA signaling antagonizes Wnt signaling or activates master genes of other lineages to reduce the number of MESP1 progenitor cells.

### Transcriptome profiling of MESP1^+^ cells during cardiac differentiation

**Figure 3:**
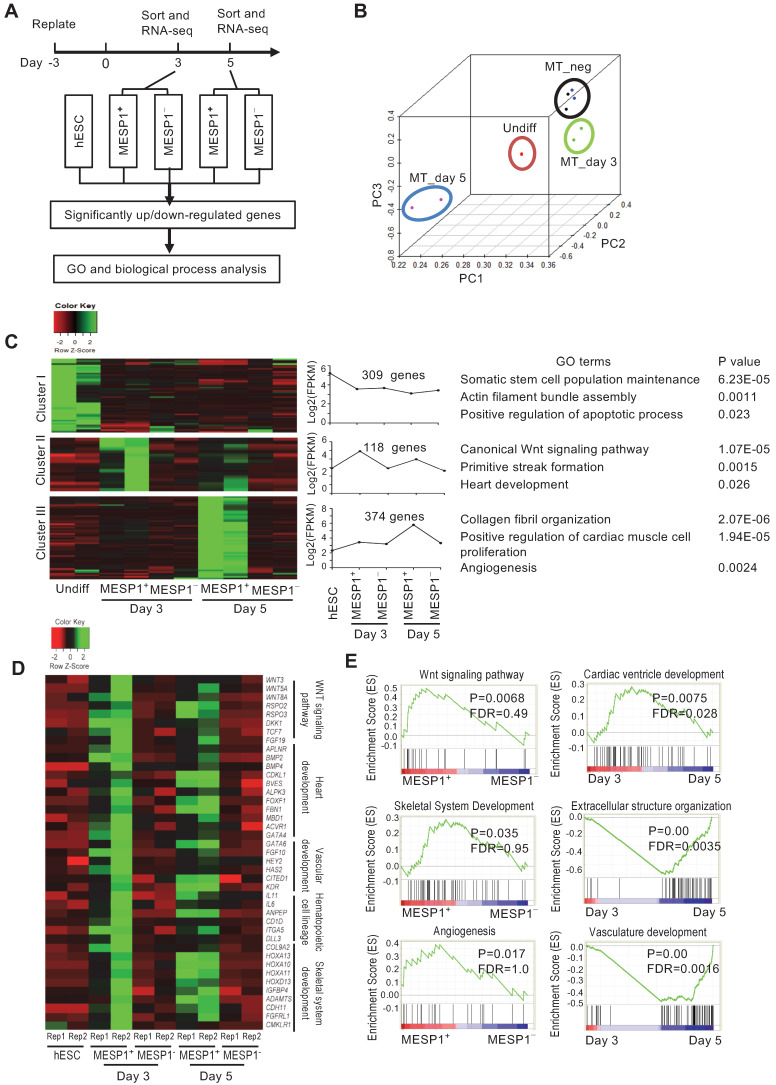
** Transcriptome analysis of MESP1-mTomato^+^ cardiovascular progenitor cells.** (**A**) Flow chart of MESP1-mTomato cell gene expression analysis. (**B**) PCA graph showing that hESCs, MESP1-mTomato^+^ cells at day 3 and 5 (MT_day3, MT_day5), and MESP1-mTomato^-^ cells (MT_neg) were separated from each other. (**C**) Representative gene expression patterns in day 3 and day 5 MESP1^+^ cells. The number of genes in each category, representative GO terms and enrichment *P* values were listed on the right side. (**D**) Heatmaps showing that many key regulators of cardiovascular development were differentially expressed in day 3 or day 5 MESP1^+^ cells, including Wnt pathway members, heart development genes, endothelial genes, hematopoietic cell genes and skeletal development genes. (**E**) GSEA plot of selective biological process enriched in MESP1^+^ or MESPI^-^ cells, day 3 or day 5 MESP1 cells.

To characterize the transcriptome of MESP1^+^ cells during cardiovascular differentiation, we performed high-throughput RNA sequencing of sorted differentiation day 3 and day 5 mTomato^+^ and mTomato^-^ cells. Principal component analysis (PCA) showed that day 3 and day 5 MESP1^+^ cells were distinct populations from hESCs and MESP1^-^ cells (Figure 3B). In the heatmap, day 3 and 5 MESP1^+^ cells were clustered together and separated from undifferentiated hESCs and MESP1^-^ cells (Figure S2A). Based on the dynamic change of gene expression, 309 genes were down-regulated more than 2 folds (fold change ≥ 2, *p* < 0.05, *q* < 0.05) after mesoderm differentiation (Figure 3C top row, Cluster I, Table S3). GO analysis showed that these genes are involved in stem cell maintenance, actin filament assembly, and apoptosis. 118 genes were specifically enriched in MESP1^+^ cells (fold change ≥ 2,* p* < 0.05, *q* < 0.05) compared to hESCs and MESP1^-^ cells) on both day 3 and day 5, many of them associated with canonical Wnt signaling (*p* = 1.07 × 10^-5^) (Figure 3C middle row, Cluster II, Table S3). Genes that regulate primitive streak formation and heart development also highly represented in this group. 374 genes were significantly higher expressed in day 5 MESP1^+^ cells (upregulated more than 2 folds compared to hESCs, MESP1^-^ cells, and day 3 MESP1^+^ cells) (Figure 3C bottom row, Cluster III, Table S3). The dominant GO classes of these group genes are collagen fibril organization, cardiac muscle cell proliferation, and angiogenesis, which are indicative of cardiovascular lineage commitment.

Heatmap representation of crucial regulators of cardiovascular differentiation, such as Wnt pathway members: *WNT3*, *WNT5A*, *WNT8A*, *RSPO2*, and *RSPO*3; heart development genes: *APLNR*, *BMP2*, *BMP4*, *FOXF1*, *GATA4*; endothelial genes: *GATA6*, *HEY2*, *KDR*; hematopoietic cell genes: *IL11*, *IL6*, *DLL3*; and skeletal development genes: *COL9A2*, *HOXA10*, *11* and *13*, *ADAMTS4* were highly expressed in day 3 or day 5 MESP1^+^ cells (Figure 3D). According to gene set enrichment analysis (GSEA), the transcriptome of MESP1^+^ cells was enriched in Wnt signaling pathway, angiogenesis, skeletal system development genes and negative regulation of cell death genes (Figure 3E and Figure S3). Day 5 MESP1^+^ cells increased the expression of extracellular structure organization, vasculature development, and cardiac ventricle development genes compared to day 3 (Figure 3E). Heatmap of selected genes showed that *MESP1* was highly expressed in the mTomato^+^ cells and almost undetectable in mTomato^-^ cells. *T/Brachyury*, *GATA4*, *KDR*, and *PDGFRα* also showed a similar trend (Figure S2B).

### MESP1 downregulated canonical Wnt signaling by activating *WNT5A* expression

**Figure 4:**
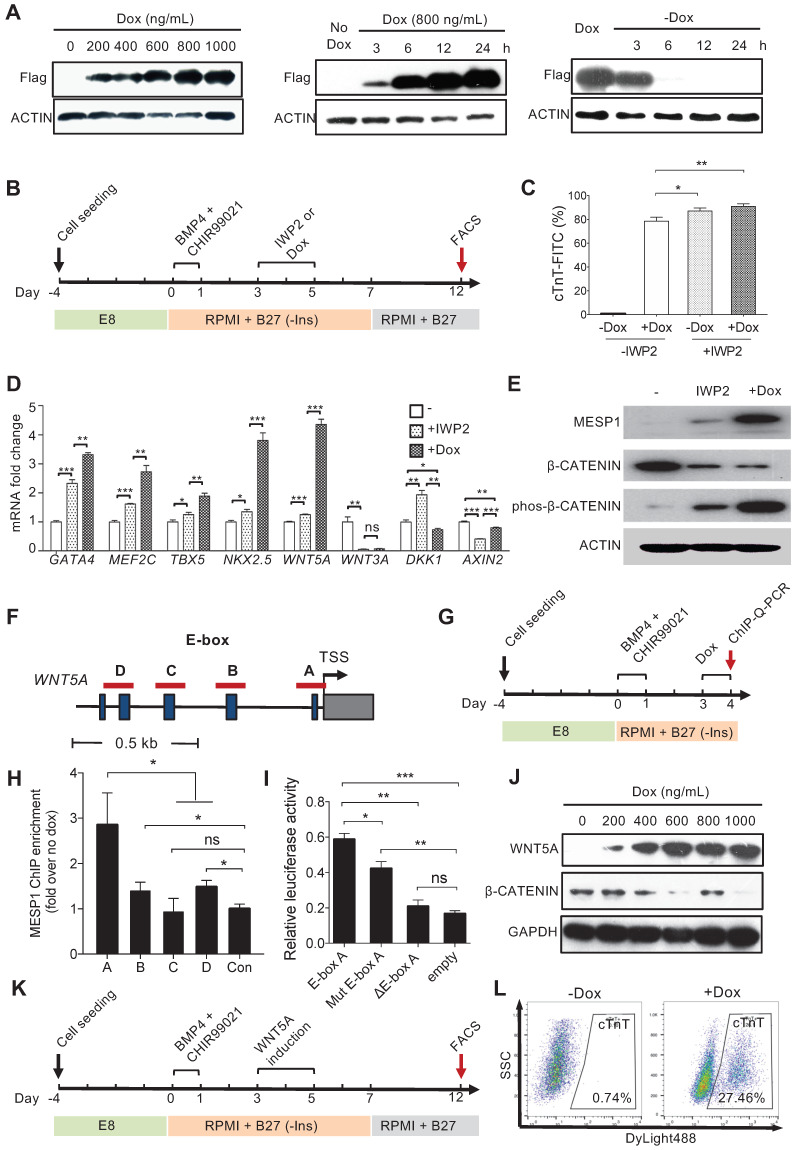
** MESP1 represses canonical Wnt/β-CATENIN signaling during hESC cardiac differentiation.** (**A**) Dox inducible MESP1 expression system. Left, dosage effect of Dox on MESP1-Flag protein expression (Dox concentration is indicated at the top). Middle and right, MESP1-Flag protein was induced 3 h after Dox addition and disappeared 6 h after Dox withdraw. ACTIN was used as the loading control in all experiments. (**B**) Schematic view of the differentiation protocol. Cells were treated with 25 ng/mL BMP4 and 2 µM CHIR99021 for 24 h. 48 h after BMP4 and CHIR99021 removal, 5 µM IWP2 or 400 ng/mL Dox was added for 48 h. After IWP2 or Dox treatment, cells were cultured in RPMI/B27 minus insulin medium until day 7, then changed to RPMI/B27 medium until day 12. (**C**) Bar graph showing cTnT^+^ cell percentage in untreated, Dox, or/and IWP2 treated cells based on flow cytometry analysis (*n* = 3). Data represent mean ± SEM. (**D**) Q-PCR analysis of genes induced by MESP1. Dox or IWP2 were added during day 3-5. The expression levels of cardiac and Wnt pathway genes were normalized against GAPDH. The expression levels in untreated cells were set as “1”. (*n* = 3). (**E**) Western blot showing MESP1, phosphorylated β-CATENIN (phos-β-CATENIN), and total β-CATENIN protein levels in cells treated IWP2 or Dox, ACTIN was used as the loading control (*n* = 3). (**F**) Schematic view of *WNT5A* promoter region. Dark blue boxes indicated E-box motifs. Red lines indicate regions tested in ChIP-Q-PCR assay. TSS: transcription start site. (**G**) Timeline of ChIP-Q-PCR analysis. (**H**) ChIP-Q-PCR analysis of MESP1 binding to E-box motifs on the *WNT5A* promoter. A non-related genomic region was used as negative control. Results were normalized against the enrichment of the same DNA fragment without Dox induction. A, B, C and D are E-box regions depicted in (F). (**I**) Luciferase reporter assay showing the transcription activity of E-box A (*WNT5A* promoter); mut E-box A (*WNT5A* promoter with E-box A mutated); ΔE-box A (E-box A deleted *WNT5A* promoter). Empty vector was used as negative control. Data represent the mean ± SEM, *n* = 3, *p < 0.05, **p < 0.005, ***p < 0.001, Student's *t*-test. (**J**) Western blot showing induction of WNT5A in hESCs reduced the level of total β-CATENIN protein. GAPDH, loading control. (**K**) Schematic view of differentiation protocol, cells were treated with WNT5A instead of IWP2 during day 3-5. (**L**) FACS analysis of cTnT on day 12 of differentiation.

We next wish to test the functional significance of the temporal expression pattern of *MESP1*. To this end, we engineered a Doxycycline (Dox) inducible Flag-tagged MESP1 hESC line. Western blot showed that MESP1 protein was induced 3 h after 800 ng/mL Dox addition, then rise to substantial-high level at 6 h and reached the highest level at 24 h (Figure 4A and Figure S4A). When we remove Dox from the medium, ectopic MESP1 diminished in 6 h (Figure 4A and Figure S4A). The above results indicated that our inducible system was sensitive and could respond quickly to Dox. Next, we added Dox in RPMI/B27-I medium during day 3-5 with or without IWP2 and analyzed the percentage of cTnT^+^ cells on day 12 (Figure 4B). Without IWP2 or Dox, very few cells expressed cTnT on day 12 (Figure 4C). Surprisingly, MESP1 induction by Dox during day 3-5 without IWP2 treatment was highly efficient to generate cTnT^+^ cells (78.54 ± 1.93%, *n* = 3) (Figure 4C), and many beating areas could be observed in the dish (Movie S2). When cells were treated with Dox combined with IWP2, the efficiency of CM formation increased up to 91.01 ± 1.22% (Figure 4C). Moreover, cells overexpressing MESP1 expressed a higher level of cardiac core transcription factors such as *GATA4*, *MEF2C*, *TBX5*, and *NKX2.5* (Figure 4D). Interestingly, among Wnt pathway members, a non-canonical Wnt ligand, *WNT5A,* was robustly induced by MESP1, while the level of *WNT3A*, *DKK1*, and *AXIN2* decreased (Figure 4D). The activation of the non-canonical Wnt pathway could suppress canonical Wnt/β-CATENIN signaling 19. Western blot analysis showed that inducing MESP1 for 48 h (day 3-5) indeed markedly increased phosphorylated β-CATENIN and downregulated total β-CATENIN protein at day 5 (Figure 4E and Figure S4B). Together, these results strongly suggested that MESP1 may have suppressed canonical Wnt signaling by upregulating *WNT5A*, an inhibitor of canonical Wnt signaling. MESP1 was reported to bind to the Enhancer Box (E-Box) motif of its target genes 20. There were multiple E-Box motifs in the promoter region of *WNT5A* (Figure 4F). Chromatin immunoprecipitation followed by Q-PCR confirmed that MESP1 indeed bound to E-Box A (Ebox A) located just before the translation start site (TSS) of *WNT5A* (Figure 4G-H). Moreover, luciferase reporter assay showed that when Ebox A was mutated or deleted from the *WNT5A* promoter, the luciferase activity decreased significantly (Figure 4I).

Inducing WNT5A protein expression during mesoderm differentiation day 3 to 5 also down-regulated total β-CATENIN protein (Figure 4J and Figure S4C-D). Finally, we tried to replace IWP2 treatment with WNT5A (Figure 4K). FACS analysis showed that 27.46% of cells were cTnT positive by day 12 (Figure 4L). Collectively, these results demonstrated that MESP1 can repress canonical Wnt signaling in human cardiovascular progenitor cells through directly upregulating the non-canonical Wnt ligand *WNT5A*.

### Optimizing cardiomyocyte, endothelial and smooth muscle cell differentiation using MESP1-mTomato reporter cells

**Figure 5:**
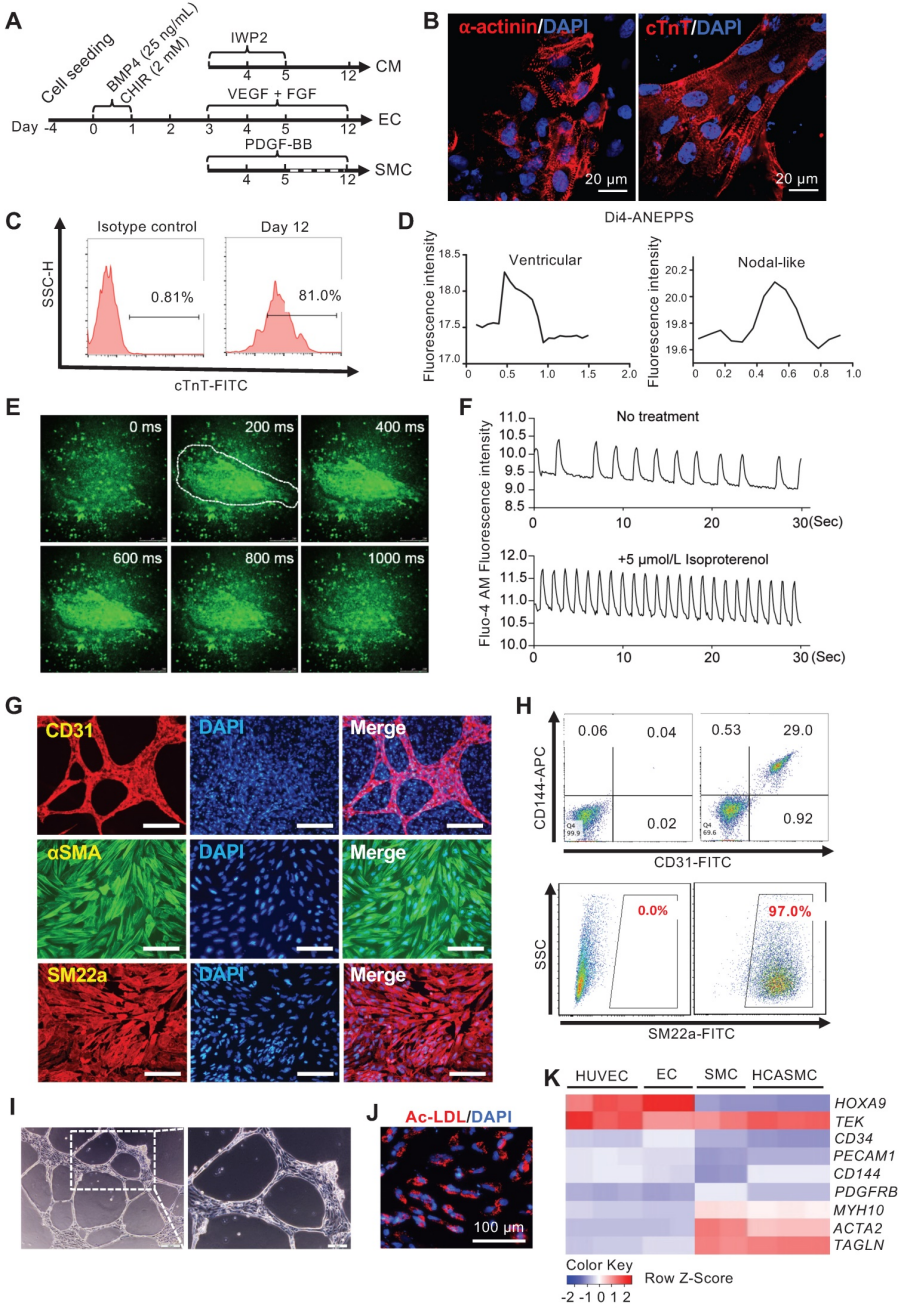
** Efficient tri-lineage differentiation based on optimizing MESP1-mTomato expression.** (**A**) Schematic view of the tri-lineage differentiation system. (**B**) Immunostaining of α-actinin (red) and cTnT (red) in CMs, DNA in blue, scale bars, 20 µm. (**C**) Flow cytometry analysis of cTnT^+^ cells on day 12. (**D**) Di-4-ANEPPS fluorescence intensity measurement to show the action potential change in MESP1-mTomato^+^ cells derived CMs. Most beating cells showed ventricular-like electrophysiology characteristics (left), and some displayed nodal-like potential change (right). (**E**) Time-lapse images of Ca^2+^ transients in CMs. The time was indicated on the top-right corner; the white line circled the contracting area. (**F**) Drug response of CMs differentiated from MESP1-mTomato^+^ cells. Beating cells stained with Fluo-4 AM were first filmed. Then they were treated with 5 µmol/L Isoproterenol and filmed again. Images were recorded at 10 frames per second. Fluorescence intensity of the circled area in (E) was quantified before and after Isoproterenol addition. (**G**) Immunostaining of CD31 (red), αSMA (green) and SM22a (red). Scale bars, 100 µm. (**H**) Flow cytometry analysis of CD31-CD144 for ECs and SM22a for SMCs. (**I**) Tube formation assay of ECs. Scale bar, 10 µm. (**J**) DiI-ac-LDL (red) uptake assay, scale bar, 100 µm. (**K**) Heatmap comparison of marker gene expression among HUVEC, MESP1 cells derived EC, SMC, and HCASMC.

Based on transcriptome analysis and signaling pathway test on MESP1-mTomato cells, we set up a monolayer, tri-lineage differentiation system that can efficiently generate CMs, ECs and SMCs through simple supplement alteration (Figure 5A). HESCs were dissociated to single cells and replated in E8 on Matrigel. When cells reached appropriate concentration (differentiation day 0), the medium was changed to RPMI/B27-I. BMP4 (25 ng/mL) and CHIR99021 (2 μM) were added during the first 24 h to induce more than 90% of cells to turn on mTomato by day 3. On differentiation day 3, the medium was changed to RPMI/B27-I with IWP2 (2 μM) for 48 h, then replaced with RPMI/B27-I. Beating clusters started to appear on day 8. For EC induction, on differentiation day 3, the medium was changed to RPMI/B27 supplemented with VEGF (50 ng/mL) and bFGF (10 ng/mL). To obtain SMCs, RPMI/B27 supplemented with PDGF-BB (10 ng/mL) was used from day 3 (Figure 5A).

In a typical CM differentiation experiment, by day 12, more than 80% of cells were cTnT^+^ (Figure 5C). Confocal imaging of α-actinin and cTnT immunostaining revealed typical sarcomere structures in the CMs (Figure 5B). Many beating areas could be seen in the dish (Movie S2). To characterize the CM subtype, we used a voltage-sensitive dye, Di4-ANEPPS, to monitor the change in the membrane potential. 30 contracting foci were measured. Based on the pattern of Di4-ANEPPS fluorescence intensity change over time, most CMs displayed ventricular-like action potential, and a small number of them showed nodal-like action potential (Figure 5D). Fluo-4 AM dye was used to detect the calcium transient in the contracting foci. A strong, rhythmic calcium influx can be seen after Fluo-4 AM staining (Figure 5E-F, and Movie S3). The contracting foci can respond to Isoproterenol treatment and accelerate the beating rate as normal heart tissue (Figure 5F, and Movie S4). These results confirmed that the CMs derived from MESP1-mTomato cells had a normal physiological function. For EC and SMC differentiation, after CHIR induction and cell replating, VEGF + FGF2 or PDGF-BB, were added. We typically obtain around 30% of CD31^+^ CD144^+^ ECs, or nearly 100% αSMA^+^ SM22a^+^ SMCs by day 12 (Figure 5G-H). In collagen gel, ECs formed typical tubular networks (Figure 5I). They can also uptake DiI-ac-LDL (Figure 5J). We compared marker gene expression in Human Umbilical Vein Endothelial Cells (HUVEC), MESP1 cells derived EC, SMC, and human coronary artery smooth muscle cells (HCASMC). Our ECs and SMCs upregulated lineage specific marker genes similar to primary HUVECs and HCASMCs (Figure 5K). The above results demonstrated that monitoring MESP1 reporter signal and going through a MESP1^+^ cell progenitor stage facilitated highly efficient CM, EC or SMC generation from hESCs.

### Transplantation of MESP1 progenitor cells improved MI repair in rats

**Figure 6:**
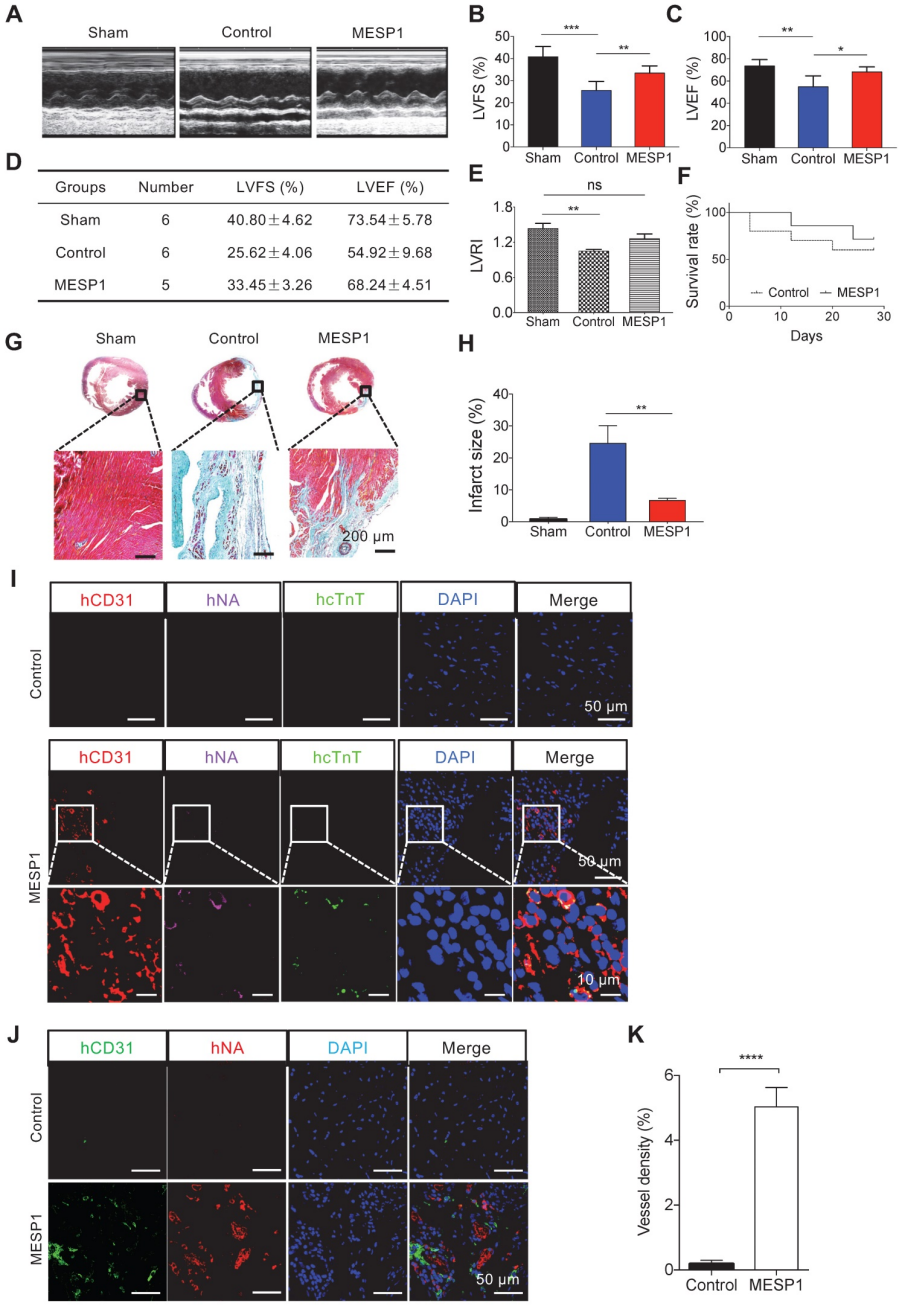
** Transplantation of MESP1^+^ cardiac progenitor cells improved the function of rat MI hearts.** (**A**) Representative M-mode echocardiographic images obtained in Sham, Control, and MESP1 groups. (**B-C**) Quantitative cardiac function analysis of LVFS and LVEF 28 days after permanent LAD occlusion of the three groups. Data expressed as mean ± SD. Sham (*n* = 6), Control (*n* = 6), and MESP1 (*n* = 5). *p < 0.05, **p < 0.005, ***p < 0.001, *t*-test. (**D**) Table summarizing the echocardiography result. (**E**) Distribution of left ventricular remodelling index (LVRI) in Sham, Control, and MESP1 groups. (**F**) The survival curve of rats in the Control (*n* = 10) and MESP1 (*n* = 7) groups. (**G**) Representative images of rat hearts (Masson's trichrome staining) for Sham, Control, and MESP1 groups. (**H**) Infarct size as percent area of myocardial scarring in MI hearts. Data are mean ± SD measured from Masson's trichrome staining, **p < 0.005, *t*-test. (**I and J**) Immunostaining of hCD31, human nuclei (hNA) and human cTnT (hcTnT) 28 days after transplantation; scale bar: 50 µm. The color of the antigen is as indicated on the top, DNA, blue. (**K**) Quantification of vessel density in the myocardial scar and border zone 28 days after implantation based on CD31 immunostaining (*n* = 5 or 6 animals per group). Data are mean ± SEM, ****p < 0.0005.

To explore the reparatory potential of human MESP1^+^ cells *in vivo*, we used a rat model of MI, where the left anterior descending coronary artery (LAD) was ligated to induce ischemia and CM death. After MI, we injected 1 × 10^7^ MESP1^+^ cells along the border zone of the infarcted area. 28 days after transplantation, we performed echocardiography examination. In control MI hearts, there was a significant decrease of left ventricle ejection fraction (LVEF) (54.92 ± 9.68%) and left ventricle fractional shortening (LVFS) (25.62 ± 4.06%) compared to sham group's LVEF (73.54 ± 5.78%) and LVFS (40.80 ± 4.62%). MESP1^+^ cells engraftment significantly increased LVEF (68.24 ± 4.51%) and LVFS (33.45 ± 3.26%) (Figure 6A-D). MESP1 cells also improved the left ventricular remodelling index (LVRI) considerably (Figure 6E). By the end of 4 weeks, 5 out of 7 MESP1 cells transplanted animals survived, compared to 6 out of 10 for control animals (Figure 6F). After echocardiography evaluation, we harvested hearts to do histological analysis. Masson's trichrome staining revealed significantly less fibrotic tissues in MESP1^+^ cells engrafted hearts compared with PBS injected hearts (Figure 6G). There was no fibrosis in sham group hearts (Figure 6H). The area of light blue collagen staining in MESP1^+^ cells engraftment hearts was significantly smaller than that in PBS injected hearts (Figure 6G-H), suggesting significantly less infarction induced pathological remodelling.

Immunostaining analysis of the peri-infarct area revealed that transplanted MESP1^+^ cells formed CD31^+^ capillaries, judging by co-localization of a human-specific CD31 antibody and a human-specific nuclei antibody (Figure 6I-K and Figure S5). In MESP1^+^ cells engraftment and sham hearts, CMs were better aligned, and the intensity of β-CATENIN was significantly stronger than that in infarcted hearts (Figure S6), suggesting better remuscularization and improved physiological functions. It is consistent with better remuscularization and physiological function of MESP1^+^ cells engrafted hearts. However, we only detected sporadic human cTnT signal (Figure 6I-J), indicating that very few MESP1^+^ cells differentiated to CM in MI hearts. These results suggest that transplantation of MESP1^+^ cells prevented massive CM death, promoted angiogenesis in the infarct area, and reduced fibrosis; hence the physiological function of the heart is much better preserved.

## Discussion

In this study, we generate a MESP1 knock-in reporter hESC line, which can be a useful tool to study cardiovascular cell fate decisions. In our MESP1 reporter line, mTomato fluorescence peaked from day 3 to 5 even after adding Wnt inhibitor IWP2, suggesting that once activated, MESP1 maintenance no longer needs canonical Wnt signaling. In comparison, in the den Hartogh's study, the MESP1-mCherry was expressed on differentiation day 3 then quickly down-regulated 6,7. This discrepancy may due to several reasons: (1) our homozygous knock-in cells may be more sensitive and display stronger fluorescence. MESP1 knock-down in human iPSC has been shown to compromise endothelial lineage specification 21. We did not destroy any MESP1 allele in our reporter line, so it should have full range of cardiovascular differentiation potential; (2) in our experiments, we used BMP4 and CHIR in a RPMI1640/B27 based chemically defined medium, while in Den Hartogh's study, Activin A was also added; (3) the genetic background of the parental hESC lines (H9 in our case and HES3 for MESP1mCherry/w-NKX2-5eGFP/w dual reporter) may also lead to some differences. Nevertheless, our transcriptome and cell surface markers analysis convincingly proved that these MESP1^+^ cells were mesoderm cells with strong cardiovascular differentiation potential. This line could be genetically modified to make double-reporter lines, as recently shown by Zhang et al., to study the dynamics of cardiac cell fate decisions 22.

MESP1 appeared to have different target genes in the mouse and human system. In mouse ESCs, overexpressing MESP1 drives cardiovascular differentiation through directly inducing Dkk1 23. In hESCs, WNT5A expression rise significantly following ectopic MESP1 induction, while the DKK1 level decreased, indicating that MESP1 did not activate DKK1 to inhibit canonical Wnt signaling. Previous research showed that the activation of non-canonical Wnt pathway may antagonise the canonical Wnt/β-CATENIN pathway 19. During mouse embryo cardiogenesis, Wnt5a^-/-^; Wnt11^-/-^ double mutant had a severe defect in myocardial differentiation from the second heart field progenitor cells due to prolonged activation of the canonical Wnt signaling 24. Experiments in mouse ESCs also found that Wnt5a and Wnt11 treatment can suppress the activity of the canonical Wnt/β-CATENIN pathway 24, and combined Wnt5a and Wnt11 treatment significantly increased the expression of key cardiac transcription factors Isl1, Tbx5 and Nkx2.5 24. In our RNA-seq analysis, among all the Wnt genes in MESP1-mTomato^+^ cells, *WNT5A* was the one upregulated the most on both day 3 and day 5, and its mRNA increased nearly 5 folds by ectopic MESP1 expression (Figure 4D). Moreover, ChIP-Q-PCR experiments revealed that MESP1 directly bound to the E-Box motif in the *WNT5A* promoter. Together, these results strongly suggest that *WNT5A* is a direct target of MESP1 in human cardiovascular progenitor cells and contributed to the repression of the canonical Wnt pathway during *in vitro* cardiomyocyte differentiation. Our study revealed the molecular mechanism underlying the biphasic regulation of canonical Wnt signaling during CM differentiation. A strong canonical Wnt signaling activates *MESP1* expression. Afterward, MESP1 induces *WNT5A* expression and activates the non-canonical Wnt signaling to suppress the canonical Wnt signaling. Thus, MESP1 appeared to initiate a negative feedback loop to scale down canonical Wnt signaling during the second stage of cardiomyocyte differentiation.

Compared to MESP1^-^ cells, MESP1^+^ cells highly expressed endothelial progenitor genes and angiogenesis genes such as *FLT1, KDR, VEGFA, HGF,* etc*.* (Figure 3C). A high percentage of MESP1^+^ cells could also be a useful indicator of strong endothelial and smooth muscle differentiation potential. We obtained about 30% of EC, or more than 95% of SMC (Figure 5G-H). Recently, Hamad and co-workers demonstrated that a scalable 3D suspension bioreactor culture enabled the generation of a large amount of CMs from human iPSCs 25. A similar system may be adapted to use with our MESP1 reporter cells to produce large quantities of CMs, ECs, and SMCs for regenerative medicine.

Human pluripotent stem cell-derived mesoderm cells with strong cardiac differentiation bias have been used in transplantation experiments to regenerate infarcted hearts 26,27. These cells are marked by the stage-specific embryonic antigen 1 (SSEA1) surface epitope. Compared to SSEA1 cells, MESP1 reporter cells provided a more defined cell population. In Blin et al.'s report, engrafted SSEA1 cells (2 × 10^7^) formed CM in MI Rhesus monkey hearts 27, while in a similar transplantation study carried out by Zhu and colleagues, they transplanted 1 × 10^7^ SSEA1 cells to the MI hearts of male cynomolgus monkey (5-8 years old), and found few cells left after 28 days 26. This discrepancy may be due to many factors. For example, Blin et al.'s infarction procedure were 90 minutes, followed by reperfusion, and 2 × 10^7^ SSEA1 cells were implanted 26. While in Zhu et al.'s study, permanent LAD ligation was done to the hearts, and 1 × 10^7^ SSEA1 cells were engrafted. Therefore, in Zhu's case, the ischemia in the MI hearts may be more severe and extensive, create a very hostile microenvironment for cell survival 26. In our transplantation test using a rat MI model after permanent LAD ligation, 1 × 10^7^ MESP1^+^ cells were injected. After 28 days, echocardiography measurement of LVEF and LVFS revealed that MESP1 engrafted hearts had much better physiological function compared to control hearts. The LVRI score had no significant difference from that of the Sham group. MESP1^+^ cells engrafted hearts have significantly less fibrosis and a more muscularized area, which is in line with the much better physiological function of this group (Figure 6A-H). In our case, we observed only a small number of graft-derived CMs, and more graft-derived ECs in receipt hearts, this result suggests that the microenvironment of adult rat MI hearts does not favor CM differentiation of human MESP1^+^ cells while permitting more EC differentiation (Figure 6I-K). The EC differentiation and blood vessel formation from engrafted human MESP1^+^ cells were also observed when they were injected into a mouse model of the hindlimb ischemia 28. These observations confirmed that MESP1^+^ cells have strong angiogenic potential in an ischemia environment. On the other hand, robust CM formation *in vivo* is likely to require more specific induction signals. Tissue-engineered cardiac patch incorporated with human iPSC derived CM, EC, and SMCs have been shown to nicely repair porcine model of acute MI without adverse complications 29. As MESP1^+^ cells have strong potential to rapidly differentiate to CM, EC and SMC *in vitro*, in the future, tissue-engineered combinatory scaffolds with lineage-specific induction cues may be created to deliver MESP1^+^ cells into the infarcted heart to facilitate simultaneous CM and EC differentiation, while achieving better cardiac repair. Alternatively, if one concerns about the safety and immunocompatibility of cell transplantation, extracellular vesicles secreted by stem cells contains various proteins, lipids, and nucleic acids, and possess cardiac repair and regenerative potential 30. A recent study showed that the incorporation of small extracellular vesicles (VE) in sodium alginate hydrogel increased their retention in the infarcted heart and improved therapeutic effects 31. MESP1 cells highly expressed several potent pro-angiogenesis factors, including *VEGFA, HGF* 30. It would be interesting to characterize VEs released by MESP1 cells and test their reparatory potential by embedding them in hydrogels to treat MI.

Summed together, our work provided a detailed characterization of MESP1 cardiovascular progenitor cells in the human system, revealed the molecular mechanism responsible for the biphasic regulation of canonical Wnt signaling during human CM *in vitro* differentiation, and demonstrated the therapeutic potential of MESP1^+^ cells in treating MI. MESP1-mTomato reporter hESCs and the MESP1 inducible expression system generated in this study will be valuable platforms to study the mechanism regulating human mesoderm cell fate decisions, which can be further explored for cardiovascular regeneration therapies.

## Supplementary Material

Supplementary figures.

Supplementary movies.

## Abbreviations

MESP1: mesoderm posterior 1
HSCs: hematopoietic stem cells
hPSCs: human pluripotent stem cells
ESCs: embryonic stem cells
iPSCs: induced pluripotent stem cells
CM: cardiomyocyte
EC: endothelial cell
SMC: smooth muscle cell
MI: myocardial infarction
TALEN: transcription activator-like effector nuclease
UTR: untranslated region
RA: retinoic acid
PCA: principal component analysis
GO: gene ontology
GSEA: gene set enrichment analysis
Dox: doxycycline
E-Box: enhancer box
ChIP: chromatin immunoprecipitation
TSS: translation start site
Q-PCR: quantitative polymerase chain reaction
FACS: fluorescent-activated cell sorting
HUVEC: human umbilical vein endothelial cells
HCASMC: human coronary artery smooth muscle cells
LAD: left anterior descending coronary artery
LVEF: left ventricle ejection fraction
LVFS: left ventricle fractional shortening
LVRI: left ventricular remodelling index
PBS: phosphate buffer saline
SSEA1: stage-specific embryonic antigen 1
VE: extracellular vesicles
SEM: standard error of the mean
GEO: gene expression omnibus
NSFC: National Natural Science Foundation of China
MEF: mouse embryonic fibroblast
mTomato: membrane-bound tdTomato
PGK-Puro: PGK promoter driving puromycin resistance gene
FPKM: fragments per kilobase million
DEGs: differentially expressed genes
KEGG: Kyoto Encyclopedia of Genes and Genomes
PB: PiggyBac
IRES: internal ribosomal entry site
LVEDD: left ventricle end diastolic dimension
LVESD: left ventricle end-systolic dimension
LVEDV: left ventricular end-diastolic volume
LVM: left ventricular mass
ANOVA: analysis of variance
pA: polyadenylation sequence
PGK: phosphoglycerate kinase promoter
RFP: red fluorescence protein
MC1-TK: MC1 promoter driving TK gene
RVD: repeat variable di-residue
WT: wild type
BF: bright field
MT: MESP1-mTomato
HE: hematoxylin and eosin
GFP: green fluorescence protein
