# Supplementary material for: Homozygous MESP1 knock-in reporter hESCs facilitated cardiovascular cell differentiation and myocardial infarction repair
Source: Theranostics. 2020 May 23;10(15):6898–914. doi: 10.7150/thno.42347 (PMC7295063; doi:10.7150/thno.42347)
Supplement: Supplementary file 1 — Supplementary figures. [file thnov10p6898s1.pdf]

**Figure S1. mTomato gene was targeted into the *MESP1* gene locus correctly in H9 cells**

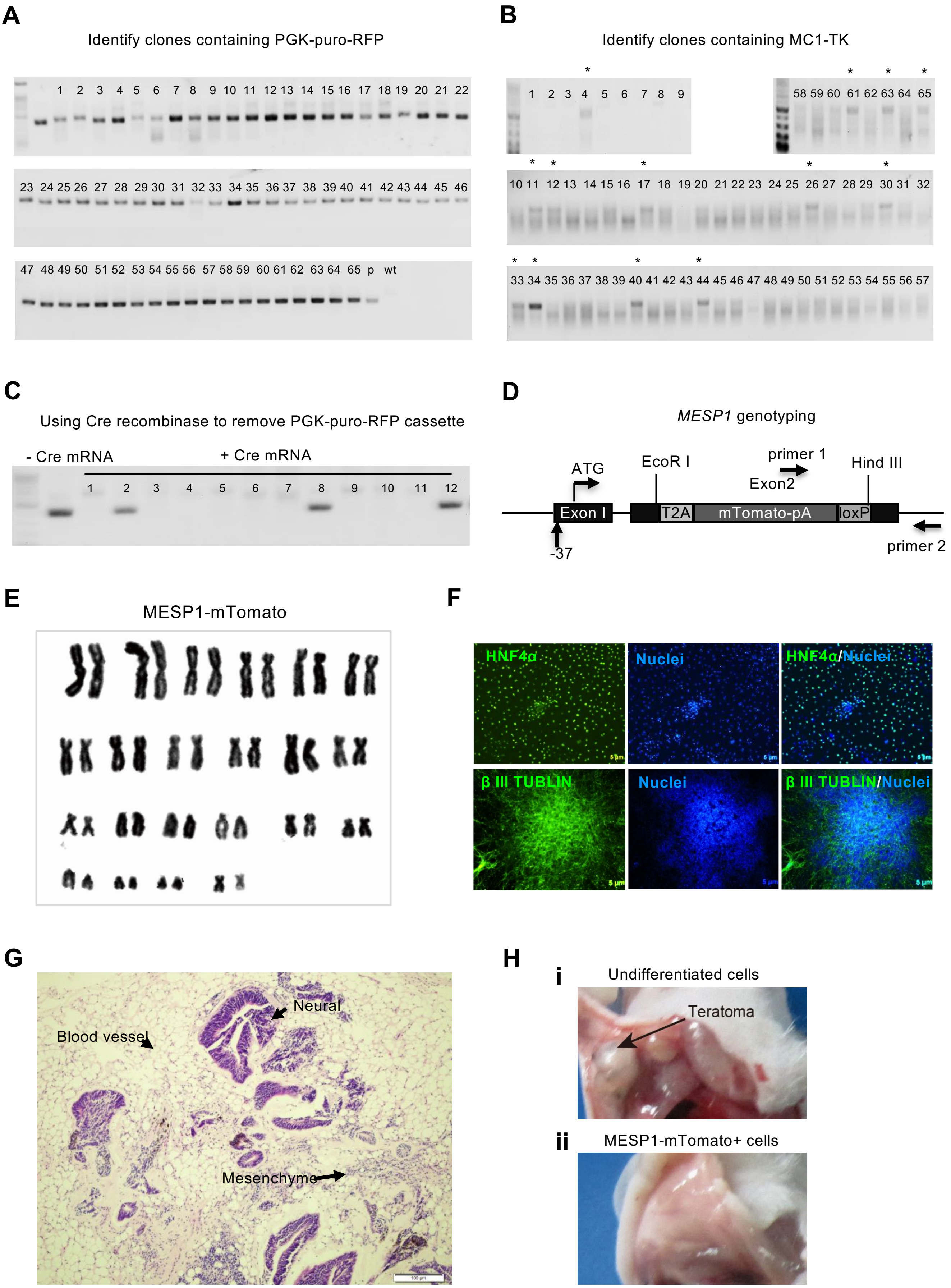

**Figure S1. mTomato gene was targeted into the *MESP1* gene locus correctly in H9 cells.**
(A) After transfection and selection, 65 puromycin resistant hESC clones all contained the PGK-puroRFP cassette. pc, plasmid positive control; wt, wild-type genomic DNA. (B) Genomic DNA PCR to analyze random insertion. Out of 65 clones, 13 clones had MC1-TK sequence, indicating random insertion "*". The rest clones did not contain MC1-TK cassette, suggesting correct targeting. (C) PGK-puroRFP cassette removal. Of the 12 clones examined, PGK-puroRFP was removed from 9 clones. (D) Positions of genomic DNA PCR primer pair used in A-C. The forward primer is within T2A-mTomato sequence; the reverse primer is on the 3′ homologous arm. (E) MESP1-mTomato reporter cells had a normal 46 XX diploid karyotype. (F) MESP1-mTomato cells can differentiate into endoderm and neuroectoderm derivatives. Upper panels, immunostaining of endoderm transcription factor HNF4a (green); lower panels, immunostaining of neural marker gene βIII Tubulin (green), DNA (blue). (G) Undifferentiated MESP1-mTomato H9 can form well-differentiated teratoma upon transplantation into nude mice. Arrows indicate neural, vascular and mesenchyme tissues (HE staining). (H) MESP1-mTomato$^+$ cells do not have tumorigenicity. i. well-differentiated teratoma formed by undifferentiated MESP1 reporter cells ($n$ = 4). ii. No tumor was detected in mTomato$^+$ cells injected mice ($n$ = 4).

**Figure S2. Heatmap of marker gene expression in MESP1-mTomato⁺ cells.**

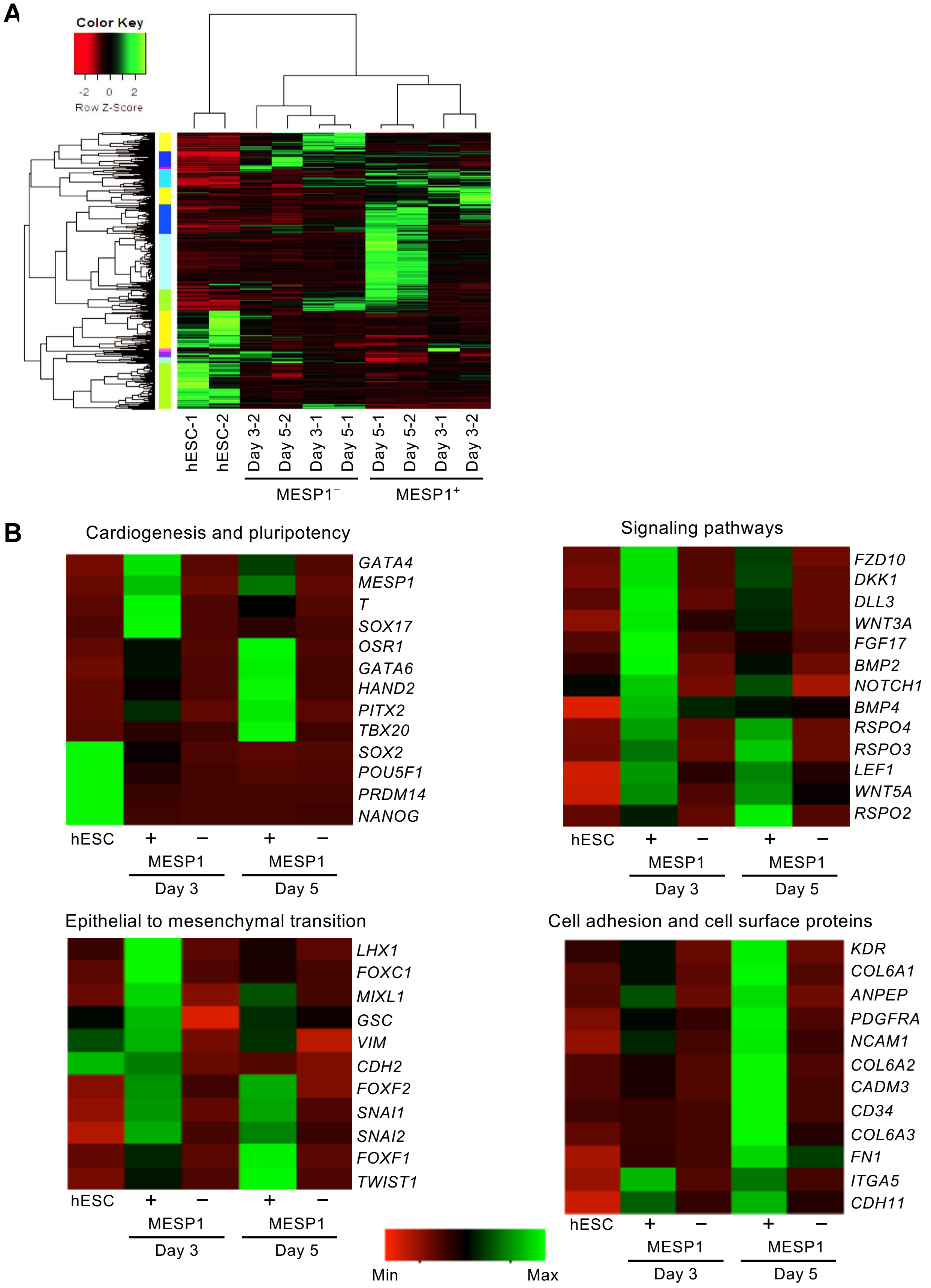


**Figure S2. Heatmap of marker gene expression in MESP1-mTomato⁺ cells.**
(A) Heatmap clustering of the transcriptome of undifferentiated H9, day 3 and 5 MESP1⁺ and MESP1⁻ cells. (B) Heatmap of selective gene expression in undifferentiated hESCs, differentiation day 3 and day 5 mTomato⁺ and mTomato⁻ cells. mTomato⁺ cells enriched genes include transcription factors involved in cardiogenesis, signaling pathway ligands and receptors, epithelial to mesenchymal transition-related genes, specific cell surface proteins. Selected genes with a fold change ≥ 1.5 in mTomato⁺ cells versus mTomato⁻ cells are shown.

**Figure S3. Cell death related gene expression in MESP1-mTomato⁺ cells.**

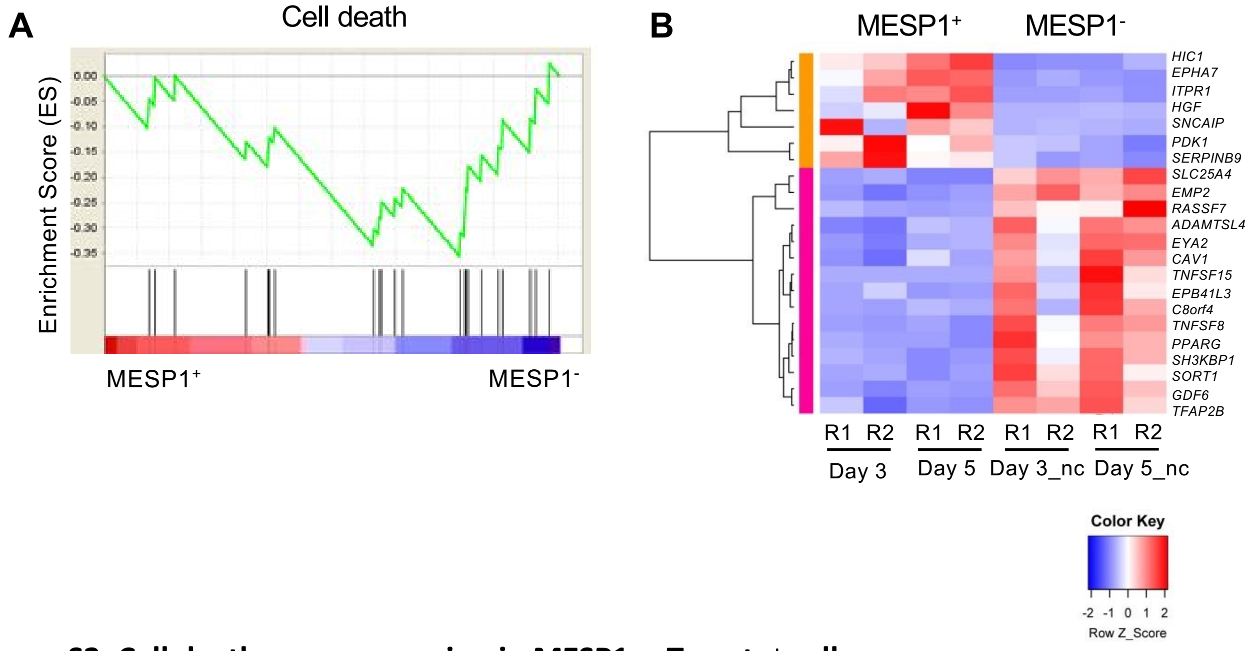


**Figure S3. Cell death gene expression in MESP1-mTomato⁺ cells.**
(A) GSEA analysis of cell-death related genes in MESP1⁺ cells and MESP1⁻ cells. (B) Heatmap of selective cell-death related gene expression in differentiation day 3 and day 5 MESP1⁺ and MESP1⁻ cells.

## Figure S4. Quantification of western blot in Figure 4.

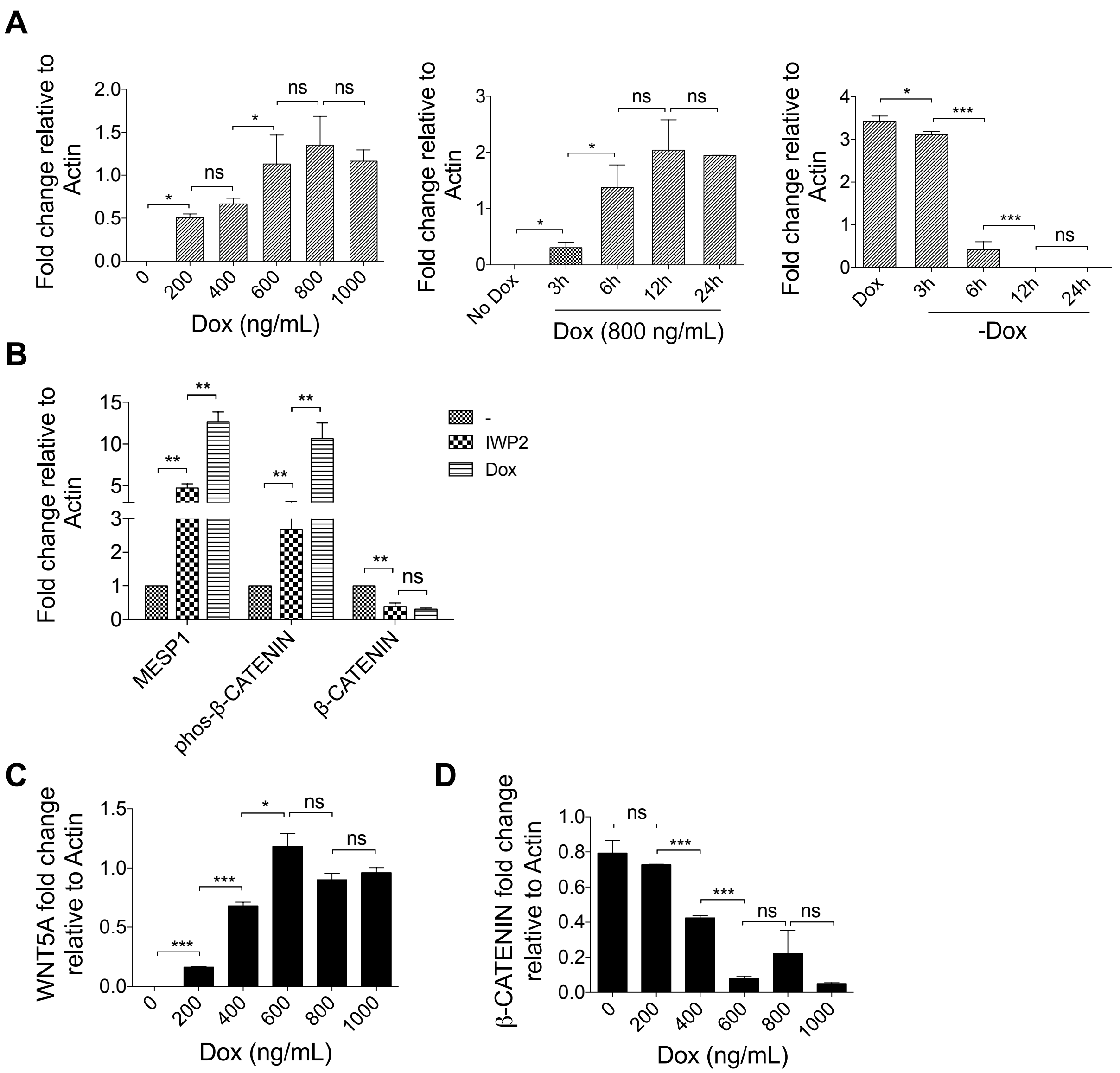


**Figure S4. Quantification of western blots in Figure 4.**

(A) Quantification of the MESP1-Flag bands in Figure 4A, the relative levels were normalized to ACTIN. (B) Quantification of MESP1, b-CATENIN, Phos-b-CATENIN proteins in Figure 4E, the relative levels were all normalized to ACTIN. None, IWP2, or Dox treatment was as indicated. (C) Quantification of WNT5A and β-CATENIN proteins in Figure 4J, the relative levels were all normalized to ACTIN.

**Figure S5. Larger blood vessels formed in MESP1 cells engrafted rat MI hearts.**

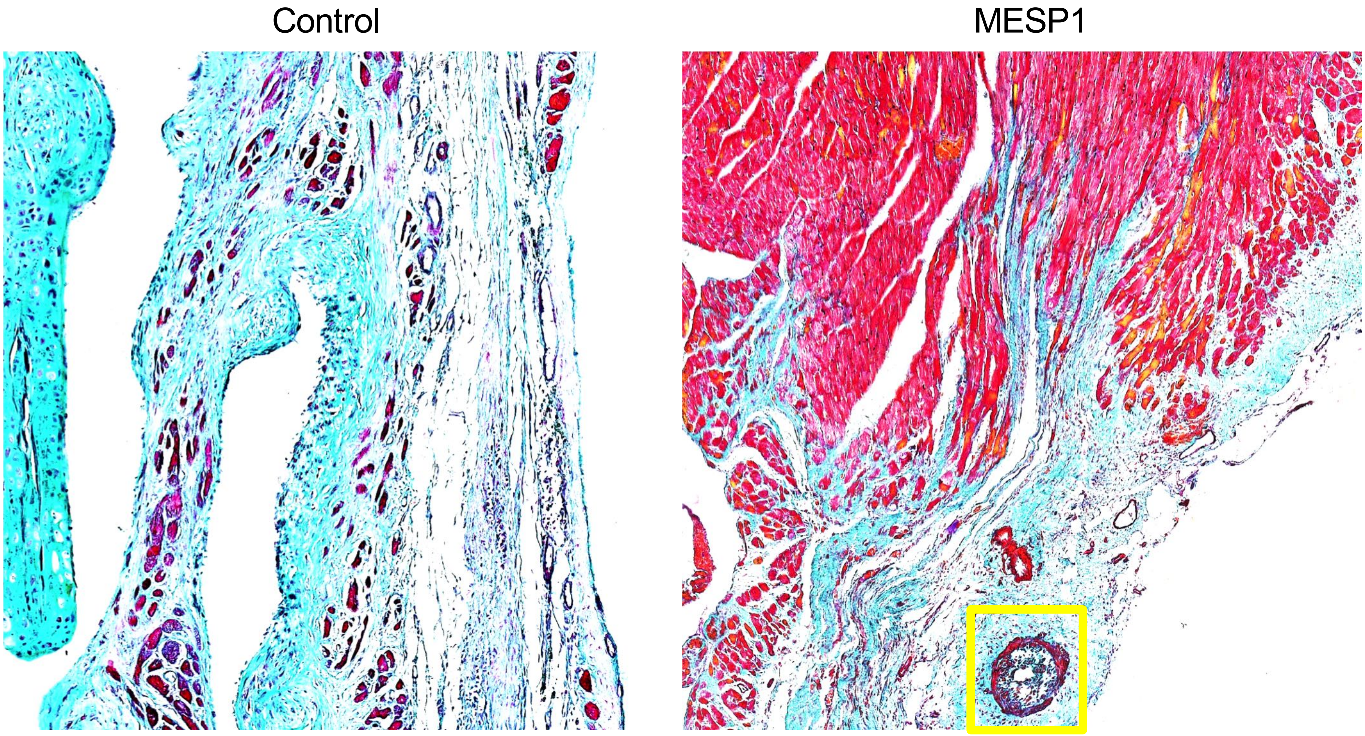


**Figure S5. Blood vessels in MESP1 cells engrafted rat MI hearts.**
Enlarged histological section and Masson's trichrome staining of control and MESP1 cells transplanted hearts. Yellow square highlights a large blood vessel, and black arrows pointing to smaller blood vessels in the MESP1 group.

**Figure S6. Immunostaining and quantification of β-CATENIN expression in rat heart 28 days after transplantation.**

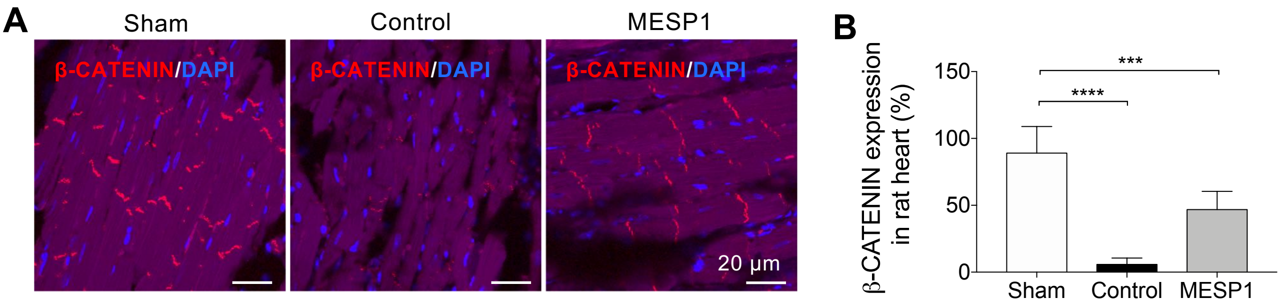


**Figure S6. Immunostaining analysis and quantification of β-CATENIN expression (percent area) in rat heart 28 days after transplantation.**
(A) Immunostaining of β-CATENIN (red) on histological sections of rat hearts 28 days post engraftment. DNA (blue). (B) Quantification of β-CATENIN fluorescence signal ($n$ = 5 or 6 animals per group). Data are mean ± SEM, ***$p < 0.001$, ****$p < 0.0005$.

## Supplemental Information

# Homozygous MESP1 knock-in reporter hESCs facilitated cardiovascular cell differentiation and myocardial infarction repair

Lin Wang[1,#], Fengzhi Zhang[1,#], Fuyu Duan[1], Rujin Huang[1], Xi Chen[1], Jia Ming[1] and Jie Na[1,*]

[1]Center for Stem Cell Biology and Regenerative Medicine, School of Medicine, Tsinghua University, Beijing, 100084, China

## Supplemental Materials and Methods

### Verification of MESP1-mTomato knocking-in reporter cell line

Genomic DNA of individual MESP1-mTomato clones was extracted. PCR was performed to detect correct *MESP1* gene targeting and Cre mediated PGK-Puro cassette removal. The primer sequences were listed in Table S4 and the results were shown in Figure S1. The identity of the PCR products was verified by Hind III and EcoR I digestion. PCR primers used to verify mTomato knock-in were listed in Table S4.

### Teratoma formation assay

All animal experiments were conducted following the Guide for the Care and Use of Animals for Research Purposes. The protocol for teratoma formation in nude mice was approved by the Institutional Animal Care and Use Committee and Internal Review Board of Tsinghua University. For teratoma assay, $5 \times 10^6$ undifferentiated MESP1-mTomato hESC or purified mTomato$^+$ cells in 100 μL medium were mixed with 100 μL Matrigel and injected subcutaneously into nude mice. 4 weeks later, mice were sacrificed, hESC formed teratomas were dissected, fixed in 4% paraformaldehyde, embedded in paraffin, sectioned and stained with hematoxylin and eosin (HE) dye.

### WNT5A promoter luciferase reporter assay

Plasmids were constructed using Seamless Assembly Cloning Kit (Taihe Biotechnology, Co LTD). Wild-type *WNT5A* promoter containing E-box regulatory elements was cloned from hESC genomic DNA and inserted between Nhe I and Bgl II sites of the Firefly luciferase reporter plasmid (PGL-basic vector). Reporter plasmids containing mutated E-box motif was constructed by PCR. For Mut E-box A, the E-box motif "CATCTG" was mutated to "TGTCTG" through primer design and overlapping PCR. Primers used to generate luciferase reporters were list in Table S5.

Luciferase reporter assay was performed in 293FT cells. Cells were seeded in 24-well plates and transfected with CAG-HA-MESP1 (0.8 μg), pRL-TK (0.1 μg), and WNT5A E-box plasmids (0.8 μg) using Lipofectamin2000 (Invitrogen). 48 h after transfection, cells were harvest and luciferase activity was analyzed using Dual Luciferase Reporter Gene Assay Kit (Beyotime). Renilla luciferase activity was used to normalize the test reporter activity. The value of the empty pGL vector activity was set as "1". Luciferase activity was measured in a FLASH microplate reader SP-Max 1800L.

**Supplementary Tables**

**Table S1. Primers for Q-PCR**

| Gene | Accession No. | Forward primer | Reverse primer | $T_m$ |
|---|---|---|---|---|
| *KDR/FLK1* | NM_002253 | tgagcaaagggtggaggtgact | cttgcacaaagtgacacgttgag | 60 |
| *GAPDH* | NM_002046 | tgatgacatcaagaaggtggtgaag | tccttggaggccatgtgggccat | 60 |
| *GATA4* | NM_002052 | gaaaacggaagcccaagaacct | ccagacatcgcactgactgag | 60 |
| *HAND1* | NM_004821 | gcgagtgcatccccaacgtg | gaggaaaaccttcgtgctgct | 60 |
| *HAND2* | NM_021973 | tcagaagaccgacgtgaaa | gttgctgctcactgtgcttt | 60 |
| *ISL1* | NM_002202 | ctgcacaccttgcggacctg | caccgtcgtgtctctctggac | 60 |
| *MEF2C* | NM_001131005 | cagtct ccatcccagtgtcca | gcctgcaccagacgtgaggt | 60 |
| *MESP1* | NM_018670 | cgctgtgccccgacgact | ggcatccaggtctccaacag | 60 |
| *NKX2.5* | NM_004387 | aaggaccctagagccgaaaag | gccgctccagctcatagacc | 60 |
| *PAX6* | NM_000280 | aattcgtggcaaagcttgtt | actggggaaggaatggactt | 60 |
| *PDGFR A* | NM_006206 | acaggttggtgtgggtccat | ctgcatcttccaaagcatca | 60 |
| *TBX5* | NM_000192 | aggcggatgtttcccagttac | aatgggtccaggtggttgttg | 60 |

| | | | | |
|---|---|---|---|---|
| *WNT5A* | NM_001256105 | cggcatctctctttcaccat | aatgcaaataggcacgaagg | 60 |
| *WNT3A* | NM_033131 | acgggacgagaggcttctat | ccacaccgtcaggtactcct | 60 |
| *AXIN2* | NM_004655 | aacccccaaaatgttgatga | ggagtgcgttcatggtttct | 60 |
| *DKK1* | NM_012242 | ggagtgcggcactgatgagt | ggtttcctcaatttctcctcgg | 60 |

**Table S2. Primers for ChIP-Q-PCR**

Coverage regions of primers and conserved E-box sites are indicated relative to the ATG for *WNT5A* gene.

| Fragment | Primers | Region 5′/ 3′ | Ebox position 5′/ 3′ |
|---|---|---|---|
| WNT5A-A | Forward: aattatctgagaggcaataaacat<br>Reverse: ctaggaagaacttggaagacatt | 36 / -86 | -60 / -65 |
| WNT5A-B | Forward: tccttctcccctccctagtct<br>Reverse: ggaaatgcagctccgaattaac | -654 / -793 | -648 / -653<br>-726 / -731 |
| WNT5A-C | Forward: aaccccagatcaggaccaag<br>Reverse: cccaaattctgatttttcactct | -995 / -1107 | -1031 / -1036<br>-1038 / -1043 |
| WNT5A-D | Forward: ccaactcctacccctgaaattc<br>Reverse: agagcccttgcagatgagtcc | -1241 / -1376 | -1251 / -1256<br>-1294 / -1299<br>-1421 / -1426 |
| Unrelated region | Forward: cgtgccccatttcccgtgcg<br>Reverse: gaagcctggtgcgtaagccg | Genomic fragment | - |

**Table S3. Genes in cluster I-II-III**

Figure 2E cluster I genes

| Genes | hESC | | Day 3 MESP1$^+$ | | Day 3 MESP1$^-$ | | Day 5 MESP1$^+$ | | Day 5 MESP1$^-$ | |
|---|---|---|---|---|---|---|---|---|---|---|
| | R1 | R2 | R1 | R2 | R1 | R2 | R1 | R2 | R1 | R2 |
| ACOXL | 5.74 | 5.52 | 0.78 | 0.18 | 0.39 | 0.27 | 0.28 | 0.25 | 0.33 | 0.30 |
| ACTG2 | 39.90 | 5.23 | 1.30 | 0.31 | 4.01 | 3.73 | 1.22 | 0.54 | 3.91 | 5.83 |
| ADD2 | 47.42 | 26.42 | 9.61 | 18.24 | 4.99 | 5.29 | 11.23 | 15.35 | 3.27 | 1.90 |
| ADM | 95.83 | 10.32 | 10.49 | 4.25 | 15.59 | 17.61 | 3.59 | 5.87 | 16.05 | 25.07 |
| AK4 | 30.68 | 6.47 | 3.21 | 3.19 | 2.20 | 2.63 | 7.38 | 7.80 | 1.70 | 1.09 |
| AKNA | 7.53 | 8.11 | 1.93 | 2.78 | 7.70 | 1.64 | 2.22 | 3.20 | 8.78 | 5.74 |
| ALDH3A2 | 31.58 | 27.13 | 10.83 | 10.32 | 10.05 | 16.33 | 13.88 | 13.46 | 8.83 | 6.96 |
| ANO5 | 1.66 | 1.32 | 0.32 | 0.29 | 0.67 | 0.70 | 0.51 | 0.35 | 0.65 | 0.18 |

| | | | | | | | | | | |
|---|---|---|---|---|---|---|---|---|---|---|
| AP1S2 | 54.33 | 53.57 | 19.00 | 19.55 | 27.33 | 27.23 | 36.71 | 29.83 | 25.85 | 11.35 |
| ARHGAP23 | 15.94 | 6.78 | 4.83 | 3.19 | 8.17 | 5.91 | 7.06 | 6.73 | 9.73 | 7.60 |
| ARNTL2 | 5.12 | 3.53 | 0.89 | 1.20 | 0.99 | 1.12 | 0.57 | 0.67 | 0.87 | 0.45 |
| ATP8A2 | 1.90 | 2.37 | 0.16 | 0.30 | 0.36 | 0.11 | 0.22 | 0.07 | 0.17 | 0.09 |
| ATPAF1 | 33.92 | 41.02 | 14.09 | 12.66 | 18.43 | 29.29 | 20.18 | 20.33 | 19.38 | 17.45 |
| AUTS2 | 30.34 | 22.02 | 9.72 | 7.73 | 10.69 | 6.31 | 5.92 | 7.46 | 10.50 | 3.69 |
| B3GALT5 | 4.37 | 0.47 | 0.32 | 0.30 | 0.99 | 0.09 | 0.96 | 0.41 | 1.59 | 0.18 |
| BCOR | 35.91 | 14.34 | 9.51 | 8.23 | 6.63 | 5.20 | 9.56 | 11.15 | 6.65 | 1.28 |
| BEND3 | 20.78 | 12.04 | 3.11 | 5.11 | 3.29 | 3.47 | 2.25 | 2.77 | 3.03 | 1.61 |
| BEND4 | 4.62 | 5.13 | 0.81 | 0.76 | 0.88 | 0.70 | 0.38 | 0.36 | 0.30 | 0.10 |
| C1orf94 | 6.85 | 3.47 | 0.49 | 0.42 | 0.29 | 0.06 | 0.87 | 0.06 | 0.07 | 0.15 |
| C21orf88 | 11.47 | 3.72 | 0.54 | 0.56 | 2.60 | 0.98 | 2.85 | 1.02 | 2.81 | 1.09 |
| CALN1 | 1.02 | 0.93 | 0.08 | 0.19 | 0.06 | 0.08 | 0.10 | 0.08 | 0.06 | 0.02 |
| CAV1 | 22.31 | 2.37 | 1.72 | 0.38 | 10.95 | 5.91 | 5.28 | 2.62 | 15.53 | 11.42 |
| CD200 | 28.04 | 19.74 | 8.23 | 8.92 | 4.60 | 6.71 | 5.34 | 5.24 | 3.03 | 1.89 |
| CD24 | 1353.00 | 1136.71 | 273.03 | 344.86 | 537.84 | 463.48 | 249.03 | 303.48 | 505.26 | 368.14 |
| CDCA7L | 40.80 | 34.40 | 6.84 | 5.83 | 5.41 | 6.93 | 3.23 | 3.66 | 4.48 | 4.39 |
| CELF2 | 7.25 | 5.85 | 3.05 | 1.42 | 3.04 | 2.37 | 1.11 | 1.08 | 3.24 | 0.95 |
| CHODL | 7.33 | 2.85 | 0.57 | 0.34 | 0.30 | 0.20 | 0.62 | 0.14 | 0.26 | 0.24 |
| CHRDL1 | 3.90 | 3.73 | 0.91 | 0.20 | 0.61 | 0.59 | 0.26 | 0.10 | 0.40 | 0.06 |
| CHST7 | 13.59 | 7.30 | 2.69 | 2.32 | 1.96 | 3.22 | 1.66 | 2.04 | 1.27 | 1.44 |
| CNTNAP3 | 3.95 | 4.34 | 0.74 | 0.24 | 0.89 | 0.49 | 0.40 | 0.50 | 0.50 | 0.18 |
| COBL | 8.27 | 4.26 | 1.87 | 1.25 | 3.84 | 2.11 | 2.71 | 2.45 | 2.41 | 1.12 |
| COL7A1 | 12.51 | 8.85 | 3.48 | 1.83 | 4.52 | 2.47 | 2.74 | 3.34 | 4.62 | 3.76 |
| CPT1A | 11.35 | 1.43 | 0.47 | 1.16 | 0.33 | 0.15 | 0.82 | 0.35 | 0.26 | 0.16 |
| CSMD2 | 1.23 | 1.13 | 0.24 | 0.23 | 0.23 | 0.07 | 0.39 | 0.10 | 0.07 | 0.03 |
| CUZD1 | 11.36 | 12.20 | 1.81 | 1.81 | 0.63 | 0.00 | 0.88 | 0.35 | 0.80 | 0.48 |
| CYP2S1 | 56.90 | 43.08 | 9.28 | 3.45 | 10.97 | 4.42 | 4.64 | 4.06 | 10.40 | 9.74 |
| CYP2U1 | 4.30 | 3.63 | 1.28 | 1.12 | 1.11 | 1.35 | 1.84 | 1.33 | 1.09 | 0.48 |
| D21S2088E | 13.75 | 1.46 | 0.15 | 0.62 | 0.16 | 0.07 | 0.39 | 0.15 | 0.11 | 0.04 |
| DBC1 | 17.80 | 3.33 | 1.68 | 1.54 | 1.56 | 0.97 | 1.91 | 0.71 | 1.68 | 0.98 |
| DBNDD1 | 26.94 | 29.32 | 3.91 | 1.41 | 5.30 | 6.16 | 1.01 | 1.15 | 2.27 | 3.38 |
| DNAJB5 | 32.16 | 30.50 | 11.56 | 11.56 | 9.95 | 12.43 | 10.71 | 10.52 | 7.65 | 6.41 |
| DNMT3B | 316.16 | 180.52 | 28.72 | 54.35 | 27.24 | 18.64 | 19.14 | 30.45 | 19.68 | 12.00 |
| DPPA4 | 235.28 | 108.76 | 33.03 | 43.17 | 35.45 | 36.73 | 26.34 | 29.68 | 31.98 | 17.99 |
| DPYSL3 | 204.77 | 248.61 | 75.43 | 45.02 | 84.87 | 44.95 | 25.22 | 29.97 | 111.69 | 68.55 |
| ERBB2 | 130.42 | 68.36 | 18.41 | 22.81 | 35.85 | 41.03 | 21.78 | 27.14 | 29.56 | 29.81 |
| ESRG | 350.14 | 164.32 | 20.33 | 11.13 | 12.46 | 3.73 | 2.78 | 4.19 | 8.22 | 3.98 |
| ESYT3 | 2.05 | 0.95 | 0.12 | 0.21 | 0.50 | 0.14 | 0.42 | 0.12 | 0.39 | 0.12 |
| ETV1 | 16.09 | 5.88 | 1.52 | 2.23 | 0.73 | 1.30 | 1.21 | 1.64 | 0.75 | 0.22 |
| ETV4 | 43.25 | 8.22 | 7.69 | 4.38 | 2.87 | 6.53 | 2.30 | 3.22 | 3.28 | 5.52 |
| FAM124A | 10.38 | 11.40 | 0.97 | 1.56 | 0.56 | 0.48 | 1.10 | 0.65 | 0.45 | 0.31 |

| | | | | | | | | | | |
|---|---|---|---|---|---|---|---|---|---|---|
| FAM129A | 4.80 | 2.31 | 0.93 | 0.63 | 2.57 | 0.13 | 1.00 | 0.46 | 5.42 | 2.01 |
| FAM155B | 4.97 | 5.96 | 1.23 | 1.24 | 0.73 | 0.55 | 0.34 | 0.34 | 0.54 | 0.40 |
| FAT3 | 2.87 | 0.97 | 0.36 | 0.61 | 3.36 | 0.71 | 1.29 | 0.41 | 2.19 | 0.29 |
| FERMT1 | 5.34 | 4.33 | 0.79 | 0.85 | 5.44 | 1.98 | 1.20 | 1.01 | 3.91 | 1.69 |
| FGD4 | 7.89 | 3.75 | 1.17 | 0.60 | 1.76 | 0.89 | 1.57 | 1.19 | 1.46 | 0.75 |
| FHDC1 | 10.55 | 4.13 | 2.36 | 1.49 | 4.26 | 1.79 | 0.94 | 0.74 | 3.28 | 1.47 |
| FKBP5 | 29.76 | 28.70 | 6.55 | 9.86 | 5.81 | 6.65 | 5.54 | 8.17 | 4.59 | 2.40 |
| FOXO1 | 14.66 | 6.32 | 4.33 | 2.03 | 8.40 | 4.26 | 3.49 | 3.95 | 11.23 | 4.37 |
| FST | 112.13 | 119.93 | 18.39 | 15.25 | 32.43 | 34.80 | 24.69 | 17.96 | 29.28 | 46.19 |
| FZD5 | 14.49 | 18.32 | 2.39 | 0.56 | 1.94 | 0.62 | 0.74 | 0.53 | 1.49 | 0.70 |
| GABRA5 | 4.77 | 3.37 | 0.29 | 0.29 | 0.68 | 0.27 | 0.39 | 0.16 | 1.10 | 0.81 |
| GABRB3 | 46.54 | 16.47 | 6.61 | 7.21 | 4.56 | 2.04 | 1.26 | 1.45 | 3.12 | 1.59 |
| GATM | 5.84 | 5.89 | 1.45 | 0.69 | 2.95 | 3.49 | 2.56 | 2.17 | 1.52 | 0.57 |
| GLDC | 33.93 | 31.59 | 8.66 | 9.05 | 8.65 | 5.34 | 2.20 | 3.63 | 7.13 | 5.79 |
| GPC4 | 127.78 | 52.50 | 18.42 | 25.57 | 15.60 | 8.13 | 5.31 | 7.48 | 14.55 | 7.98 |
| GPM6B | 46.73 | 36.30 | 15.37 | 10.26 | 8.11 | 16.42 | 15.15 | 12.94 | 5.18 | 2.71 |
| GPR176 | 23.46 | 12.05 | 3.03 | 2.56 | 1.36 | 0.78 | 1.90 | 1.73 | 1.55 | 0.97 |
| GPR98 | 2.78 | 2.44 | 0.93 | 0.75 | 0.78 | 0.82 | 1.22 | 0.70 | 0.57 | 0.27 |
| GRID2 | 6.17 | 1.73 | 0.55 | 1.06 | 0.52 | 0.16 | 2.81 | 1.28 | 0.32 | 0.04 |
| GRIK5 | 12.31 | 21.32 | 3.91 | 1.66 | 3.33 | 9.14 | 1.63 | 1.67 | 1.40 | 2.12 |
| GRTP1 | 13.08 | 13.96 | 2.68 | 2.44 | 5.08 | 5.38 | 1.95 | 2.28 | 4.27 | 4.17 |
| HDAC9 | 4.50 | 3.36 | 0.75 | 0.39 | 0.60 | 1.10 | 1.08 | 0.38 | 0.51 | 0.14 |
| HOMER2 | 6.44 | 7.06 | 1.77 | 1.52 | 3.06 | 2.00 | 1.31 | 1.35 | 2.45 | 1.50 |
| HOOK1 | 5.07 | 2.95 | 0.56 | 0.49 | 4.36 | 2.25 | 0.90 | 0.63 | 4.46 | 1.16 |
| HSD11B2 | 7.04 | 6.71 | 1.14 | 1.16 | 0.48 | 1.19 | 1.50 | 1.00 | 0.73 | 0.74 |
| HTR3A | 2.22 | 2.77 | 0.14 | 0.04 | 0.05 | 0.18 | 0.61 | 0.04 | 0.07 | 0.12 |
| IQGAP2 | 15.22 | 9.69 | 2.60 | 2.69 | 20.25 | 11.60 | 8.32 | 6.58 | 22.64 | 6.69 |
| IRF2BPL | 19.35 | 14.06 | 4.66 | 4.67 | 8.55 | 11.48 | 13.53 | 13.02 | 7.98 | 8.17 |
| ITGA6 | 53.85 | 32.23 | 7.99 | 6.84 | 11.58 | 6.08 | 14.24 | 9.63 | 11.96 | 3.95 |
| ITGA7 | 11.78 | 16.45 | 3.29 | 1.70 | 3.60 | 6.06 | 1.73 | 1.90 | 1.86 | 2.31 |
| JARID2 | 70.32 | 29.21 | 14.46 | 20.13 | 8.66 | 6.32 | 9.40 | 14.23 | 6.24 | 3.38 |
| KAL1 | 30.58 | 10.06 | 2.98 | 6.85 | 1.36 | 0.63 | 2.12 | 3.52 | 1.86 | 0.52 |
| KIAA1244 | 1.46 | 1.19 | 0.35 | 0.16 | 0.24 | 0.04 | 0.46 | 0.12 | 0.30 | 0.06 |
| KIAA1644 | 3.89 | 1.89 | 0.64 | 0.57 | 0.28 | 0.17 | 1.36 | 1.17 | 0.26 | 0.07 |
| KIF1A | 42.93 | 34.03 | 7.67 | 3.16 | 10.26 | 3.17 | 1.32 | 1.83 | 5.00 | 3.06 |
| KIF21A | 11.04 | 8.68 | 3.31 | 2.82 | 5.98 | 5.05 | 4.92 | 3.37 | 4.57 | 2.87 |
| KLHDC7A | 4.51 | 1.89 | 0.35 | 0.42 | 1.89 | 0.61 | 1.00 | 0.19 | 0.57 | 0.52 |
| L1TD1 | 274.69 | 41.35 | 29.65 | 43.25 | 66.54 | 25.25 | 7.14 | 8.32 | 59.34 | 39.85 |
| LARGE | 17.06 | 19.48 | 5.52 | 7.07 | 8.24 | 4.91 | 6.12 | 6.74 | 7.38 | 4.62 |
| LCK | 14.78 | 5.02 | 1.10 | 1.47 | 0.37 | 0.17 | 0.64 | 0.44 | 0.47 | 0.66 |
| LECT1 | 102.16 | 89.62 | 3.59 | 1.31 | 2.93 | 4.51 | 2.02 | 1.68 | 1.25 | 1.26 |
| LEFTY1 | 28.46 | 1.30 | 0.32 | 0.78 | 0.58 | 0.19 | 0.47 | 0.37 | 1.07 | 1.84 |
| LIMCH1 | 9.20 | 5.70 | 1.85 | 3.06 | 7.76 | 3.50 | 6.04 | 5.48 | 9.03 | 4.24 |

| | | | | | | | | | | |
|---|---|---|---|---|---|---|---|---|---|---|
| LINC-ROR | 5.65 | 0.18 | 0.23 | 0.18 | 0.12 | 0.02 | 0.05 | 0.06 | 0.20 | 0.09 |
| LINC00707 | 9.61 | 2.70 | 0.19 | 0.10 | 0.35 | 0.11 | 0.03 | 0.07 | 0.10 | 0.19 |
| LOC100506013 | 96.62 | 29.47 | 4.07 | 1.30 | 3.65 | 7.15 | 1.25 | 1.01 | 1.51 | 0.99 |
| LRAT | 42.49 | 4.37 | 2.69 | 0.83 | 0.22 | 2.05 | 1.77 | 1.15 | 0.31 | 0.24 |
| LRIG1 | 48.96 | 47.48 | 7.96 | 5.47 | 3.64 | 12.06 | 3.58 | 4.59 | 2.04 | 1.16 |
| LRRC8B | 7.04 | 4.52 | 1.67 | 2.34 | 2.49 | 2.02 | 1.92 | 1.61 | 1.97 | 0.63 |
| MAGI1 | 16.43 | 10.25 | 2.57 | 4.12 | 5.65 | 2.82 | 3.78 | 3.90 | 4.59 | 1.59 |
| MAP7 | 8.16 | 6.54 | 2.19 | 1.98 | 8.89 | 4.53 | 1.01 | 0.88 | 5.79 | 3.28 |
| MEGF10 | 5.96 | 4.37 | 1.20 | 2.20 | 0.31 | 0.26 | 0.98 | 0.91 | 0.88 | 0.20 |
| MRGPRF | 8.76 | 6.81 | 1.23 | 0.83 | 0.45 | 1.77 | 1.46 | 1.07 | 0.37 | 1.00 |
| MTA3 | 66.66 | 71.68 | 23.47 | 32.44 | 33.15 | 37.69 | 28.81 | 29.06 | 25.41 | 24.31 |
| MTM1 | 5.50 | 6.13 | 1.53 | 1.88 | 2.48 | 2.02 | 1.82 | 1.79 | 2.68 | 0.62 |
| MYEF2 | 14.00 | 14.67 | 3.97 | 3.17 | 8.37 | 9.40 | 3.66 | 3.49 | 8.02 | 3.31 |
| MYO5C | 4.27 | 4.19 | 0.85 | 0.81 | 3.19 | 1.21 | 0.77 | 0.57 | 2.29 | 0.72 |
| N4BP3 | 12.50 | 8.68 | 3.73 | 2.80 | 3.15 | 3.16 | 2.22 | 2.42 | 2.16 | 3.10 |
| NANOG | 77.79 | 6.26 | 2.77 | 2.13 | 1.66 | 0.37 | 0.47 | 0.10 | 2.03 | 1.86 |
| NEFL | 5.03 | 7.59 | 1.35 | 0.94 | 0.96 | 2.36 | 0.91 | 0.81 | 0.55 | 0.54 |
| NFASC | 3.73 | 2.07 | 0.50 | 0.14 | 0.76 | 0.51 | 0.93 | 0.36 | 0.59 | 0.26 |
| NFE2L3 | 22.96 | 4.74 | 3.80 | 1.67 | 3.66 | 2.15 | 1.31 | 1.15 | 3.16 | 1.32 |
| NLRP12 | 3.52 | 0.86 | 0.08 | 0.11 | 0.25 | 0.06 | 0.93 | 0.07 | 0.07 | 0.10 |
| NMRK2 | 38.01 | 27.08 | 2.84 | 2.54 | 4.47 | 6.66 | 0.30 | 0.45 | 2.53 | 6.58 |
| NPTX1 | 7.46 | 8.07 | 0.40 | 0.01 | 0.05 | 0.16 | 0.11 | 0.04 | 0.06 | 0.05 |
| NUP210 | 29.92 | 26.31 | 4.42 | 2.60 | 10.47 | 7.21 | 2.18 | 2.96 | 7.94 | 3.22 |
| OAZ2 | 128.93 | 120.80 | 53.44 | 39.34 | 57.15 | 82.83 | 53.40 | 50.66 | 49.56 | 61.75 |
| PAR5 | 19.22 | 1.55 | 1.12 | 5.38 | 2.99 | 0.80 | 1.95 | 3.64 | 3.28 | 0.19 |
| PCDH1 | 62.64 | 36.16 | 6.51 | 1.53 | 12.96 | 11.01 | 2.24 | 1.43 | 9.67 | 7.65 |
| PCDH11X | 3.15 | 0.99 | 0.30 | 0.58 | 1.80 | 1.00 | 0.57 | 0.60 | 1.03 | 0.28 |
| PCNXL2 | 5.74 | 3.99 | 1.62 | 1.80 | 2.82 | 1.00 | 2.25 | 2.16 | 2.43 | 0.83 |
| PDGFA | 19.25 | 9.42 | 6.06 | 3.39 | 7.43 | 9.43 | 3.22 | 3.75 | 5.46 | 9.20 |
| PDGFD | 2.13 | 2.28 | 0.42 | 0.39 | 1.07 | 0.56 | 0.91 | 0.76 | 1.57 | 0.59 |
| PDZD4 | 43.49 | 24.19 | 8.15 | 11.68 | 3.56 | 5.73 | 4.53 | 6.04 | 3.25 | 4.62 |
| PGBD5 | 8.48 | 6.82 | 1.29 | 1.99 | 1.32 | 1.75 | 1.27 | 1.10 | 0.95 | 0.76 |
| PHF15 | 13.89 | 5.51 | 1.59 | 2.96 | 2.17 | 0.72 | 3.39 | 2.59 | 2.30 | 1.43 |
| PIM2 | 54.70 | 24.53 | 6.32 | 8.76 | 3.18 | 4.38 | 2.17 | 2.76 | 3.28 | 4.26 |
| PLA2G3 | 3.60 | 4.21 | 0.64 | 0.66 | 1.15 | 1.89 | 0.83 | 0.58 | 0.93 | 0.85 |
| PLAUR | 8.40 | 7.75 | 1.52 | 1.55 | 2.79 | 3.75 | 1.90 | 1.91 | 2.05 | 3.81 |
| PLCH1 | 7.98 | 6.65 | 1.32 | 1.44 | 0.94 | 1.09 | 1.40 | 0.92 | 0.78 | 0.26 |
| PLS3 | 127.58 | 98.18 | 44.33 | 39.97 | 81.07 | 49.07 | 33.68 | 30.48 | 73.59 | 39.20 |
| PODXL | 373.08 | 261.69 | 55.49 | 75.71 | 188.79 | 119.48 | 208.61 | 193.35 | 181.61 | 58.35 |
| POLR3G | 64.84 | 18.65 | 2.42 | 5.13 | 2.83 | 2.59 | 1.73 | 2.68 | 2.47 | 1.36 |
| POU5F1 | 202.33 | 108.92 | 28.83 | 30.64 | 11.74 | 9.80 | 3.57 | 5.77 | 7.22 | 14.53 |

| | | | | | | | | | |
|---|---|---|---|---|---|---|---|---|---|
| PPM1B | 65.71 | 30.25 | 16.82 | 15.58 | 21.98 | 22.59 | 9.17 | 9.39 | 17.69 | 7.86 |
| PPP1R16B | 3.61 | 0.89 | 0.52 | 0.14 | 0.07 | 0.05 | 1.16 | 1.21 | 0.15 | 0.04 |
| PPP2R2B | 12.95 | 10.66 | 1.62 | 0.35 | 1.95 | 2.89 | 0.79 | 0.29 | 2.47 | 2.37 |
| PPP2R2C | 1.93 | 1.76 | 0.30 | 0.35 | 0.17 | 0.21 | 0.25 | 0.12 | 0.09 | 0.06 |
| PRDM14 | 96.42 | 48.19 | 3.23 | 1.31 | 0.64 | 0.23 | 0.79 | 0.57 | 0.34 | 0.30 |
| PREX2 | 8.50 | 2.63 | 1.36 | 2.15 | 1.82 | 0.55 | 2.03 | 2.20 | 0.96 | 0.08 |
| PRKAA2 | 1.20 | 1.45 | 0.39 | 0.17 | 2.19 | 1.22 | 0.82 | 0.61 | 1.54 | 0.44 |
| PRKCA | 4.40 | 6.22 | 0.98 | 1.48 | 1.84 | 1.41 | 1.07 | 1.27 | 1.57 | 0.40 |
| PRKCQ | 4.96 | 3.08 | 0.80 | 0.66 | 1.44 | 1.30 | 0.81 | 0.88 | 1.64 | 1.52 |
| PRKCQ-AS1 | 4.75 | 3.56 | 0.68 | 0.71 | 1.34 | 2.69 | 0.66 | 1.37 | 1.68 | 1.93 |
| PRRT3 | 5.77 | 7.86 | 1.56 | 0.79 | 2.03 | 3.68 | 1.60 | 1.71 | 1.33 | 1.56 |
| PTPRZ1 | 47.97 | 25.63 | 7.08 | 3.18 | 5.08 | 8.41 | 3.98 | 4.28 | 4.50 | 0.97 |
| PVRL3 | 26.52 | 13.20 | 7.46 | 7.33 | 12.65 | 10.98 | 8.75 | 9.26 | 13.22 | 4.36 |
| RABGAP1L | 28.12 | 10.57 | 2.52 | 3.86 | 5.08 | 0.98 | 5.33 | 4.19 | 3.78 | 1.29 |
| RAP1GAP2 | 5.14 | 3.51 | 1.22 | 0.84 | 3.93 | 0.86 | 0.46 | 0.69 | 3.95 | 1.78 |
| RBM47 | 15.45 | 1.71 | 3.04 | 2.33 | 14.68 | 7.40 | 6.63 | 4.61 | 12.58 | 7.43 |
| RRAGD | 6.18 | 6.03 | 1.20 | 0.65 | 1.56 | 1.33 | 0.82 | 0.85 | 0.93 | 1.11 |
| SALL2 | 83.53 | 69.24 | 22.91 | 13.60 | 15.25 | 19.39 | 13.39 | 15.27 | 10.57 | 5.17 |
| SAMHD1 | 8.10 | 8.86 | 1.85 | 2.13 | 0.87 | 1.01 | 1.72 | 1.71 | 0.97 | 0.25 |
| SBK1 | 18.24 | 29.17 | 6.01 | 4.99 | 10.64 | 10.14 | 3.37 | 5.26 | 7.39 | 6.58 |
| SCAMP5 | 18.04 | 26.41 | 6.37 | 6.51 | 10.01 | 9.09 | 5.68 | 6.06 | 7.89 | 9.01 |
| SCGB3A2 | 64.54 | 58.29 | 10.21 | 3.77 | 10.12 | 9.38 | 1.67 | 1.16 | 4.37 | 10.09 |
| SCNN1A | 14.12 | 11.33 | 2.21 | 0.99 | 4.69 | 2.61 | 1.03 | 0.58 | 2.84 | 2.52 |
| SEMA4D | 8.02 | 12.32 | 2.09 | 2.23 | 5.00 | 5.50 | 2.40 | 2.43 | 3.47 | 2.54 |
| SEPHS1 | 224.88 | 88.51 | 49.08 | 73.88 | 38.59 | 40.27 | 30.61 | 39.28 | 39.72 | 39.15 |
| SERPINE1 | 64.14 | 3.23 | 12.95 | 1.12 | 13.87 | 8.39 | 3.97 | 2.94 | 24.74 | 12.57 |
| SFRP2 | 212.62 | 130.93 | 28.30 | 15.34 | 15.69 | 39.78 | 8.40 | 8.30 | 19.87 | 18.67 |
| SFTA1P | 5.84 | 0.54 | 0.00 | 0.00 | 0.09 | 0.00 | 0.00 | 0.00 | 0.41 | 0.97 |
| SHANK2 | 2.25 | 2.21 | 0.52 | 0.42 | 2.88 | 0.66 | 1.20 | 0.80 | 2.38 | 0.53 |
| SHC3 | 2.32 | 0.38 | 0.28 | 0.15 | 0.17 | 0.15 | 0.28 | 0.26 | 0.09 | 0.07 |
| SHISA3 | 32.52 | 43.82 | 8.60 | 3.07 | 0.24 | 0.64 | 0.97 | 0.52 | 0.36 | 0.18 |
| SLAIN1 | 9.11 | 8.33 | 1.99 | 0.68 | 3.54 | 6.61 | 1.53 | 0.72 | 2.65 | 1.20 |
| SLC12A8 | 4.28 | 4.55 | 0.89 | 0.93 | 1.05 | 0.50 | 0.99 | 0.84 | 0.95 | 1.04 |
| SLC16A9 | 16.18 | 18.87 | 4.29 | 6.01 | 23.18 | 12.19 | 4.80 | 5.28 | 18.31 | 6.71 |
| SLC29A1 | 76.69 | 83.21 | 17.79 | 8.79 | 21.34 | 31.52 | 13.87 | 14.54 | 14.50 | 21.81 |
| SLC38A5 | 25.35 | 13.18 | 6.17 | 5.76 | 0.89 | 2.48 | 13.47 | 10.90 | 1.16 | 1.46 |
| SMIM3 | 43.85 | 41.69 | 2.94 | 1.77 | 4.33 | 5.39 | 4.72 | 4.69 | 2.89 | 3.61 |
| SNTB1 | 4.24 | 3.57 | 0.78 | 0.20 | 1.63 | 0.57 | 0.98 | 0.46 | 1.82 | 0.72 |
| SORBS2 | 7.00 | 3.95 | 0.96 | 0.39 | 0.57 | 0.84 | 1.14 | 0.37 | 1.04 | 0.52 |
| ST8SIA3 | 1.68 | 1.94 | 0.31 | 0.36 | 0.20 | 0.44 | 0.24 | 0.24 | 0.03 | 0.03 |
| STAT3 | 30.92 | 25.12 | 8.06 | 12.19 | 10.01 | 9.87 | 13.50 | 15.07 | 9.71 | 6.14 |
| STK33 | 6.50 | 7.36 | 2.49 | 1.26 | 1.45 | 1.90 | 2.00 | 1.85 | 1.17 | 0.89 |
| SYT13 | 1.61 | 2.78 | 0.43 | 0.49 | 0.78 | 0.40 | 0.54 | 0.50 | 0.51 | 0.25 |

| | | | | | | | | | | |
|---|---|---|---|---|---|---|---|---|---|---|
| SYT6 | 15.86 | 12.85 | 4.81 | 3.59 | 2.48 | 1.30 | 1.48 | 1.63 | 0.97 | 0.48 |
| SYTL2 | 8.27 | 5.28 | 1.71 | 0.18 | 1.12 | 1.50 | 1.33 | 0.42 | 0.88 | 0.45 |
| TCF7L1 | 64.60 | 59.03 | 17.23 | 11.92 | 21.57 | 17.11 | 13.92 | 16.09 | 21.70 | 27.06 |
| TDGF1 | 209.83 | 16.55 | 11.75 | 22.37 | 3.93 | 0.70 | 3.34 | 4.40 | 4.50 | 3.60 |
| TERF1 | 166.58 | 63.38 | 26.83 | 44.25 | 18.69 | 21.64 | 16.37 | 20.86 | 18.38 | 11.91 |
| TFDP2 | 8.15 | 9.84 | 3.67 | 3.26 | 4.22 | 5.07 | 4.33 | 5.01 | 3.80 | 2.38 |
| THBS2 | 6.17 | 3.79 | 0.52 | 0.13 | 0.49 | 0.22 | 0.62 | 0.14 | 0.25 | 0.16 |
| THY1 | 132.29 | 49.69 | 38.62 | 15.85 | 18.41 | 20.23 | 32.58 | 35.40 | 16.23 | 23.02 |
| TLL2 | 1.26 | 1.28 | 0.24 | 0.15 | 0.06 | 0.07 | 0.22 | 0.08 | 0.01 | 0.02 |
| TM4SF18 | 11.21 | 1.08 | 0.21 | 0.10 | 0.04 | 0.19 | 1.95 | 1.58 | 0.14 | 0.03 |
| TM7SF3 | 15.44 | 19.08 | 5.79 | 4.61 | 7.38 | 10.17 | 4.52 | 4.80 | 7.01 | 4.72 |
| TMEM132B | 6.91 | 5.13 | 1.43 | 1.09 | 2.78 | 1.25 | 0.86 | 0.55 | 2.15 | 0.69 |
| TMEM132D | 3.39 | 1.86 | 0.43 | 0.55 | 0.14 | 0.19 | 0.82 | 0.44 | 0.13 | 0.05 |
| TMEM178B | 1.90 | 1.76 | 0.45 | 0.20 | 1.07 | 0.41 | 0.43 | 0.32 | 1.04 | 0.42 |
| TNFRSF8 | 11.80 | 4.30 | 0.15 | 0.31 | 0.24 | 0.10 | 0.30 | 0.14 | 0.32 | 0.48 |
| TOX | 10.98 | 5.95 | 0.47 | 0.31 | 2.85 | 1.37 | 0.55 | 0.45 | 2.84 | 0.97 |
| TOX3 | 7.48 | 10.77 | 2.44 | 0.89 | 1.36 | 1.46 | 1.95 | 1.54 | 1.05 | 0.35 |
| TRERF1 | 6.49 | 6.50 | 1.22 | 1.13 | 3.41 | 1.46 | 3.66 | 3.19 | 3.20 | 1.03 |
| TRIM2 | 19.16 | 15.66 | 7.51 | 5.88 | 5.37 | 5.85 | 5.58 | 5.72 | 4.27 | 1.05 |
| TRIM22 | 8.47 | 9.11 | 0.36 | 0.24 | 0.49 | 0.28 | 0.99 | 0.25 | 0.27 | 0.09 |
| TRIM24 | 104.98 | 94.79 | 27.97 | 51.11 | 46.07 | 25.43 | 34.68 | 40.62 | 55.34 | 27.79 |
| TRIM71 | 95.58 | 32.67 | 14.04 | 31.25 | 21.32 | 3.16 | 10.30 | 20.19 | 15.51 | 1.11 |
| TRNP1 | 12.64 | 11.69 | 4.15 | 2.91 | 3.98 | 6.02 | 4.23 | 4.66 | 3.96 | 6.98 |
| TRPC4 | 14.76 | 16.82 | 2.74 | 1.75 | 1.33 | 1.72 | 2.11 | 1.03 | 0.76 | 0.52 |
| TTC9 | 5.96 | 2.77 | 1.44 | 1.33 | 2.67 | 1.55 | 0.85 | 0.80 | 1.91 | 1.24 |
| UNC13A | 4.20 | 2.57 | 0.31 | 0.95 | 0.25 | 0.08 | 0.26 | 0.15 | 0.26 | 0.06 |
| UNC5D | 7.22 | 3.28 | 0.64 | 0.75 | 0.95 | 0.37 | 0.96 | 0.57 | 0.95 | 0.11 |
| USP44 | 66.82 | 27.30 | 0.95 | 1.04 | 0.73 | 0.95 | 1.20 | 0.57 | 0.60 | 0.22 |
| USP9X | 58.49 | 42.56 | 10.38 | 14.48 | 12.19 | 5.97 | 11.40 | 13.34 | 12.56 | 4.80 |
| VASH2 | 35.51 | 16.66 | 3.30 | 5.94 | 2.43 | 3.45 | 4.14 | 4.75 | 1.90 | 0.50 |
| VAT1L | 91.49 | 109.50 | 12.93 | 6.44 | 13.94 | 9.18 | 14.41 | 10.98 | 6.34 | 2.87 |
| VLDLR | 13.17 | 7.14 | 2.60 | 3.57 | 18.64 | 4.69 | 8.49 | 7.37 | 18.16 | 7.25 |
| VRTN | 93.31 | 69.94 | 12.23 | 36.47 | 4.37 | 6.03 | 4.74 | 8.81 | 2.78 | 3.11 |
| VSNL1 | 15.51 | 5.72 | 4.04 | 0.99 | 1.41 | 0.96 | 1.54 | 0.76 | 0.82 | 0.45 |
| WNK2 | 6.83 | 8.40 | 1.68 | 1.85 | 1.12 | 1.39 | 1.34 | 1.66 | 0.87 | 0.42 |
| WSCD1 | 9.28 | 14.31 | 1.58 | 0.17 | 0.79 | 3.76 | 1.32 | 0.97 | 0.53 | 0.43 |
| ZDHHC22 | 4.50 | 2.44 | 0.70 | 0.66 | 0.25 | 0.47 | 0.91 | 0.55 | 0.24 | 0.22 |
| ZNF589 | 31.23 | 24.70 | 11.56 | 9.76 | 8.82 | 3.87 | 7.07 | 7.96 | 5.77 | 3.52 |
| ZSCAN10 | 61.29 | 32.93 | 8.89 | 7.97 | 5.58 | 4.32 | 2.35 | 3.90 | 2.75 | 5.57 |
| AASS | 34.85 | 26.29 | 12.61 | 16.26 | 11.88 | 9.17 | 10.39 | 12.51 | 12.04 | 4.40 |
| ADAMTS16 | 3.44 | 1.31 | 0.94 | 0.11 | 0.37 | 0.20 | 0.47 | 0.12 | 0.31 | 0.15 |
| ADCY2 | 5.96 | 2.53 | 1.55 | 3.19 | 0.74 | 0.78 | 1.34 | 1.40 | 0.39 | 0.27 |
| AKAP1 | 26.44 | 26.71 | 8.12 | 12.88 | 8.29 | 6.36 | 6.61 | 8.28 | 7.30 | 4.58 |

| | | | | | | | | | |
|---|---|---|---|---|---|---|---|---|---|
| AP1S3 | 2.52 | 1.61 | 0.43 | 0.50 | 0.82 | 1.04 | 0.23 | 0.33 | 1.01 | 0.50 |
| ARC | 5.41 | 2.32 | 1.03 | 0.24 | 0.88 | 1.75 | 0.53 | 0.88 | 1.49 | 2.43 |
| AZIN1 | 123.91 | 74.39 | 51.50 | 63.73 | 71.24 | 66.43 | 34.95 | 39.63 | 65.21 | 26.56 |
| BUB1 | 96.55 | 69.11 | 30.24 | 39.66 | 24.77 | 19.89 | 19.58 | 26.23 | 26.37 | 11.86 |
| CABLES1 | 7.15 | 3.49 | 1.98 | 2.66 | 0.76 | 1.27 | 1.56 | 1.74 | 0.77 | 0.94 |
| CDC42EP3 | 17.00 | 4.32 | 11.41 | 5.95 | 9.06 | 8.82 | 2.74 | 3.78 | 9.89 | 7.91 |
| CDC6 | 69.47 | 44.67 | 23.23 | 31.94 | 26.88 | 34.00 | 12.90 | 16.70 | 24.17 | 19.02 |
| CEBPZ | 121.85 | 61.09 | 35.87 | 43.68 | 24.66 | 25.04 | 19.30 | 21.16 | 22.96 | 15.26 |
| CNTNAP3B | 1.59 | 2.39 | 0.40 | 0.13 | 0.55 | 0.44 | 0.24 | 0.29 | 0.40 | 0.10 |
| CST1 | 113.18 | 14.65 | 39.71 | 27.12 | 6.30 | 18.14 | 12.27 | 13.49 | 16.69 | 52.08 |
| CTSC | 216.98 | 143.86 | 66.19 | 90.90 | 48.99 | 51.92 | 57.28 | 64.41 | 36.34 | 26.04 |
| CUX2 | 1.66 | 2.33 | 0.48 | 0.69 | 0.93 | 0.82 | 0.58 | 0.41 | 0.56 | 0.20 |
| DAB1 | 5.12 | 5.14 | 1.12 | 1.75 | 0.97 | 0.18 | 1.15 | 1.16 | 0.47 | 0.21 |
| DOCK3 | 1.60 | 2.30 | 0.80 | 0.26 | 2.62 | 0.42 | 0.43 | 0.21 | 2.25 | 0.73 |
| ECT2 | 38.61 | 32.00 | 15.70 | 26.09 | 18.26 | 19.82 | 11.66 | 16.28 | 19.24 | 6.94 |
| EMB | 11.81 | 8.72 | 3.99 | 4.55 | 6.64 | 4.88 | 2.52 | 2.62 | 5.28 | 1.88 |
| EPB41L4B | 4.95 | 3.38 | 1.31 | 2.04 | 3.14 | 1.13 | 0.78 | 0.75 | 2.68 | 1.67 |
| ESRP1 | 15.58 | 5.93 | 7.24 | 5.79 | 20.85 | 8.42 | 2.59 | 1.92 | 17.13 | 8.66 |
| FAM111B | 14.34 | 9.74 | 4.10 | 5.27 | 8.52 | 5.87 | 3.99 | 3.04 | 8.91 | 3.63 |
| FAM189A2 | 8.55 | 1.38 | 1.82 | 0.69 | 1.09 | 0.28 | 0.69 | 0.32 | 1.12 | 1.17 |
| FANCD2 | 28.36 | 26.98 | 12.72 | 18.44 | 12.88 | 13.09 | 7.42 | 12.05 | 11.36 | 7.83 |
| FAT1 | 27.49 | 16.08 | 11.93 | 8.48 | 16.94 | 4.02 | 5.43 | 7.92 | 15.83 | 2.44 |
| FGF2 | 16.14 | 8.81 | 3.69 | 5.84 | 4.30 | 2.81 | 3.53 | 4.71 | 2.50 | 1.21 |
| GAL | 131.65 | 28.43 | 51.84 | 59.87 | 8.67 | 5.34 | 7.62 | 16.29 | 10.76 | 22.62 |
| GDAP1 | 11.32 | 8.63 | 4.78 | 6.73 | 6.06 | 4.65 | 2.84 | 2.55 | 5.65 | 2.75 |
| GPRC5B | 61.39 | 37.93 | 18.26 | 22.32 | 6.17 | 7.39 | 8.51 | 12.30 | 3.94 | 2.82 |
| GSPT2 | 20.74 | 22.78 | 11.68 | 10.66 | 10.58 | 12.80 | 6.23 | 6.88 | 8.42 | 5.81 |
| HELLS | 36.03 | 20.96 | 13.52 | 17.27 | 13.39 | 19.11 | 7.35 | 9.57 | 10.98 | 6.45 |
| HHLA1 | 5.00 | 0.23 | 0.56 | 0.91 | 0.47 | 0.11 | 0.40 | 0.32 | 0.70 | 0.43 |
| ICMT | 52.32 | 53.82 | 27.37 | 33.51 | 31.12 | 35.40 | 17.11 | 23.88 | 28.35 | 22.74 |
| IDO1 | 13.13 | 7.01 | 0.28 | 0.10 | 0.17 | 0.14 | 0.71 | 0.11 | 0.25 | 1.06 |
| IL17RD | 20.57 | 2.41 | 4.49 | 10.80 | 4.16 | 6.05 | 3.41 | 4.46 | 3.61 | 1.51 |
| IPW | 30.57 | 20.36 | 13.90 | 11.38 | 12.07 | 9.63 | 4.27 | 5.95 | 9.22 | 5.89 |
| ITPR3 | 9.08 | 6.15 | 2.79 | 3.96 | 6.31 | 1.09 | 2.46 | 3.20 | 7.10 | 2.31 |
| KIAA1804 | 3.29 | 2.79 | 0.79 | 1.20 | 0.63 | 0.55 | 1.04 | 0.76 | 0.63 | 0.19 |
| KLKB1 | 7.05 | 2.17 | 1.30 | 2.21 | 0.41 | 0.29 | 1.06 | 0.58 | 0.27 | 0.21 |
| LOC653653 | 14.50 | 8.73 | 3.15 | 3.92 | 3.88 | 2.03 | 3.03 | 2.44 | 2.53 | 0.22 |
| LRRN1 | 91.80 | 65.01 | 41.53 | 32.67 | 142.75 | 52.66 | 16.86 | 22.56 | 107.86 | 30.81 |
| MAL2 | 9.92 | 7.99 | 4.48 | 1.68 | 9.19 | 5.71 | 1.55 | 1.47 | 6.65 | 3.92 |
| MCM3 | 144.93 | 156.77 | 83.62 | 83.11 | 82.65 | 86.48 | 47.19 | 66.21 | 72.15 | 66.08 |
| MCM4 | 120.75 | 107.01 | 69.84 | 80.09 | 62.38 | 73.78 | 36.72 | 51.18 | 55.67 | 38.88 |
| MGC45800 | 4.58 | 2.96 | 2.67 | 1.32 | 5.30 | 3.08 | 1.01 | 1.17 | 5.71 | 3.39 |

| | | | | | | | | | | |
|---|---|---|---|---|---|---|---|---|---|---|
| MLF1IP | 58.38 | 46.83 | 25.45 | 32.49 | 25.89 | 29.19 | 16.52 | 20.39 | 24.93 | 19.95 |
| MOCOS | 5.96 | 4.33 | 1.36 | 2.18 | 1.87 | 1.11 | 1.20 | 1.23 | 1.49 | 1.46 |
| MSH2 | 115.36 | 63.37 | 34.65 | 51.38 | 44.45 | 36.38 | 27.30 | 30.95 | 40.25 | 17.61 |
| MTHFD1 | 80.31 | 49.31 | 40.04 | 59.67 | 34.58 | 31.66 | 19.65 | 29.88 | 32.28 | 22.18 |
| MYO10 | 26.84 | 22.89 | 16.06 | 18.60 | 14.99 | 6.53 | 8.31 | 11.22 | 13.94 | 6.22 |
| MYO1E | 10.36 | 8.24 | 3.98 | 3.63 | 5.27 | 1.78 | 2.65 | 2.72 | 4.19 | 1.76 |
| NAA50 | 51.26 | 35.73 | 25.76 | 38.28 | 26.55 | 33.07 | 15.67 | 19.87 | 23.33 | 13.59 |
| NLN | 26.15 | 15.98 | 7.95 | 15.50 | 10.62 | 7.42 | 5.44 | 7.66 | 9.29 | 4.83 |
| NLRP2 | 39.72 | 31.20 | 15.19 | 18.32 | 27.80 | 21.04 | 10.84 | 12.51 | 23.71 | 19.74 |
| NQO2 | 47.13 | 34.34 | 13.44 | 19.78 | 9.37 | 13.99 | 10.70 | 11.42 | 7.59 | 12.52 |
| NR6A1 | 45.05 | 32.10 | 9.50 | 22.03 | 12.54 | 2.94 | 10.22 | 17.17 | 6.12 | 1.21 |
| ORC1 | 40.19 | 31.17 | 15.71 | 17.93 | 13.18 | 10.23 | 7.48 | 10.49 | 12.20 | 9.38 |
| PAK1 | 59.96 | 33.02 | 20.15 | 26.61 | 21.92 | 18.21 | 15.50 | 17.86 | 19.69 | 15.60 |
| PARP1 | 186.95 | 176.62 | 84.24 | 77.31 | 89.92 | 105.1<br>4 | 57.25 | 70.68 | 76.11 | 54.18 |
| PAWR | 26.63 | 15.59 | 7.29 | 10.44 | 29.22 | 18.20 | 5.44 | 8.77 | 24.31 | 11.65 |
| PCSK9 | 10.54 | 3.38 | 2.44 | 3.40 | 0.94 | 0.22 | 1.49 | 2.56 | 0.80 | 0.79 |
| PCYT1B | 5.22 | 7.09 | 2.15 | 2.23 | 3.17 | 2.69 | 1.67 | 2.51 | 1.80 | 1.20 |
| PKI55 | 7.03 | 4.79 | 4.80 | 2.94 | 3.68 | 4.04 | 1.39 | 1.80 | 4.67 | 4.07 |
| PLCB3 | 24.97 | 23.36 | 7.38 | 12.81 | 10.30 | 10.83 | 7.98 | 11.09 | 9.24 | 9.88 |
| PLS1 | 5.93 | 5.87 | 1.44 | 3.00 | 3.70 | 3.34 | 1.47 | 1.80 | 2.48 | 0.90 |
| PMAIP1 | 47.57 | 12.61 | 21.77 | 23.14 | 7.61 | 2.61 | 4.48 | 10.04 | 7.24 | 2.87 |
| PPP1R3B | 24.21 | 11.12 | 9.45 | 15.78 | 7.94 | 5.51 | 6.35 | 6.83 | 6.24 | 2.75 |
| PSAT1 | 224.43 | 175.31 | 129.9<br>2 | 126.5<br>4 | 170.7<br>6 | 76.27 | 41.84 | 73.34 | 177.7<br>1 | 116.31 |
| PSIP1 | 240.90 | 186.49 | 85.51 | 80.78 | 77.05 | 93.51 | 75.10 | 76.14 | 62.60 | 46.75 |
| RAB39B | 3.93 | 4.37 | 1.70 | 2.46 | 1.89 | 1.04 | 0.90 | 0.97 | 1.11 | 0.55 |
| RFWD3 | 41.15 | 26.36 | 20.22 | 28.29 | 19.91 | 14.78 | 10.49 | 14.83 | 17.49 | 7.83 |
| RNF138 | 35.36 | 24.34 | 15.32 | 20.55 | 14.70 | 22.21 | 10.24 | 12.94 | 14.71 | 7.50 |
| ROR1 | 34.90 | 25.69 | 14.41 | 24.23 | 15.19 | 8.75 | 10.21 | 12.08 | 17.66 | 5.62 |
| RTN3 | 93.59 | 121.11 | 52.25 | 32.47 | 52.13 | 77.34 | 29.06 | 29.85 | 44.92 | 43.16 |
| RTN4IP1 | 10.57 | 10.01 | 2.53 | 4.72 | 3.10 | 3.79 | 2.95 | 2.81 | 2.91 | 3.48 |
| SCG3 | 14.93 | 6.36 | 4.11 | 4.89 | 6.25 | 2.99 | 1.28 | 1.55 | 4.71 | 3.24 |
| SELRC1 | 32.01 | 20.00 | 12.47 | 21.69 | 10.75 | 7.14 | 8.23 | 9.77 | 9.58 | 5.30 |
| SEMA6A | 111.47 | 45.04 | 42.13 | 41.20 | 53.08 | 16.08 | 14.69 | 18.24 | 34.94 | 14.74 |
| SHISA9 | 11.05 | 4.92 | 4.55 | 4.33 | 5.81 | 3.12 | 2.56 | 2.76 | 5.89 | 4.39 |
| SLC16A1 | 204.29 | 162.83 | 89.68 | 144.5<br>9 | 104.4<br>4 | 88.99 | 54.61 | 73.81 | 73.06 | 40.23 |
| SLC7A1 | 33.51 | 14.88 | 18.50 | 14.27 | 25.21 | 7.84 | 5.09 | 8.76 | 24.72 | 8.08 |
| SLC7A11 | 11.26 | 3.84 | 7.02 | 3.15 | 11.85 | 2.98 | 1.53 | 2.14 | 11.52 | 4.54 |
| SLC7A3 | 41.84 | 39.82 | 20.85 | 13.56 | 23.47 | 17.34 | 6.96 | 11.34 | 24.11 | 20.52 |
| STEAP3 | 8.09 | 6.29 | 2.76 | 4.25 | 3.10 | 1.53 | 1.45 | 1.45 | 1.96 | 1.99 |
| TAF4B | 11.07 | 3.11 | 3.56 | 3.73 | 1.43 | 0.70 | 1.95 | 2.42 | 1.22 | 0.38 |

| | | | | | | | | | | |
|---|---|---|---|---|---|---|---|---|---|---|
| TJP2 | 24.69 | 9.18 | 8.20 | 5.43 | 30.20 | 8.82 | 5.15 | 4.75 | 34.16 | 19.13 |
| TMEM178A | 7.10 | 4.86 | 1.12 | 0.27 | 2.12 | 1.89 | 1.20 | 0.54 | 1.73 | 1.89 |
| TUBB2A | 568.21 | 293.19 | 233.55 | 187.28 | 196.57 | 193.07 | 60.56 | 95.15 | 169.78 | 300.55 |
| TXLNG | 24.16 | 12.16 | 10.11 | 13.60 | 10.04 | 6.86 | 6.67 | 7.43 | 9.42 | 4.06 |
| TXNRD1 | 63.03 | 42.39 | 29.88 | 38.92 | 39.26 | 32.91 | 19.09 | 21.36 | 42.32 | 22.31 |
| WWC1 | 4.74 | 6.63 | 3.20 | 1.65 | 7.71 | 3.09 | 1.54 | 1.59 | 7.54 | 4.92 |
| YEATS4 | 42.47 | 48.56 | 15.54 | 26.15 | 28.22 | 42.46 | 15.35 | 18.77 | 24.30 | 18.06 |

Figure 2E. Cluster II genes

| Genes | hESC | | Day 3 MESP1$^+$ | | Day 3 MESP1$^-$ | | Day 5 MESP1$^+$ | | Day 5 MESP1$^-$ | |
|---|---|---|---|---|---|---|---|---|---|---|
| | R1 | R2 | R1 | R2 | R1 | R2 | R1 | R2 | R1 | R2 |
| ACE | 0.34 | 0.23 | 1.32 | 3.15 | 0.36 | 0.30 | 2.06 | 2.40 | 0.22 | 0.30 |
| ACSL6 | 0.05 | 0.04 | 0.25 | 0.80 | 0.02 | 0.02 | 0.25 | 0.21 | 0.01 | 0.00 |
| ADRA2C | 3.37 | 7.97 | 15.01 | 20.64 | 4.65 | 8.73 | 5.15 | 10.70 | 3.65 | 10.12 |
| ALDH2 | 28.89 | 28.70 | 71.21 | 106.09 | 35.24 | 32.88 | 62.40 | 83.76 | 44.69 | 52.13 |
| ALPK2 | 0.40 | 0.10 | 10.19 | 22.68 | 7.46 | 1.29 | 8.95 | 14.49 | 11.96 | 4.09 |
| ALPK3 | 5.12 | 2.59 | 10.62 | 19.09 | 9.19 | 1.49 | 7.25 | 11.39 | 6.68 | 1.93 |
| AMER3 | 0.03 | 0.09 | 0.20 | 3.06 | 0.34 | 0.67 | 0.67 | 0.49 | 0.10 | 0.05 |
| AMIGO2 | 2.56 | 1.40 | 35.79 | 58.39 | 2.87 | 6.68 | 41.22 | 45.46 | 2.64 | 1.53 |
| ANKRD55 | 0.00 | 0.00 | 0.16 | 1.17 | 0.00 | 0.09 | 0.61 | 0.45 | 0.04 | 0.00 |
| APLNR | 2.14 | 1.01 | 184.27 | 385.56 | 5.69 | 14.26 | 81.18 | 156.43 | 3.28 | 3.78 |
| APOBEC3B | 5.37 | 5.04 | 12.00 | 21.79 | 7.71 | 8.02 | 7.01 | 8.99 | 5.95 | 7.11 |
| APOBEC3G | 0.59 | 0.84 | 3.66 | 11.96 | 0.89 | 1.09 | 4.06 | 5.85 | 1.23 | 0.85 |
| ATP12A | 0.12 | 0.10 | 6.99 | 18.46 | 3.83 | 0.25 | 2.16 | 3.29 | 1.07 | 0.33 |
| ATP8B3 | 0.60 | 1.48 | 2.44 | 4.58 | 0.58 | 0.30 | 2.30 | 2.38 | 0.38 | 0.25 |
| BAALC | 0.75 | 1.94 | 4.74 | 6.65 | 1.04 | 2.59 | 4.21 | 5.87 | 0.24 | 0.23 |
| BCAR3 | 4.43 | 3.44 | 19.78 | 26.02 | 8.37 | 8.47 | 13.73 | 17.07 | 8.49 | 6.16 |
| BMP2 | 6.60 | 5.38 | 31.60 | 24.72 | 1.97 | 3.02 | 11.54 | 19.44 | 1.41 | 0.96 |
| BMPER | 0.36 | 0.11 | 14.42 | 34.40 | 3.32 | 3.29 | 5.20 | 11.10 | 2.48 | 1.18 |
| C8orf31 | 0.21 | 0.19 | 3.39 | 6.66 | 1.23 | 0.58 | 3.70 | 5.07 | 0.84 | 0.71 |
| CA12 | 1.05 | 1.84 | 4.39 | 13.29 | 6.63 | 1.99 | 6.27 | 7.78 | 3.30 | 2.28 |
| CA2 | 5.85 | 5.88 | 51.20 | 45.40 | 14.27 | 17.54 | 6.02 | 12.65 | 11.18 | 10.79 |
| CALCB | 0.92 | 1.96 | 25.01 | 17.49 | 3.07 | 2.60 | 2.28 | 4.47 | 4.57 | 5.57 |
| CD1D | 0.34 | 0.36 | 3.61 | 8.57 | 0.38 | 0.54 | 1.88 | 2.72 | 0.35 | 0.21 |
| CER1 | 15.08 | 0.62 | 28.86 | 55.54 | 2.97 | 0.54 | 6.61 | 12.42 | 2.26 | 0.86 |
| CGNL1 | 7.79 | 3.87 | 21.17 | 32.44 | 18.84 | 6.86 | 17.41 | 27.91 | 15.27 | 6.82 |
| CLSTN2 | 0.41 | 1.10 | 3.17 | 9.74 | 8.23 | 2.64 | 3.50 | 4.03 | 3.17 | 1.23 |
| COL13A1 | 2.47 | 9.21 | 13.17 | 26.91 | 2.29 | 5.83 | 15.43 | 21.60 | 1.32 | 1.52 |
| COL9A2 | 2.33 | 5.86 | 35.95 | 54.97 | 7.25 | 12.95 | 12.73 | 25.96 | 4.43 | 7.42 |
| CRLF1 | 0.89 | 1.32 | 4.17 | 8.19 | 0.50 | 0.71 | 1.19 | 2.24 | 0.45 | 0.71 |
| CYP27C1 | 1.52 | 2.39 | 6.49 | 5.05 | 2.62 | 2.35 | 2.34 | 3.29 | 1.63 | 1.24 |
| DENND2C | 0.73 | 0.58 | 1.79 | 3.71 | 0.36 | 0.08 | 1.34 | 1.13 | 0.34 | 0.10 |

| | | | | | | | | | |
|---|---|---|---|---|---|---|---|---|---|
| DLL3 | 1.07 | 3.04 | 26.31 | 76.37 | 0.26 | 6.23 | 11.26 | 21.76 | 0.47 | 1.13 |
| DUSP10 | 2.01 | 1.25 | 7.50 | 5.58 | 1.33 | 1.95 | 2.73 | 4.38 | 3.11 | 2.88 |
| EGF | 0.80 | 0.38 | 1.84 | 2.99 | 0.43 | 0.36 | 0.90 | 1.01 | 0.20 | 0.12 |
| ELN | 1.25 | 2.34 | 4.45 | 9.48 | 0.82 | 3.11 | 2.66 | 6.32 | 0.24 | 0.55 |
| EOMES | 8.96 | 0.12 | 10.82 | 27.55 | 1.30 | 0.28 | 2.56 | 4.07 | 2.31 | 1.29 |
| FAM222A | 1.97 | 2.02 | 6.09 | 6.36 | 0.88 | 4.00 | 2.33 | 4.00 | 1.05 | 1.07 |
| FBN2 | 12.14 | 8.44 | 34.52 | 86.51 | 31.00 | 9.22 | 27.73 | 44.55 | 21.81 | 3.30 |
| FGF19 | 3.08 | 1.22 | 8.52 | 36.37 | 0.97 | 5.69 | 7.86 | 22.40 | 0.40 | 0.45 |
| FGFRL1 | 4.70 | 4.51 | 9.03 | 20.01 | 4.07 | 6.53 | 10.46 | 15.74 | 6.69 | 3.86 |
| FNIP2 | 2.75 | 1.79 | 10.72 | 9.29 | 5.40 | 1.08 | 6.66 | 7.19 | 4.50 | 0.53 |
| GATA4 | 1.21 | 0.11 | 17.47 | 29.17 | 2.81 | 6.99 | 9.42 | 14.17 | 3.11 | 2.53 |
| GATA6 | 1.47 | 0.18 | 16.98 | 53.61 | 5.84 | 18.03 | 45.23 | 56.04 | 6.72 | 4.35 |
| GYPB | 0.00 | 0.00 | 0.44 | 1.21 | 0.00 | 0.00 | 0.00 | 0.00 | 0.00 | 0.00 |
| GYPE | 0.00 | 0.00 | 0.67 | 1.25 | 0.03 | 0.00 | 0.36 | 0.12 | 0.00 | 0.00 |
| HAS2 | 11.97 | 1.81 | 84.49 | 155.97 | 13.15 | 13.28 | 35.82 | 61.11 | 14.80 | 5.11 |
| HHIPL2 | 0.08 | 0.33 | 4.17 | 7.03 | 2.88 | 3.21 | 4.57 | 5.43 | 1.22 | 1.14 |
| HLA-DQB1 | 4.54 | 2.56 | 20.10 | 34.52 | 6.19 | 10.30 | 6.58 | 10.14 | 3.98 | 7.71 |
| HOXD13 | 0.00 | 0.00 | 1.73 | 4.25 | 0.04 | 0.61 | 2.59 | 2.25 | 0.08 | 0.10 |
| HS3ST3A1 | 1.97 | 3.32 | 9.31 | 21.71 | 1.48 | 3.06 | 3.99 | 8.74 | 1.05 | 1.72 |
| IKZF1 | 0.09 | 0.17 | 0.45 | 1.20 | 0.32 | 0.04 | 0.28 | 0.27 | 0.60 | 0.31 |
| IL13RA1 | 6.77 | 2.90 | 10.53 | 18.06 | 6.46 | 4.03 | 10.14 | 9.64 | 7.03 | 4.33 |
| ITGB8 | 0.49 | 0.94 | 2.93 | 2.93 | 0.24 | 0.52 | 1.37 | 1.85 | 0.27 | 0.10 |
| JAKMIP1 | 0.40 | 1.09 | 1.01 | 9.48 | 0.26 | 0.07 | 1.46 | 2.06 | 0.29 | 0.23 |
| JHDM1D | 1.58 | 1.24 | 1.95 | 7.89 | 3.52 | 1.59 | 3.26 | 4.30 | 2.74 | 0.74 |
| KEL | 1.75 | 0.44 | 6.47 | 4.58 | 0.70 | 0.44 | 2.62 | 2.42 | 0.87 | 1.16 |
| KIAA1024 | 0.06 | 0.12 | 1.80 | 5.60 | 0.44 | 0.11 | 2.00 | 2.17 | 0.58 | 0.16 |
| KIAA1551 | 21.63 | 7.93 | 53.04 | 83.00 | 59.02 | 19.43 | 40.98 | 51.66 | 39.14 | 9.25 |
| LGR5 | 0.48 | 0.04 | 9.80 | 14.75 | 1.13 | 0.24 | 3.13 | 5.41 | 0.48 | 0.06 |
| LHX1 | 0.89 | 0.34 | 6.47 | 7.03 | 0.18 | 0.43 | 1.40 | 3.94 | 0.11 | 0.12 |
| LINC00261 | 1.41 | 0.24 | 5.02 | 2.98 | 0.47 | 1.52 | 1.19 | 1.05 | 0.56 | 0.35 |
| LPGAT1 | 3.29 | 2.35 | 6.93 | 10.09 | 5.27 | 2.50 | 5.43 | 5.89 | 6.21 | 2.15 |
| LRRC37BP1 | 6.10 | 6.39 | 13.46 | 19.66 | 13.84 | 11.29 | 7.51 | 8.84 | 8.18 | 5.57 |
| LZTS1 | 8.55 | 1.43 | 11.68 | 35.90 | 0.61 | 0.76 | 5.51 | 9.17 | 0.84 | 0.56 |
| MALT1 | 5.89 | 6.26 | 12.89 | 24.58 | 10.20 | 5.93 | 9.07 | 11.29 | 10.26 | 4.63 |
| MESP2 | 0.25 | 0.36 | 4.77 | 9.99 | 0.03 | 0.08 | 1.69 | 3.36 | 0.12 | 0.12 |
| MIXL1 | 2.43 | 0.23 | 6.38 | 50.10 | 1.47 | 1.95 | 5.31 | 10.97 | 2.62 | 1.60 |
| MSGN1 | 0.00 | 0.00 | 8.46 | 43.72 | 0.00 | 0.57 | 9.59 | 16.73 | 0.00 | 0.00 |
| MYC | 20.43 | 4.92 | 55.25 | 34.89 | 16.45 | 12.12 | 24.15 | 34.18 | 22.01 | 21.76 |
| MYLK3 | 0.10 | 0.05 | 1.45 | 2.79 | 2.23 | 1.36 | 1.53 | 1.86 | 1.10 | 0.63 |
| NAV2 | 20.25 | 10.37 | 23.95 | 71.28 | 26.51 | 8.03 | 28.42 | 46.46 | 24.41 | 5.90 |
| NRG2 | 0.09 | 0.24 | 3.62 | 3.98 | 0.14 | 0.37 | 1.62 | 2.66 | 0.10 | 0.16 |
| ODAM | 0.00 | 0.00 | 3.57 | 4.69 | 0.86 | 0.70 | 2.74 | 3.92 | 0.73 | 0.35 |
| PCED1B | 2.00 | 0.69 | 6.68 | 9.38 | 0.38 | 0.66 | 4.00 | 5.23 | 0.54 | 0.58 |

| | | | | | | | | | | |
|---|---|---|---|---|---|---|---|---|---|---|
| PCSK2 | 0.10 | 1.63 | 4.59 | 8.74 | 0.51 | 0.55 | 1.09 | 1.19 | 0.36 | 0.42 |
| PELI2 | 2.98 | 4.37 | 8.63 | 10.09 | 1.43 | 2.16 | 4.60 | 6.78 | 1.30 | 0.58 |
| PENK | 0.19 | 0.38 | 6.50 | 12.61 | 0.37 | 2.49 | 7.10 | 6.83 | 0.13 | 0.43 |
| PLEKHA6 | 1.69 | 1.72 | 5.30 | 9.90 | 5.11 | 1.95 | 3.32 | 5.22 | 3.73 | 2.80 |
| PLXNA2 | 0.69 | 1.12 | 15.49 | 33.59 | 11.07 | 4.28 | 15.91 | 22.32 | 15.41 | 4.71 |
| PRDM1 | 0.66 | 0.27 | 3.48 | 3.68 | 0.91 | 0.79 | 1.68 | 0.82 | 0.41 | 0.35 |
| PVR | 6.17 | 4.70 | 19.19 | 16.72 | 9.79 | 6.51 | 11.83 | 13.06 | 9.33 | 7.72 |
| RASGRP3 | 0.10 | 0.03 | 2.06 | 7.73 | 0.09 | 0.08 | 3.50 | 4.70 | 0.08 | 0.01 |
| RGS13 | 0.00 | 0.00 | 8.45 | 23.14 | 0.11 | 0.56 | 4.99 | 6.90 | 0.19 | 0.12 |
| RGS2 | 11.06 | 14.59 | 51.02 | 43.24 | 4.63 | 10.90 | 25.25 | 37.97 | 5.61 | 4.65 |
| SEMA6D | 6.91 | 3.22 | 16.84 | 29.27 | 3.74 | 3.76 | 21.12 | 21.07 | 3.59 | 0.86 |
| SERPINB9 | 13.20 | 1.79 | 62.49 | 104.14 | 24.83 | 11.69 | 40.54 | 44.55 | 15.85 | 8.16 |
| SERPINE2 | 122.55 | 43.96 | 212.02 | 337.10 | 37.67 | 36.62 | 55.71 | 96.78 | 68.52 | 58.64 |
| SEZ6L | 0.16 | 0.28 | 1.08 | 2.73 | 0.92 | 0.59 | 1.29 | 1.38 | 0.45 | 0.13 |
| SHISA6 | 1.04 | 1.37 | 1.84 | 8.37 | 0.40 | 0.24 | 1.62 | 3.34 | 0.29 | 0.09 |
| SLC39A8 | 8.22 | 3.40 | 14.62 | 18.24 | 13.00 | 10.99 | 15.43 | 12.82 | 9.27 | 5.92 |
| SLPI | 0.00 | 0.00 | 1.54 | 1.43 | 0.11 | 0.27 | 0.54 | 0.28 | 0.00 | 0.13 |
| SMAD9 | 0.15 | 0.34 | 3.78 | 4.57 | 2.81 | 2.76 | 3.00 | 3.04 | 2.74 | 0.77 |
| SNAI1 | 3.98 | 5.25 | 19.43 | 49.39 | 6.30 | 8.14 | 20.45 | 57.65 | 7.57 | 9.32 |
| SOX17 | 1.30 | 0.35 | 24.78 | 16.72 | 1.40 | 2.79 | 3.69 | 4.59 | 1.51 | 3.21 |
| ST3GAL6 | 1.24 | 2.77 | 6.52 | 10.24 | 3.25 | 4.95 | 6.09 | 8.85 | 3.58 | 2.62 |
| SYNJ1 | 1.00 | 0.56 | 2.55 | 4.37 | 2.20 | 1.19 | 2.74 | 2.78 | 1.78 | 0.39 |
| SYNM | 0.74 | 0.74 | 3.20 | 2.37 | 1.59 | 0.78 | 1.57 | 1.05 | 1.34 | 0.62 |
| T | 0.78 | 0.21 | 19.85 | 29.72 | 1.55 | 1.25 | 5.98 | 10.22 | 1.19 | 0.84 |
| TBX6 | 0.36 | 1.17 | 8.98 | 16.93 | 0.70 | 0.81 | 4.80 | 10.03 | 0.48 | 0.78 |
| TERF2IP | 8.09 | 10.60 | 19.87 | 42.25 | 13.40 | 21.52 | 28.63 | 34.89 | 13.64 | 15.56 |
| TMEM185A | 6.88 | 8.32 | 34.29 | 51.09 | 7.18 | 8.05 | 23.85 | 34.10 | 6.93 | 6.94 |
| TP53INP1 | 1.62 | 3.28 | 6.16 | 9.43 | 7.65 | 5.68 | 7.29 | 7.58 | 7.05 | 2.89 |
| TSLP | 0.23 | 0.20 | 2.07 | 2.59 | 0.34 | 0.46 | 1.36 | 1.35 | 0.23 | 0.02 |
| WNT3 | 3.16 | 1.36 | 5.89 | 14.54 | 5.93 | 2.92 | 5.19 | 5.66 | 5.15 | 2.34 |
| WNT5A | 1.56 | 2.46 | 29.28 | 90.82 | 12.25 | 10.01 | 29.46 | 54.23 | 17.23 | 6.95 |
| WNT8A | 0.65 | 7.95 | 20.14 | 35.16 | 1.37 | 1.79 | 12.54 | 18.18 | 0.59 | 0.04 |
| ZNF516 | 3.25 | 5.71 | 7.28 | 16.20 | 4.99 | 4.59 | 8.78 | 14.36 | 4.24 | 2.01 |
| ZNF611 | 3.52 | 1.87 | 12.65 | 10.65 | 4.81 | 2.10 | 5.89 | 6.72 | 4.62 | 1.38 |
| ANXA6 | 57.80 | 56.37 | 96.70 | 145.01 | 60.23 | 92.30 | 124.40 | 140.42 | 56.56 | 68.86 |
| IGFBP4 | 39.78 | 39.15 | 68.91 | 115.27 | 13.69 | 50.99 | 85.51 | 111.63 | 15.93 | 38.58 |
| ABTB2 | 6.45 | 1.94 | 10.32 | 10.61 | 2.29 | 2.66 | 2.87 | 4.47 | 1.99 | 1.46 |
| ALDH1A3 | 3.32 | 0.84 | 3.63 | 10.17 | 0.30 | 0.05 | 0.92 | 2.03 | 0.58 | 0.21 |
| CALB1 | 3.63 | 1.33 | 5.17 | 3.79 | 0.67 | 0.23 | 0.47 | 0.33 | 0.66 | 0.19 |
| CHCHD2 | 0.31 | 0.00 | 0.59 | 0.69 | 0.15 | 0.00 | 0.00 | 0.00 | 0.00 | 0.00 |
| FABP5 | 320.75 | 174.97 | 365.73 | 559.21 | 95.07 | 258.41 | 159.07 | 214.01 | 90.35 | 139.23 |
| HSPH1 | 55.13 | 34.56 | 56.96 | 74.81 | 38.45 | 38.43 | 18.07 | 29.62 | 34.81 | 20.34 |
| LEPREL1 | 20.19 | 19.13 | 22.14 | 40.07 | 20.70 | 13.81 | 8.08 | 14.97 | 16.65 | 10.95 |

| | | | | | | | | | | |
|---|---|---|---|---|---|---|---|---|---|---|
| NTS | 152.48 | 4.31 | 151.64 | 307.42 | 12.26 | 7.28 | 29.97 | 46.88 | 6.27 | 4.68 |

Figure 2E. Cluster III genes

| Genes | hESC | | Day 3 MESP1$^{+}$ | | Day 3 MESP1$^{-}$ | | Day 5 MESP1$^{+}$ | | Day 5 MESP1$^{-}$ | |
|---|---|---|---|---|---|---|---|---|---|---|
| | R1 | R2 | R1 | R2 | R1 | R2 | R1 | R2 | R1 | R2 |
| COL3A1 | 0.84 | 1.40 | 224.29 | 151.47 | 345.37 | 292.56 | 5035.29 | 3197.53 | 686.05 | 301.08 |
| COL1A1 | 36.82 | 20.91 | 113.15 | 110.68 | 184.26 | 278.01 | 1506.58 | 1289.41 | 298.52 | 248.63 |
| SPARC | 156.34 | 103.46 | 262.77 | 282.76 | 262.71 | 211.91 | 1275.25 | 847.71 | 377.40 | 337.25 |
| IGF2 | 0.50 | 3.08 | 159.84 | 101.79 | 73.38 | 262.40 | 1265.50 | 1056.50 | 194.96 | 277.47 |
| COL6A2 | 64.31 | 28.08 | 190.38 | 218.69 | 41.38 | 75.77 | 1109.28 | 986.89 | 64.78 | 106.72 |
| POSTN | 1.99 | 0.35 | 9.47 | 5.81 | 3.45 | 10.13 | 965.83 | 535.86 | 15.13 | 6.27 |
| COL6A1 | 56.98 | 25.72 | 279.97 | 405.71 | 45.87 | 58.49 | 848.43 | 841.83 | 57.14 | 50.17 |
| COL1A2 | 17.33 | 10.55 | 21.56 | 26.16 | 72.22 | 113.17 | 537.01 | 394.29 | 138.65 | 81.49 |
| COL5A1 | 11.42 | 8.73 | 71.65 | 103.96 | 92.02 | 38.66 | 379.67 | 334.28 | 142.49 | 82.35 |
| FOS | 74.01 | 68.77 | 17.61 | 10.67 | 110.34 | 192.27 | 370.42 | 225.20 | 211.62 | 140.59 |
| MEST | 72.82 | 82.87 | 192.11 | 159.78 | 125.97 | 179.05 | 309.12 | 325.50 | 175.09 | 113.31 |
| COL6A3 | 0.55 | 0.32 | 29.98 | 39.41 | 21.46 | 4.18 | 306.29 | 300.77 | 48.21 | 12.32 |
| NID2 | 2.47 | 5.72 | 91.71 | 90.27 | 16.44 | 24.63 | 301.12 | 300.12 | 38.71 | 11.78 |
| SST | 0.44 | 1.23 | 13.35 | 4.66 | 0.95 | 7.15 | 251.15 | 154.94 | 2.91 | 14.63 |
| VCAN | 86.14 | 38.27 | 43.21 | 52.84 | 16.44 | 23.72 | 240.88 | 191.28 | 16.23 | 8.20 |
| SLC9A3R1 | 32.36 | 54.89 | 126.68 | 104.30 | 130.68 | 317.08 | 240.64 | 251.57 | 108.59 | 184.11 |
| COL4A2 | 47.99 | 43.39 | 89.40 | 91.77 | 63.56 | 34.83 | 233.53 | 210.37 | 81.28 | 49.92 |
| PCOLCE | 9.47 | 12.79 | 23.65 | 19.85 | 26.88 | 58.09 | 214.43 | 178.07 | 27.30 | 65.09 |
| B2M | 60.46 | 71.60 | 108.61 | 109.99 | 84.25 | 91.12 | 211.82 | 175.18 | 66.20 | 76.60 |
| COL4A1 | 50.20 | 34.77 | 84.25 | 85.53 | 54.92 | 21.20 | 195.82 | 172.19 | 80.15 | 24.26 |
| FKBP10 | 76.99 | 100.98 | 67.03 | 69.10 | 59.88 | 79.44 | 192.70 | 172.28 | 66.11 | 75.90 |
| COL5A2 | 14.50 | 2.04 | 28.45 | 49.28 | 46.49 | 26.47 | 192.23 | 143.17 | 63.28 | 20.41 |
| FRZB | 3.78 | 14.50 | 6.49 | 2.28 | 0.85 | 9.66 | 188.56 | 112.58 | 2.10 | 2.90 |
| BST2 | 31.43 | 104.47 | 33.94 | 13.38 | 11.78 | 50.65 | 166.17 | 172.75 | 6.75 | 16.94 |

| | | | | | | | | | | |
|---|---|---|---|---|---|---|---|---|---|---|
| MFAP4 | 4.08 | 2.82 | 28.18 | 44.93 | 3.16 | 4.75 | 154.54 | 139.82 | 4.22 | 3.58 |
| PLTP | 44.17 | 53.30 | 44.67 | 40.86 | 22.06 | 35.84 | 152.42 | 118.83 | 18.49 | 28.94 |
| TMEM98 | 46.54 | 59.15 | 59.87 | 71.45 | 46.52 | 104.66 | 151.14 | 142.68 | 49.45 | 63.84 |
| PITX2 | 2.68 | 1.64 | 56.15 | 64.33 | 2.45 | 15.02 | 139.37 | 115.90 | 5.53 | 5.55 |
| TFPI | 5.52 | 7.01 | 28.70 | 19.32 | 15.60 | 27.11 | 135.50 | 98.76 | 12.84 | 8.26 |
| FGFR2 | 39.28 | 38.90 | 34.37 | 42.17 | 40.73 | 35.72 | 135.22 | 109.30 | 42.58 | 19.40 |
| AQP1 | 0.05 | 0.18 | 21.70 | 16.96 | 1.39 | 15.38 | 103.58 | 73.02 | 8.56 | 9.02 |
| PEG10 | 35.44 | 31.20 | 44.94 | 34.78 | 36.59 | 23.87 | 98.92 | 82.29 | 48.07 | 15.11 |
| GNG11 | 12.84 | 6.42 | 7.35 | 4.90 | 3.13 | 6.81 | 92.53 | 59.37 | 6.18 | 9.34 |
| CRHBP | 0.00 | 0.07 | 16.28 | 8.00 | 0.34 | 1.72 | 91.15 | 63.54 | 1.66 | 1.42 |
| IL6ST | 2.33 | 1.23 | 16.29 | 25.60 | 8.04 | 7.80 | 88.58 | 69.83 | 13.36 | 3.19 |
| MRC2 | 9.66 | 13.16 | 23.23 | 31.53 | 12.37 | 15.69 | 83.85 | 79.76 | 12.62 | 12.86 |
| SERPINF1 | 14.83 | 26.55 | 6.23 | 1.83 | 3.87 | 9.61 | 81.61 | 57.60 | 3.81 | 3.78 |
| GABARAPL1 | 22.80 | 25.99 | 45.33 | 39.71 | 27.09 | 13.64 | 80.30 | 57.35 | 27.90 | 28.17 |
| FMOD | 0.01 | 0.03 | 4.25 | 6.85 | 1.05 | 1.51 | 77.35 | 48.96 | 1.63 | 1.25 |
| ADAMTS1 | 9.86 | 3.23 | 27.98 | 38.69 | 23.67 | 16.61 | 75.59 | 66.18 | 28.62 | 11.47 |
| CHN1 | 27.60 | 33.96 | 15.38 | 24.37 | 13.82 | 15.85 | 74.32 | 49.90 | 14.36 | 11.24 |
| IMPAD1 | 10.29 | 6.36 | 29.31 | 40.74 | 11.54 | 11.21 | 73.99 | 61.48 | 12.69 | 6.49 |
| CHSY1 | 31.93 | 11.48 | 26.35 | 41.75 | 13.59 | 11.71 | 71.36 | 58.51 | 11.91 | 5.60 |
| MAN1A1 | 1.69 | 1.09 | 9.72 | 16.72 | 6.64 | 5.62 | 71.07 | 51.32 | 14.32 | 3.18 |
| SLN | 0.00 | 0.14 | 9.30 | 3.31 | 1.30 | 4.80 | 68.32 | 44.01 | 3.95 | 8.46 |
| PPIC | 9.07 | 5.00 | 21.67 | 23.14 | 17.47 | 15.44 | 66.52 | 51.16 | 20.28 | 19.02 |
| MYRF | 2.43 | 3.34 | 14.46 | 13.03 | 18.71 | 12.64 | 66.49 | 59.31 | 31.74 | 17.24 |
| ATP7B | 0.97 | 1.61 | 8.31 | 13.42 | 6.08 | 4.37 | 64.88 | 54.90 | 7.79 | 2.34 |
| LEPRE1 | 22.57 | 23.20 | 23.17 | 32.63 | 18.90 | 22.60 | 63.33 | 56.73 | 18.79 | 23.44 |
| HGF | 0.00 | 0.10 | 5.37 | 10.28 | 0.18 | 0.28 | 59.30 | 36.10 | 0.85 | 0.30 |
| P4HA1 | 9.98 | 19.15 | 17.82 | 25.37 | 14.33 | 18.69 | 56.34 | 45.77 | 12.77 | 9.83 |
| ADAMTS9 | 2.32 | 2.08 | 21.48 | 23.32 | 15.87 | 4.71 | 55.91 | 47.21 | 22.50 | 6.44 |
| PAMR1 | 2.80 | 18.63 | 26.70 | 20.80 | 5.48 | 9.50 | 54.47 | 54.42 | 3.69 | 3.27 |
| SPON1 | 1.95 | 2.53 | 14.97 | 15.17 | 2.10 | 4.17 | 51.08 | 47.46 | 1.68 | 0.89 |
| TGFBR3 | 1.77 | 2.36 | 4.43 | 3.48 | 1.18 | 1.35 | 50.80 | 34.26 | 1.65 | 0.80 |
| TPST1 | 4.92 | 7.10 | 23.11 | 23.84 | 9.05 | 10.68 | 50.73 | 43.68 | 10.22 | 6.60 |
| S1PR3 | 2.76 | 2.22 | 22.58 | 23.74 | 8.18 | 4.71 | 50.66 | 52.69 | 7.84 | 3.90 |
| NID1 | 17.12 | 7.39 | 10.99 | 13.98 | 3.36 | 4.39 | 50.49 | 44.54 | 5.49 | 1.26 |
| PNRC1 | 7.44 | 21.33 | 18.56 | 16.59 | 25.37 | 34.88 | 50.42 | 43.98 | 28.61 | 28.78 |
| CHST2 | 9.23 | 4.73 | 4.86 | 4.22 | 0.71 | 2.76 | 50.41 | 40.65 | 1.02 | 1.23 |
| EGFL7 | 14.69 | 38.82 | 19.87 | 19.39 | 11.74 | 28.50 | 49.96 | 54.83 | 8.78 | 32.08 |
| REN | 0.00 | 0.03 | 1.07 | 0.09 | 0.04 | 0.26 | 49.55 | 31.02 | 0.07 | 0.17 |
| AEBP1 | 9.28 | 13.05 | 17.81 | 24.41 | 11.44 | 15.72 | 48.33 | 43.59 | 11.99 | 13.28 |
| ENPP2 | 1.22 | 4.00 | 8.91 | 4.58 | 1.39 | 4.17 | 46.28 | 28.43 | 1.58 | 0.87 |
| VEGFA | 7.27 | 8.92 | 16.32 | 19.41 | 13.84 | 6.79 | 45.96 | 55.98 | 14.46 | 10.49 |
| JUNB | 2.66 | 12.47 | 6.69 | 6.73 | 4.68 | 12.60 | 44.64 | 39.48 | 9.58 | 21.50 |

| | | | | | | | | | | |
|---|---|---|---|---|---|---|---|---|---|---|
| SOCS3 | 3.92 | 3.15 | 10.95 | 12.15 | 24.53 | 36.57 | 44.61 | 40.04 | 20.13 | 19.73 |
| SLIT2 | 1.93 | 0.85 | 7.37 | 14.85 | 6.92 | 3.94 | 44.50 | 41.75 | 11.74 | 1.70 |
| CREB3L1 | 3.03 | 4.39 | 27.22 | 13.22 | 9.74 | 9.16 | 44.21 | 37.33 | 12.67 | 15.84 |
| EGFL6 | 0.24 | 0.30 | 0.93 | 0.31 | 0.21 | 0.29 | 43.31 | 25.65 | 1.28 | 0.93 |
| RAMP2 | 10.39 | 14.44 | 10.35 | 11.43 | 3.29 | 11.36 | 42.93 | 35.03 | 2.74 | 8.22 |
| GPR124 | 0.59 | 1.19 | 17.97 | 21.56 | 6.95 | 4.03 | 41.50 | 48.33 | 9.92 | 5.25 |
| CD248 | 3.82 | 2.49 | 6.68 | 4.28 | 6.44 | 6.05 | 41.43 | 33.70 | 8.68 | 13.71 |
| SEPP1 | 5.80 | 7.58 | 13.88 | 6.81 | 16.46 | 21.35 | 41.21 | 26.28 | 11.75 | 7.41 |
| ST6GALNAC3 | 7.14 | 12.24 | 13.86 | 19.69 | 16.11 | 18.21 | 41.00 | 34.29 | 15.78 | 7.92 |
| SLC40A1 | 0.36 | 0.15 | 8.99 | 6.94 | 4.67 | 4.14 | 40.68 | 26.77 | 6.02 | 3.05 |
| BGN | 3.23 | 2.17 | 5.93 | 5.56 | 2.54 | 2.74 | 38.60 | 33.52 | 3.32 | 4.60 |
| EGFLAM | 0.38 | 0.36 | 13.27 | 12.50 | 0.93 | 2.04 | 37.15 | 27.47 | 1.01 | 1.17 |
| EMILIN1 | 2.59 | 4.69 | 4.64 | 7.38 | 5.06 | 12.08 | 37.11 | 35.39 | 5.33 | 9.55 |
| THBD | 0.38 | 0.05 | 0.62 | 1.00 | 1.15 | 2.38 | 37.01 | 27.12 | 2.40 | 1.65 |
| STRA6 | 8.69 | 10.45 | 21.27 | 10.11 | 14.17 | 27.42 | 36.92 | 36.87 | 11.91 | 15.56 |
| STMN2 | 0.79 | 2.08 | 2.97 | 0.90 | 2.91 | 2.43 | 36.21 | 19.18 | 4.79 | 6.82 |
| P4HA2 | 2.70 | 8.44 | 9.87 | 12.90 | 8.11 | 7.54 | 35.17 | 30.39 | 9.04 | 9.79 |
| TCF21 | 0.07 | 0.00 | 7.90 | 9.88 | 2.44 | 6.67 | 34.66 | 28.09 | 2.24 | 3.38 |
| GPX7 | 8.75 | 13.86 | 16.98 | 19.73 | 11.11 | 21.75 | 33.77 | 30.56 | 11.31 | 17.62 |
| TXNIP | 5.43 | 23.01 | 12.33 | 16.99 | 7.50 | 15.51 | 33.66 | 43.29 | 6.28 | 3.93 |
| PCMTD2 | 12.40 | 14.96 | 15.28 | 19.29 | 17.62 | 15.48 | 33.58 | 29.63 | 17.09 | 7.30 |
| C12orf23 | 4.54 | 4.64 | 7.83 | 11.98 | 10.44 | 10.17 | 33.11 | 22.71 | 13.90 | 7.54 |
| FREM1 | 0.25 | 0.48 | 7.53 | 9.96 | 6.14 | 4.46 | 32.69 | 31.70 | 5.66 | 3.11 |
| COL15A1 | 0.02 | 0.06 | 0.39 | 0.18 | 0.10 | 0.09 | 32.31 | 21.62 | 0.53 | 0.15 |
| COL21A1 | 0.62 | 0.47 | 0.86 | 0.68 | 1.27 | 0.38 | 32.11 | 19.41 | 1.79 | 0.73 |
| EFNB3 | 4.56 | 12.51 | 12.59 | 9.97 | 10.03 | 17.58 | 30.26 | 33.92 | 10.08 | 8.65 |
| TMEM200A | 4.16 | 4.43 | 12.93 | 14.87 | 11.31 | 13.23 | 29.83 | 22.81 | 9.20 | 3.09 |
| MAGEH1 | 6.91 | 10.06 | 10.15 | 11.30 | 12.43 | 17.90 | 29.76 | 23.78 | 13.95 | 16.59 |
| FAM110B | 0.77 | 1.65 | 5.30 | 3.80 | 6.68 | 6.67 | 29.56 | 22.16 | 4.68 | 5.96 |
| TEK | 8.17 | 8.63 | 13.41 | 9.38 | 3.79 | 3.83 | 29.43 | 24.04 | 3.69 | 1.18 |
| NR4A1 | 5.41 | 8.87 | 12.56 | 10.97 | 8.31 | 10.56 | 28.55 | 22.87 | 11.13 | 17.08 |
| ADAM12 | 0.07 | 0.38 | 2.28 | 3.84 | 0.37 | 0.27 | 28.35 | 22.29 | 1.14 | 0.25 |
| ADAMTS15 | 0.46 | 0.34 | 9.28 | 1.83 | 1.51 | 2.16 | 28.29 | 24.17 | 1.93 | 1.35 |
| ZEB2 | 4.10 | 10.37 | 13.49 | 15.43 | 6.00 | 5.40 | 28.00 | 21.79 | 7.95 | 1.88 |
| NDRG2 | 5.89 | 11.12 | 11.10 | 13.09 | 6.85 | 20.57 | 27.80 | 23.61 | 6.60 | 8.86 |
| NPNT | 0.10 | 0.04 | 1.74 | 1.37 | 2.63 | 0.97 | 27.80 | 16.44 | 5.67 | 2.55 |
| DIO3 | 1.10 | 1.36 | 16.57 | 11.89 | 16.36 | 32.95 | 27.64 | 21.22 | 8.92 | 14.41 |
| KCNN2 | 9.87 | 9.00 | 5.20 | 5.37 | 7.35 | 12.18 | 27.21 | 20.32 | 4.79 | 4.69 |
| COBLL1 | 0.92 | 0.56 | 8.37 | 8.62 | 4.18 | 3.00 | 26.23 | 17.39 | 6.08 | 1.38 |
| PDCD4 | 3.99 | 7.00 | 13.16 | 8.35 | 10.02 | 13.43 | 26.03 | 20.71 | 10.66 | 6.81 |
| TBX20 | 0.07 | 0.00 | 3.40 | 13.04 | 2.07 | 0.50 | 25.58 | 25.24 | 1.72 | 0.10 |
| ZNF503 | 0.29 | 6.05 | 10.04 | 19.27 | 7.39 | 41.92 | 25.19 | 36.26 | 9.16 | 11.18 |
| RPP25 | 10.96 | 19.03 | 8.67 | 8.56 | 3.71 | 7.32 | 25.14 | 24.19 | 3.51 | 6.64 |

| | | | | | | | | | | |
|---|---|---|---|---|---|---|---|---|---|---|
| TSPAN5 | 5.86 | 11.24 | 17.06 | 15.19 | 10.36 | 8.16 | 25.08 | 23.33 | 9.37 | 6.60 |
| ECM1 | 1.77 | 0.73 | 3.19 | 2.63 | 1.26 | 1.00 | 24.41 | 18.36 | 1.57 | 2.65 |
| IFI16 | 1.30 | 1.88 | 8.48 | 7.18 | 8.14 | 6.25 | 23.97 | 19.40 | 5.64 | 3.32 |
| TSHZ1 | 0.27 | 0.51 | 5.94 | 5.90 | 2.44 | 4.97 | 23.76 | 22.13 | 2.28 | 0.66 |
| VTN | 0.60 | 0.87 | 6.32 | 4.44 | 2.80 | 6.88 | 23.72 | 16.26 | 2.98 | 5.54 |
| CLIP3 | 4.02 | 20.63 | 11.05 | 6.33 | 5.68 | 9.22 | 23.45 | 21.67 | 5.73 | 3.67 |
| SLC22A17 | 8.47 | 25.05 | 10.94 | 6.66 | 8.12 | 19.35 | 22.94 | 25.76 | 5.91 | 10.69 |
| STC1 | 8.34 | 6.78 | 2.99 | 0.81 | 2.48 | 1.75 | 22.80 | 13.97 | 0.91 | 0.38 |
| DSC3 | 0.35 | 0.63 | 2.96 | 3.86 | 0.57 | 1.88 | 22.74 | 15.79 | 1.78 | 0.55 |
| ISLR | 1.12 | 0.34 | 4.09 | 2.78 | 0.86 | 7.24 | 22.46 | 17.97 | 1.23 | 2.31 |
| COL14A1 | 1.62 | 2.35 | 1.33 | 0.74 | 0.80 | 1.01 | 22.38 | 14.63 | 0.62 | 0.19 |
| DRD2 | 0.48 | 2.14 | 4.67 | 4.15 | 0.15 | 1.16 | 21.71 | 19.26 | 0.50 | 0.81 |
| ENTPD3 | 0.04 | 0.12 | 2.22 | 2.38 | 0.09 | 0.48 | 21.14 | 13.76 | 0.38 | 0.27 |
| NTRK3 | 1.51 | 2.43 | 2.45 | 2.84 | 0.68 | 1.08 | 21.04 | 15.88 | 1.22 | 0.64 |
| ANXA8L2 | 0.05 | 0.00 | 0.96 | 0.44 | 0.95 | 1.36 | 21.02 | 11.92 | 2.61 | 2.78 |
| CADM3 | 0.89 | 2.47 | 2.67 | 1.85 | 0.75 | 1.16 | 20.04 | 15.00 | 1.19 | 0.91 |
| CHPF2 | 6.63 | 5.50 | 9.73 | 8.63 | 10.10 | 8.94 | 19.94 | 18.93 | 10.79 | 9.65 |
| RAB37 | 1.03 | 2.34 | 3.59 | 2.99 | 3.38 | 4.68 | 19.52 | 16.92 | 2.28 | 3.17 |
| PARM1 | 1.56 | 1.20 | 0.68 | 0.31 | 0.34 | 1.25 | 19.39 | 11.46 | 0.29 | 0.24 |
| HOXC8 | 0.04 | 0.04 | 6.00 | 4.83 | 0.52 | 7.61 | 18.69 | 17.80 | 1.71 | 1.35 |
| STON1 | 1.84 | 5.07 | 2.87 | 5.40 | 5.90 | 3.93 | 18.67 | 14.89 | 4.14 | 1.24 |
| MGP | 0.00 | 0.00 | 0.12 | 0.06 | 0.04 | 0.14 | 18.41 | 9.52 | 0.07 | 0.23 |
| PLVAP | 0.07 | 0.09 | 0.89 | 0.08 | 0.14 | 0.60 | 18.41 | 16.02 | 0.10 | 0.21 |
| RHPN2 | 9.81 | 13.79 | 6.31 | 3.36 | 6.64 | 5.66 | 18.29 | 13.68 | 5.72 | 4.00 |
| TNS1 | 1.17 | 0.55 | 5.57 | 7.63 | 2.09 | 1.07 | 18.06 | 18.53 | 3.62 | 1.18 |
| ABO | 0.06 | 0.09 | 3.60 | 1.01 | 0.14 | 0.00 | 17.92 | 12.89 | 0.25 | 0.08 |
| CD34 | 0.25 | 0.23 | 0.52 | 0.06 | 0.06 | 0.96 | 17.73 | 15.11 | 0.16 | 0.36 |
| VEGFC | 0.31 | 0.67 | 3.04 | 6.79 | 0.48 | 1.84 | 17.54 | 17.07 | 0.93 | 0.61 |
| SPON2 | 0.35 | 2.81 | 2.52 | 1.28 | 0.73 | 1.36 | 17.46 | 13.83 | 0.56 | 1.96 |
| LRFN5 | 2.22 | 0.41 | 4.25 | 9.83 | 1.55 | 1.47 | 17.09 | 15.66 | 2.46 | 1.23 |
| PTGIS | 6.42 | 9.96 | 5.67 | 3.13 | 11.24 | 10.79 | 16.55 | 15.55 | 7.95 | 6.07 |
| PKIA | 4.42 | 6.18 | 3.98 | 3.05 | 3.85 | 3.81 | 16.44 | 10.32 | 3.37 | 1.44 |
| OSBPL10 | 5.90 | 3.06 | 5.33 | 4.53 | 2.74 | 2.09 | 16.40 | 13.08 | 3.19 | 1.94 |
| DOK5 | 1.94 | 5.47 | 5.90 | 5.40 | 3.22 | 3.14 | 16.22 | 11.53 | 3.38 | 3.29 |
| GRK5 | 0.84 | 2.20 | 5.99 | 5.06 | 5.00 | 3.96 | 16.00 | 13.93 | 6.95 | 6.36 |
| RAB38 | 5.39 | 6.02 | 2.13 | 1.17 | 3.86 | 8.62 | 15.86 | 10.70 | 3.89 | 4.68 |
| TBX2 | 0.01 | 0.00 | 1.38 | 2.36 | 0.76 | 6.35 | 15.70 | 12.85 | 1.06 | 1.73 |
| ZFPM2 | 0.03 | 0.15 | 0.99 | 2.11 | 0.14 | 1.20 | 15.18 | 10.93 | 0.95 | 0.28 |
| S1PR1 | 0.41 | 1.01 | 5.70 | 5.45 | 0.17 | 1.63 | 14.95 | 14.20 | 0.82 | 0.40 |
| TBC1D4 | 6.13 | 5.30 | 2.24 | 4.68 | 1.87 | 2.41 | 14.82 | 12.92 | 1.74 | 0.50 |
| HTRA3 | 1.87 | 3.47 | 4.00 | 4.80 | 1.89 | 3.83 | 14.41 | 13.23 | 2.89 | 5.42 |
| MORC4 | 0.98 | 0.63 | 6.33 | 2.57 | 3.88 | 1.96 | 14.23 | 8.68 | 5.91 | 3.85 |
| ZADH2 | 1.06 | 1.74 | 5.33 | 4.76 | 3.19 | 4.30 | 13.99 | 13.68 | 2.68 | 1.67 |

| | | | | | | | | | | |
|---|---|---|---|---|---|---|---|---|---|---|
| FLRT2 | 1.64 | 1.57 | 7.22 | 5.66 | 3.22 | 1.39 | 13.76 | 11.02 | 4.19 | 1.03 |
| KISS1 | 0.00 | 0.00 | 0.35 | 0.00 | 0.36 | 0.32 | 13.75 | 7.74 | 0.24 | 0.73 |
| CLEC1B | 0.00 | 0.00 | 3.66 | 2.61 | 0.12 | 0.64 | 13.71 | 9.47 | 1.17 | 1.45 |
| CDH5 | 0.46 | 0.13 | 0.42 | 0.11 | 1.12 | 0.92 | 13.55 | 11.32 | 0.92 | 0.62 |
| PGM5 | 0.01 | 0.01 | 0.91 | 1.24 | 1.73 | 2.12 | 13.41 | 12.64 | 2.36 | 1.28 |
| RGL1 | 2.21 | 1.57 | 6.43 | 4.35 | 1.90 | 1.91 | 13.32 | 10.42 | 2.74 | 1.06 |
| RHOBTB1 | 4.29 | 3.76 | 7.26 | 6.06 | 5.66 | 3.98 | 13.28 | 10.94 | 6.13 | 2.92 |
| NFATC4 | 3.30 | 14.02 | 3.01 | 3.80 | 7.75 | 6.60 | 13.28 | 12.87 | 7.56 | 10.46 |
| IL11RA | 1.68 | 4.28 | 4.13 | 3.27 | 3.64 | 4.71 | 13.15 | 11.11 | 3.63 | 5.59 |
| SRPX | 3.00 | 4.94 | 2.63 | 1.67 | 2.10 | 2.73 | 12.86 | 10.21 | 1.66 | 1.58 |
| F10 | 0.78 | 3.42 | 2.20 | 2.82 | 1.26 | 3.15 | 12.66 | 10.38 | 0.85 | 1.58 |
| MGAT3 | 1.45 | 4.27 | 5.16 | 6.85 | 5.65 | 4.78 | 12.64 | 12.82 | 5.55 | 4.88 |
| ESAM | 1.18 | 0.40 | 2.12 | 0.28 | 0.25 | 0.58 | 12.42 | 10.12 | 0.46 | 0.65 |
| PLAGL1 | 1.09 | 3.19 | 8.65 | 5.20 | 5.41 | 5.41 | 12.38 | 14.44 | 4.36 | 1.70 |
| GATA5 | 0.11 | 0.30 | 1.09 | 3.42 | 4.21 | 17.95 | 12.38 | 11.09 | 3.29 | 5.08 |
| SOX6 | 0.03 | 0.02 | 1.09 | 1.60 | 0.84 | 1.20 | 12.08 | 10.78 | 1.82 | 0.71 |
| FCGRT | 2.22 | 6.72 | 3.28 | 2.21 | 4.23 | 6.39 | 11.96 | 9.38 | 4.25 | 10.09 |
| P2RY1 | 5.76 | 1.23 | 2.50 | 3.68 | 0.68 | 0.32 | 11.83 | 9.58 | 0.87 | 0.11 |
| EPOR | 1.95 | 6.28 | 2.34 | 5.11 | 3.00 | 3.59 | 11.73 | 11.62 | 2.67 | 3.32 |
| BMP5 | 0.14 | 0.72 | 4.24 | 2.37 | 1.94 | 3.18 | 11.40 | 12.67 | 1.51 | 0.50 |
| JAZF1 | 3.85 | 1.90 | 1.96 | 3.71 | 1.01 | 0.99 | 11.40 | 7.25 | 1.08 | 0.62 |
| IGDCC4 | 4.28 | 6.49 | 3.77 | 4.63 | 1.70 | 1.36 | 11.09 | 10.23 | 2.14 | 0.56 |
| CYGB | 0.71 | 0.80 | 2.30 | 4.59 | 0.12 | 0.58 | 10.95 | 9.22 | 0.28 | 0.50 |
| GREM2 | 0.03 | 0.01 | 0.57 | 1.21 | 0.88 | 0.45 | 10.90 | 6.55 | 0.69 | 0.53 |
| UAP1L1 | 1.74 | 2.13 | 4.40 | 4.92 | 2.94 | 0.94 | 10.89 | 9.61 | 2.96 | 2.70 |
| TSHZ3 | 0.78 | 0.52 | 3.00 | 4.59 | 2.10 | 2.18 | 10.80 | 10.38 | 3.40 | 1.24 |
| FMO4 | 0.82 | 1.66 | 1.33 | 1.38 | 1.63 | 0.47 | 10.75 | 7.02 | 1.34 | 0.81 |
| PROS1 | 1.24 | 1.41 | 3.41 | 2.62 | 4.57 | 5.58 | 10.75 | 7.28 | 4.80 | 1.88 |
| CALB2 | 0.88 | 4.92 | 1.94 | 1.56 | 3.91 | 3.71 | 10.66 | 5.98 | 4.39 | 8.22 |
| ICAM2 | 0.29 | 0.33 | 0.31 | 0.29 | 0.10 | 0.58 | 10.59 | 8.44 | 0.00 | 0.23 |
| CLIP4 | 0.03 | 0.14 | 0.49 | 0.80 | 0.49 | 0.62 | 10.53 | 7.08 | 0.69 | 0.21 |
| PRPH | 0.33 | 1.01 | 3.76 | 5.40 | 1.99 | 18.53 | 10.51 | 8.91 | 2.91 | 5.01 |
| LAMC3 | 4.61 | 1.82 | 2.97 | 5.39 | 0.29 | 0.16 | 10.50 | 10.19 | 0.39 | 0.41 |
| PID1 | 0.68 | 0.75 | 1.98 | 2.30 | 0.67 | 0.27 | 10.38 | 6.49 | 1.04 | 0.61 |
| ORAI3 | 3.26 | 7.86 | 2.80 | 3.54 | 4.55 | 3.78 | 10.26 | 10.01 | 4.15 | 4.29 |
| ITGA8 | 0.04 | 0.06 | 1.65 | 3.25 | 1.39 | 0.39 | 9.97 | 9.68 | 1.29 | 0.25 |
| MMP1 | 0.00 | 0.00 | 1.19 | 0.96 | 0.03 | 0.48 | 9.95 | 7.03 | 0.92 | 0.67 |
| C2orf72 | 0.30 | 1.49 | 1.53 | 1.88 | 2.73 | 1.65 | 9.95 | 9.67 | 1.93 | 1.93 |
| PROK1 | 0.00 | 0.00 | 1.63 | 0.73 | 0.00 | 0.18 | 9.91 | 7.19 | 0.00 | 0.05 |
| ANGPT1 | 0.92 | 1.17 | 0.51 | 0.66 | 0.49 | 1.21 | 9.71 | 6.21 | 0.27 | 0.13 |
| C1S | 3.41 | 1.45 | 0.78 | 0.71 | 0.65 | 0.34 | 9.62 | 5.71 | 2.73 | 1.83 |
| PXK | 1.80 | 1.95 | 3.55 | 4.05 | 2.70 | 2.01 | 9.57 | 7.63 | 2.79 | 1.07 |
| TIE1 | 1.75 | 0.43 | 0.28 | 0.18 | 0.19 | 0.53 | 9.50 | 7.30 | 0.25 | 0.42 |

| | | | | | | | | | | |
|---|---|---|---|---|---|---|---|---|---|---|
| KYNU | 0.13 | 0.27 | 2.25 | 0.79 | 0.45 | 0.55 | 9.18 | 4.88 | 0.29 | 0.40 |
| CLDN5 | 0.23 | 1.12 | 0.17 | 0.23 | 0.16 | 0.97 | 9.17 | 7.18 | 0.20 | 0.54 |
| HTR7 | 3.04 | 0.40 | 1.24 | 2.43 | 1.16 | 0.39 | 9.16 | 5.49 | 0.90 | 0.37 |
| CYSLTR2 | 0.00 | 0.00 | 0.30 | 0.07 | 0.12 | 0.02 | 9.14 | 5.17 | 0.33 | 0.00 |
| CD93 | 0.03 | 0.00 | 0.07 | 0.05 | 0.01 | 0.07 | 8.97 | 6.56 | 0.07 | 0.03 |
| C5orf4 | 0.44 | 3.59 | 1.54 | 1.49 | 2.01 | 1.00 | 8.86 | 6.24 | 1.33 | 1.03 |
| SLC26A2 | 3.33 | 2.77 | 4.38 | 4.90 | 2.57 | 2.29 | 8.76 | 7.77 | 2.54 | 0.91 |
| CPED1 | 0.04 | 0.03 | 1.15 | 0.90 | 0.17 | 0.24 | 8.73 | 6.52 | 0.32 | 0.04 |
| AKR1B15 | 0.00 | 0.00 | 0.95 | 0.22 | 0.07 | 0.25 | 8.69 | 6.47 | 0.30 | 0.34 |
| COL8A2 | 0.41 | 0.58 | 0.15 | 0.13 | 0.07 | 0.25 | 8.49 | 5.08 | 0.35 | 0.36 |
| SH3RF2 | 0.49 | 0.22 | 2.58 | 2.51 | 2.08 | 2.15 | 8.18 | 8.12 | 3.31 | 2.85 |
| MMP16 | 2.70 | 1.09 | 0.86 | 2.53 | 1.00 | 0.55 | 8.14 | 6.71 | 0.81 | 0.16 |
| MMRN2 | 0.59 | 0.32 | 0.26 | 0.16 | 0.15 | 0.26 | 8.11 | 7.16 | 0.12 | 0.18 |
| CHST9 | 2.30 | 1.28 | 0.97 | 1.13 | 0.30 | 1.15 | 8.10 | 5.72 | 0.65 | 0.19 |
| PLCB2 | 2.52 | 1.36 | 1.68 | 1.89 | 3.32 | 2.64 | 8.06 | 7.01 | 3.26 | 3.44 |
| SLC2A10 | 2.69 | 3.27 | 1.88 | 2.56 | 2.62 | 1.73 | 7.79 | 7.06 | 2.50 | 0.92 |
| KLF2 | 0.27 | 0.14 | 2.00 | 1.10 | 1.04 | 2.57 | 7.65 | 8.95 | 1.23 | 3.59 |
| ADAMTS20 | 0.56 | 0.78 | 1.32 | 0.59 | 0.99 | 0.20 | 7.64 | 4.90 | 2.18 | 0.18 |
| GJA4 | 0.68 | 0.17 | 2.20 | 3.00 | 0.10 | 1.10 | 7.37 | 5.30 | 0.36 | 1.22 |
| FLI1 | 1.71 | 0.15 | 3.55 | 2.17 | 0.69 | 0.27 | 7.35 | 7.73 | 1.44 | 0.81 |
| WNT2 | 0.01 | 0.01 | 1.43 | 0.55 | 0.59 | 0.56 | 7.29 | 3.73 | 0.77 | 0.74 |
| MPPED2 | 2.79 | 0.58 | 2.51 | 4.22 | 0.67 | 0.77 | 7.24 | 6.39 | 0.75 | 0.41 |
| SLC34A2 | 0.39 | 0.39 | 0.49 | 0.22 | 2.01 | 0.48 | 7.14 | 3.51 | 0.55 | 0.32 |
| CDK20 | 0.99 | 2.37 | 1.94 | 2.22 | 1.83 | 2.65 | 6.96 | 5.46 | 3.00 | 2.30 |
| EBF4 | 0.36 | 1.58 | 1.31 | 1.22 | 0.61 | 1.49 | 6.94 | 7.09 | 0.68 | 1.30 |
| C12orf68 | 0.11 | 1.50 | 0.65 | 0.47 | 0.22 | 0.71 | 6.87 | 5.73 | 0.12 | 0.63 |
| PDE4B | 2.44 | 1.00 | 0.86 | 1.40 | 0.55 | 0.63 | 6.84 | 5.10 | 0.67 | 0.25 |
| MASP1 | 0.50 | 0.62 | 2.42 | 1.09 | 0.71 | 0.37 | 6.76 | 4.23 | 0.41 | 0.36 |
| PRR16 | 0.27 | 0.38 | 0.87 | 0.75 | 0.21 | 0.14 | 6.75 | 3.53 | 0.24 | 0.24 |
| CNTN5 | 0.00 | 0.01 | 0.09 | 0.09 | 0.08 | 0.17 | 6.75 | 4.22 | 0.22 | 0.07 |
| DEPTOR | 0.49 | 0.70 | 1.44 | 2.42 | 0.27 | 0.49 | 6.74 | 5.15 | 0.23 | 0.07 |
| SHANK3 | 1.84 | 5.05 | 2.68 | 3.11 | 3.84 | 3.35 | 6.68 | 8.60 | 4.01 | 2.35 |
| LMO2 | 0.30 | 0.90 | 1.11 | 0.92 | 0.40 | 0.58 | 6.41 | 6.59 | 0.59 | 0.58 |
| AKAP4 | 0.00 | 0.01 | 0.79 | 1.59 | 0.12 | 0.34 | 6.37 | 5.68 | 0.37 | 0.25 |
| LOC100506190 | 0.42 | 0.76 | 0.92 | 1.64 | 1.69 | 1.69 | 6.36 | 5.15 | 2.50 | 2.35 |
| CNTNAP1 | 2.25 | 5.94 | 1.55 | 3.04 | 1.35 | 1.25 | 6.35 | 5.98 | 1.44 | 0.71 |
| LMO3 | 0.35 | 0.29 | 0.51 | 0.62 | 0.09 | 1.02 | 6.35 | 5.80 | 0.19 | 0.08 |
| SH2D3C | 0.36 | 1.91 | 1.49 | 0.30 | 0.52 | 0.67 | 6.31 | 4.91 | 0.69 | 1.41 |
| FAM65C | 0.20 | 0.60 | 1.66 | 2.06 | 0.36 | 0.49 | 6.26 | 7.29 | 0.68 | 0.44 |
| IL2RB | 1.27 | 0.67 | 2.08 | 2.44 | 1.37 | 0.35 | 6.23 | 4.90 | 2.23 | 1.22 |
| EDN3 | 0.19 | 0.44 | 0.38 | 0.27 | 0.33 | 0.98 | 6.22 | 5.71 | 0.88 | 0.65 |
| LOX | 0.22 | 0.21 | 2.14 | 1.42 | 1.10 | 0.65 | 6.19 | 4.63 | 1.71 | 0.72 |

| | | | | | | | | | | |
|---|---|---|---|---|---|---|---|---|---|---|
| FIBIN | 0.00 | 0.03 | 0.43 | 0.82 | 0.12 | 0.31 | 6.17 | 4.22 | 0.15 | 0.12 |
| NT5E | 1.64 | 0.31 | 0.28 | 0.18 | 0.08 | 0.06 | 6.03 | 3.83 | 0.13 | 0.07 |
| ZNF467 | 0.28 | 1.59 | 0.44 | 0.30 | 1.79 | 2.86 | 5.94 | 4.76 | 1.58 | 2.51 |
| C1R | 2.72 | 2.70 | 1.16 | 0.43 | 0.68 | 1.18 | 5.92 | 3.27 | 0.70 | 0.92 |
| CSPG4 | 1.53 | 1.15 | 1.56 | 1.62 | 0.66 | 0.50 | 5.91 | 5.40 | 0.87 | 0.51 |
| ADAMTS10 | 1.45 | 1.39 | 1.26 | 1.52 | 1.42 | 1.00 | 5.71 | 5.52 | 1.37 | 1.47 |
| PYGM | 1.12 | 3.31 | 1.53 | 0.25 | 0.71 | 1.83 | 5.69 | 4.26 | 1.60 | 2.12 |
| ALOX15 | 0.20 | 0.55 | 0.56 | 0.24 | 0.64 | 0.55 | 5.67 | 4.71 | 0.84 | 0.59 |
| SRPX2 | 0.14 | 0.02 | 0.61 | 0.41 | 0.21 | 0.05 | 5.61 | 3.58 | 0.71 | 0.63 |
| CHSY3 | 0.20 | 0.10 | 0.52 | 1.11 | 0.69 | 0.39 | 5.55 | 3.47 | 1.00 | 0.30 |
| RERG | 0.11 | 0.06 | 1.20 | 0.64 | 1.58 | 2.29 | 5.50 | 2.87 | 1.87 | 0.92 |
| GLT8D2 | 2.56 | 2.43 | 0.79 | 0.86 | 1.77 | 1.58 | 5.50 | 4.27 | 1.77 | 1.36 |
| RNF112 | 0.30 | 0.81 | 0.54 | 0.55 | 0.19 | 0.29 | 5.46 | 4.61 | 0.21 | 0.23 |
| PAG1 | 0.48 | 0.11 | 2.49 | 1.91 | 0.25 | 0.26 | 5.41 | 4.32 | 0.55 | 0.24 |
| BCL6B | 0.41 | 1.59 | 0.68 | 1.63 | 0.20 | 0.34 | 5.39 | 5.42 | 0.12 | 0.10 |
| LAPTM5 | 0.36 | 0.12 | 0.79 | 0.84 | 0.18 | 0.24 | 5.39 | 4.98 | 0.31 | 0.37 |
| SLC47A1 | 2.25 | 1.91 | 1.26 | 1.19 | 0.28 | 0.40 | 5.38 | 4.08 | 0.31 | 0.23 |
| GHR | 0.21 | 0.29 | 1.27 | 1.54 | 1.41 | 0.93 | 5.37 | 4.02 | 1.72 | 0.69 |
| SEMA3E | 0.63 | 1.13 | 1.36 | 1.06 | 0.77 | 1.00 | 5.31 | 3.09 | 0.40 | 0.85 |
| PLCE1 | 0.16 | 0.22 | 1.83 | 2.19 | 1.84 | 0.47 | 5.19 | 4.46 | 1.41 | 0.57 |
| LOXL4 | 0.42 | 0.29 | 2.03 | 0.89 | 1.49 | 1.58 | 5.08 | 3.39 | 1.25 | 1.07 |
| RPS6KA5 | 0.81 | 0.98 | 0.37 | 0.84 | 1.06 | 0.93 | 5.06 | 3.13 | 0.90 | 0.09 |
| FLT4 | 2.76 | 4.86 | 1.02 | 1.29 | 0.95 | 2.42 | 5.06 | 6.09 | 0.89 | 0.97 |
| HSPA12B | 1.11 | 1.56 | 0.79 | 1.92 | 0.42 | 0.75 | 4.96 | 4.77 | 0.52 | 0.52 |
| CALCRL | 0.13 | 0.18 | 0.24 | 0.15 | 0.15 | 0.16 | 4.95 | 3.29 | 0.27 | 0.08 |
| COL22A1 | 1.33 | 0.98 | 0.70 | 0.58 | 2.14 | 2.50 | 4.85 | 4.87 | 1.96 | 1.05 |
| OIT3 | 0.00 | 0.00 | 0.20 | 0.18 | 0.00 | 0.10 | 4.78 | 2.98 | 0.08 | 0.05 |
| PLXNA4 | 0.47 | 0.21 | 0.49 | 0.47 | 0.42 | 0.17 | 4.77 | 3.40 | 0.49 | 0.10 |
| RASIP1 | 0.44 | 1.35 | 0.83 | 0.68 | 0.55 | 0.94 | 4.74 | 6.37 | 0.48 | 0.86 |
| ABCA2 | 1.62 | 4.39 | 1.76 | 1.12 | 0.90 | 1.50 | 4.68 | 5.58 | 0.96 | 0.54 |
| ABCA8 | 0.00 | 0.00 | 0.14 | 0.20 | 0.01 | 0.09 | 4.66 | 2.42 | 0.02 | 0.00 |
| PABPC4L | 0.53 | 0.31 | 0.90 | 1.11 | 0.72 | 1.56 | 4.62 | 3.95 | 1.09 | 0.23 |
| GFRA2 | 0.72 | 0.98 | 2.64 | 2.66 | 1.36 | 1.50 | 4.56 | 4.38 | 1.42 | 2.15 |
| HPSE2 | 0.17 | 0.35 | 0.24 | 0.22 | 0.13 | 0.08 | 4.54 | 3.47 | 0.09 | 0.07 |
| OTUD1 | 0.60 | 1.33 | 2.38 | 1.81 | 2.19 | 2.71 | 4.53 | 4.56 | 2.45 | 2.09 |
| IFIT1 | 0.53 | 1.31 | 1.13 | 0.42 | 0.46 | 0.71 | 4.44 | 3.12 | 0.89 | 0.57 |
| TRANK1 | 0.45 | 0.94 | 0.49 | 0.76 | 0.78 | 0.72 | 4.42 | 4.14 | 0.77 | 0.26 |
| GBP4 | 0.06 | 0.05 | 0.89 | 0.36 | 0.06 | 0.23 | 4.38 | 3.78 | 0.13 | 0.24 |
| TRABD2B | 0.98 | 1.23 | 1.53 | 1.33 | 0.29 | 1.15 | 4.38 | 3.56 | 0.59 | 0.61 |
| DES | 0.53 | 0.79 | 1.67 | 1.71 | 0.46 | 0.53 | 4.36 | 3.54 | 0.42 | 0.56 |
| DARC | 0.13 | 0.35 | 0.86 | 0.47 | 0.13 | 0.36 | 4.24 | 2.73 | 0.28 | 0.52 |
| SCARF1 | 0.96 | 0.49 | 0.15 | 0.18 | 0.28 | 0.22 | 4.18 | 3.54 | 0.27 | 0.32 |
| ALOX5 | 0.10 | 0.19 | 1.19 | 1.77 | 0.17 | 0.37 | 4.15 | 3.43 | 0.25 | 0.36 |

| | | | | | | | | | | |
|---|---|---|---|---|---|---|---|---|---|---|
| CPA1 | 0.00 | 0.00 | 2.15 | 1.64 | 0.11 | 0.53 | 3.89 | 3.14 | 0.41 | 0.70 |
| LSAMP | 1.29 | 2.47 | 1.42 | 0.74 | 0.95 | 1.47 | 3.85 | 3.22 | 0.98 | 0.41 |
| CARD16 | 0.00 | 0.00 | 0.44 | 0.00 | 0.00 | 0.10 | 3.84 | 1.28 | 0.00 | 0.11 |
| SLC19A3 | 0.50 | 0.42 | 0.41 | 0.53 | 1.88 | 2.35 | 3.84 | 2.22 | 1.30 | 1.11 |
| ERG | 0.04 | 0.09 | 0.48 | 0.15 | 0.42 | 0.13 | 3.71 | 3.30 | 0.14 | 0.07 |
| LRRC4C | 0.13 | 0.27 | 0.66 | 0.83 | 0.66 | 0.47 | 3.67 | 1.68 | 0.73 | 0.38 |
| GRIN2D | 0.56 | 0.45 | 1.05 | 2.15 | 1.04 | 0.75 | 3.66 | 3.37 | 0.69 | 0.89 |
| CLEC12A | 0.00 | 0.00 | 0.54 | 0.44 | 0.00 | 0.04 | 3.63 | 2.58 | 0.03 | 0.18 |
| PLD1 | 0.37 | 0.15 | 0.91 | 0.67 | 0.50 | 0.33 | 3.58 | 2.31 | 1.04 | 0.31 |
| ART4 | 0.00 | 0.00 | 2.22 | 0.50 | 1.08 | 0.28 | 3.56 | 2.02 | 1.12 | 0.55 |
| EPHA6 | 0.15 | 0.08 | 0.15 | 0.18 | 0.11 | 0.12 | 3.52 | 1.48 | 0.17 | 0.06 |
| ASXL3 | 0.08 | 0.29 | 0.68 | 1.20 | 0.61 | 0.45 | 3.51 | 2.33 | 0.85 | 0.27 |
| ZFHX4 | 0.48 | 0.89 | 1.52 | 0.29 | 0.71 | 0.83 | 3.51 | 2.41 | 1.15 | 0.42 |
| SCUBE1 | 0.56 | 0.82 | 1.45 | 0.54 | 0.77 | 1.68 | 3.48 | 3.07 | 1.33 | 0.93 |
| CLMN | 0.51 | 0.88 | 0.62 | 0.73 | 0.98 | 0.54 | 3.46 | 3.48 | 1.21 | 0.70 |
| DKK2 | 0.53 | 0.18 | 0.29 | 0.34 | 0.21 | 0.09 | 3.46 | 2.53 | 0.40 | 0.21 |
| ZNF469 | 0.19 | 0.11 | 1.24 | 0.33 | 0.15 | 0.08 | 3.39 | 3.02 | 0.31 | 0.10 |
| CD109 | 1.39 | 0.56 | 0.91 | 1.86 | 0.37 | 0.15 | 3.29 | 2.70 | 0.37 | 0.08 |
| FAM179A | 0.09 | 0.24 | 0.51 | 0.29 | 0.07 | 0.24 | 3.29 | 2.42 | 0.13 | 0.11 |
| C9orf172 | 0.36 | 0.62 | 2.31 | 0.84 | 0.61 | 1.09 | 3.27 | 4.28 | 1.20 | 0.74 |
| PLB1 | 0.01 | 0.02 | 0.33 | 0.14 | 0.13 | 0.07 | 3.25 | 2.41 | 0.10 | 0.04 |
| IL6R | 0.76 | 0.30 | 0.72 | 0.90 | 0.37 | 0.12 | 3.16 | 2.42 | 0.59 | 0.47 |
| PDZRN4 | 0.00 | 0.04 | 0.09 | 0.08 | 0.44 | 0.53 | 3.13 | 1.26 | 0.14 | 0.07 |
| PRHOXNB | 0.00 | 0.00 | 0.78 | 1.98 | 0.00 | 2.86 | 3.13 | 4.35 | 0.12 | 0.49 |
| PCDH12 | 0.03 | 0.02 | 0.11 | 0.05 | 0.06 | 0.04 | 3.11 | 2.24 | 0.10 | 0.07 |
| SOBP | 0.95 | 2.00 | 1.01 | 0.78 | 0.60 | 1.18 | 3.06 | 2.70 | 0.50 | 0.41 |
| OSBP2 | 0.84 | 0.82 | 0.26 | 0.35 | 0.23 | 0.17 | 2.89 | 1.61 | 0.24 | 0.16 |
| PCDH20 | 0.17 | 0.23 | 0.48 | 0.44 | 0.56 | 0.81 | 2.77 | 1.21 | 0.35 | 0.08 |
| SUCNR1 | 0.00 | 0.00 | 0.00 | 0.03 | 0.00 | 0.04 | 2.72 | 0.90 | 0.00 | 0.00 |
| CAMK2A | 0.01 | 0.09 | 0.53 | 0.18 | 0.32 | 0.34 | 2.71 | 2.59 | 0.88 | 0.63 |
| TNFRSF1B | 0.43 | 0.76 | 0.33 | 0.38 | 0.19 | 0.20 | 2.71 | 2.41 | 0.27 | 0.27 |
| FAM78A | 0.48 | 1.59 | 0.43 | 0.45 | 0.29 | 0.48 | 2.69 | 2.00 | 0.23 | 0.32 |
| ATP8B4 | 0.03 | 0.49 | 0.51 | 0.96 | 0.62 | 0.25 | 2.67 | 1.87 | 1.01 | 0.42 |
| TAL1 | 0.43 | 0.99 | 0.82 | 0.28 | 0.01 | 0.19 | 2.67 | 3.94 | 0.03 | 0.03 |
| ERBB4 | 0.09 | 0.02 | 0.07 | 0.22 | 0.22 | 0.39 | 2.67 | 1.56 | 0.49 | 0.11 |
| ARHGEF15 | 0.29 | 0.37 | 0.23 | 0.26 | 0.08 | 0.16 | 2.60 | 1.94 | 0.04 | 0.06 |
| NDST3 | 0.03 | 0.08 | 0.39 | 0.76 | 0.31 | 0.17 | 2.47 | 1.50 | 0.47 | 0.14 |
| HOXA11-AS | 0.00 | 0.00 | 0.54 | 1.29 | 0.00 | 0.20 | 2.46 | 2.24 | 0.09 | 0.32 |
| PTPRH | 0.19 | 0.30 | 0.10 | 0.32 | 0.08 | 0.24 | 2.44 | 1.26 | 0.14 | 0.15 |
| MLC1 | 0.01 | 0.03 | 0.11 | 0.06 | 0.11 | 0.02 | 2.36 | 1.73 | 0.05 | 0.19 |
| SLITRK3 | 0.31 | 0.94 | 0.17 | 0.09 | 0.01 | 0.09 | 2.33 | 0.81 | 0.02 | 0.01 |
| SYNE1 | 0.13 | 0.15 | 0.76 | 0.81 | 0.36 | 0.12 | 2.27 | 1.95 | 0.72 | 0.14 |
| MS4A4A | 0.00 | 0.00 | 0.00 | 0.00 | 0.07 | 0.07 | 2.26 | 0.96 | 0.00 | 0.08 |

| | | | | | | | | | | |
|---|---|---|---|---|---|---|---|---|---|---|
| SHE | 0.05 | 0.18 | 0.14 | 0.28 | 0.06 | 0.08 | 2.17 | 1.98 | 0.06 | 0.04 |
| MITF | 0.17 | 0.06 | 0.63 | 0.81 | 0.49 | 0.63 | 2.13 | 1.39 | 0.64 | 0.29 |
| GLYAT | 0.00 | 0.00 | 0.48 | 0.21 | 0.13 | 0.17 | 2.12 | 1.72 | 0.18 | 0.12 |
| DNASE1L3 | 0.00 | 0.00 | 0.29 | 0.13 | 0.00 | 0.07 | 2.01 | 0.87 | 0.11 | 0.00 |
| PTGFR | 0.02 | 0.08 | 0.11 | 0.18 | 0.03 | 0.00 | 1.99 | 0.99 | 0.11 | 0.04 |
| MAST4 | 0.49 | 0.37 | 1.27 | 1.12 | 0.91 | 0.37 | 1.92 | 1.50 | 1.00 | 0.96 |
| DCC | 0.22 | 0.16 | 0.46 | 0.26 | 0.30 | 0.15 | 1.90 | 1.30 | 0.31 | 0.08 |
| CACNA1A | 0.79 | 1.89 | 0.46 | 0.18 | 0.76 | 0.98 | 1.87 | 1.54 | 0.70 | 0.39 |
| AADAC | 0.00 | 0.00 | 0.08 | 0.00 | 0.00 | 0.00 | 1.77 | 0.43 | 0.00 | 0.00 |
| ADAMTSL1 | 0.59 | 0.74 | 0.06 | 0.05 | 0.03 | 0.07 | 1.76 | 0.77 | 0.08 | 0.03 |
| SLCO2B1 | 0.00 | 0.00 | 0.17 | 0.13 | 0.14 | 0.15 | 1.67 | 0.97 | 0.15 | 0.21 |
| OSR2 | 0.00 | 0.00 | 0.47 | 0.12 | 0.15 | 0.20 | 1.64 | 0.74 | 0.37 | 0.27 |
| RALYL | 0.00 | 0.00 | 0.39 | 0.63 | 0.37 | 0.67 | 1.61 | 0.84 | 0.48 | 0.34 |
| MECOM | 0.09 | 0.18 | 0.34 | 0.21 | 0.67 | 0.88 | 1.58 | 0.81 | 0.48 | 0.20 |
| SLC8A3 | 0.02 | 0.21 | 0.15 | 0.34 | 0.15 | 0.09 | 1.57 | 1.69 | 0.06 | 0.12 |
| CLEC12B | 0.00 | 0.00 | 0.18 | 0.16 | 0.00 | 0.00 | 1.57 | 0.50 | 0.00 | 0.05 |
| BCO2 | 0.05 | 0.03 | 0.50 | 0.42 | 0.20 | 0.41 | 1.55 | 0.97 | 0.35 | 0.17 |
| NLRP1 | 0.06 | 0.02 | 0.12 | 0.07 | 0.23 | 0.06 | 1.54 | 0.48 | 0.37 | 0.20 |
| CCRL2 | 0.00 | 0.00 | 0.05 | 0.03 | 0.00 | 0.00 | 1.46 | 0.52 | 0.00 | 0.00 |
| PLEK2 | 0.00 | 0.00 | 0.20 | 0.03 | 0.07 | 0.43 | 1.43 | 1.12 | 0.23 | 0.21 |
| TECRL | 0.00 | 0.00 | 0.56 | 0.49 | 0.58 | 0.93 | 1.41 | 2.22 | 0.57 | 0.42 |
| TBX5 | 0.03 | 0.00 | 0.00 | 0.00 | 0.29 | 1.27 | 1.41 | 0.72 | 0.50 | 0.22 |
| ADAMTSL3 | 0.14 | 0.19 | 0.26 | 0.25 | 0.24 | 0.13 | 1.36 | 0.70 | 0.40 | 0.05 |
| GRIA1 | 0.23 | 0.38 | 0.15 | 0.05 | 0.15 | 0.24 | 1.35 | 0.64 | 0.09 | 0.04 |
| HOXD3 | 0.00 | 0.00 | 0.26 | 0.22 | 0.09 | 2.34 | 1.34 | 0.80 | 0.04 | 0.05 |
| KRT24 | 0.00 | 0.00 | 0.00 | 0.00 | 0.00 | 0.00 | 1.31 | 0.27 | 0.00 | 0.00 |
| IFIT2 | 0.00 | 0.00 | 0.19 | 0.36 | 0.00 | 0.03 | 1.28 | 0.40 | 0.05 | 0.03 |
| CETP | 0.00 | 0.00 | 0.00 | 0.00 | 0.00 | 0.00 | 1.23 | 0.38 | 0.00 | 0.04 |
| PTPRB | 0.21 | 0.03 | 0.19 | 0.10 | 0.06 | 0.02 | 1.17 | 0.61 | 0.07 | 0.01 |
| RNASE13 | 0.00 | 0.00 | 0.24 | 0.19 | 0.00 | 0.00 | 1.10 | 0.35 | 0.09 | 0.04 |
| LYST | 0.02 | 0.04 | 0.18 | 0.35 | 0.24 | 0.08 | 1.09 | 0.45 | 0.37 | 0.08 |
| MYBPH | 0.00 | 0.00 | 0.27 | 0.27 | 0.06 | 0.37 | 1.04 | 0.79 | 0.05 | 0.07 |
| GDF2 | 0.00 | 0.00 | 0.17 | 0.09 | 0.00 | 0.00 | 1.02 | 0.46 | 0.00 | 0.00 |
| PRL | 0.00 | 0.00 | 0.22 | 0.19 | 0.00 | 0.06 | 0.98 | 0.21 | 0.00 | 0.00 |
| VAV1 | 0.16 | 0.12 | 0.00 | 0.00 | 0.00 | 0.02 | 0.93 | 0.70 | 0.02 | 0.02 |
| LOC100505495 | 0.00 | 0.00 | 0.02 | 0.00 | 0.00 | 0.02 | 0.86 | 0.41 | 0.01 | 0.02 |
| ABCG1 | 0.07 | 0.07 | 0.20 | 0.21 | 0.07 | 0.14 | 0.86 | 0.32 | 0.09 | 0.02 |
| CYP2E1 | 0.00 | 0.00 | 0.23 | 0.08 | 0.13 | 0.29 | 0.85 | 0.21 | 0.20 | 0.15 |
| TSHZ2 | 0.03 | 0.13 | 0.09 | 0.04 | 0.46 | 0.74 | 0.83 | 0.32 | 0.40 | 0.31 |
| CIDEC | 0.00 | 0.00 | 0.32 | 0.08 | 0.05 | 0.20 | 0.79 | 0.23 | 0.04 | 0.11 |
| CSF2RB | 0.00 | 0.00 | 0.03 | 0.04 | 0.13 | 0.02 | 0.78 | 0.33 | 0.14 | 0.08 |
| GIMAP6 | 0.00 | 0.00 | 0.07 | 0.16 | 0.01 | 0.00 | 0.75 | 0.29 | 0.00 | 0.00 |

| | | | | | | | | | | |
|---|---|---|---|---|---|---|---|---|---|---|
| EVX2 | 0.00 | 0.00 | 0.16 | 0.22 | 0.00 | 0.15 | 0.70 | 0.35 | 0.03 | 0.04 |
| BANK1 | 0.04 | 0.00 | 0.00 | 0.00 | 0.00 | 0.02 | 0.69 | 0.28 | 0.06 | 0.02 |
| TYROBP | 0.00 | 0.00 | 0.00 | 0.00 | 0.00 | 0.00 | 0.68 | 0.71 | 0.11 | 0.14 |
| GRIA2 | 0.00 | 0.00 | 0.01 | 0.00 | 0.00 | 0.00 | 0.67 | 0.21 | 0.04 | 0.00 |
| EMCN | 0.00 | 0.00 | 0.01 | 0.00 | 0.04 | 0.10 | 0.65 | 0.34 | 0.06 | 0.03 |
| CD69 | 0.10 | 0.00 | 0.00 | 0.00 | 0.03 | 0.00 | 0.64 | 0.49 | 0.00 | 0.00 |
| CSMD1 | 0.00 | 0.01 | 0.05 | 0.05 | 0.02 | 0.01 | 0.63 | 0.33 | 0.05 | 0.01 |
| HOXD-AS2 | 0.00 | 0.00 | 0.15 | 0.15 | 0.00 | 1.32 | 0.60 | 0.99 | 0.17 | 0.43 |
| IL1RN | 0.00 | 0.00 | 0.12 | 0.00 | 0.00 | 0.00 | 0.59 | 0.25 | 0.00 | 0.07 |

**Table S4. Primers for Genomic DNA PCR to confirm 2A-mTomato knock-in.**

| Aim | Forward primer | Reverse primer | $T_m$ |
|---|---|---|---|
| Test positive integration | gctccagactgccttgggaaa | gaagaagaaattgccaactgaca | 60 |
| Test random integration | cgagcggctggacctggcta | gtctccttccgtgtttcagttag | 60 |
| Confirmation of PGK-puro removal | gggcgacgtgctggctctgt | catgaactctttgatgacctcct | 60 |
| Confirmation of homozygote | cccgccgtggattcaaagggt | tccatagagggtaaaggacgctt | 60 |

**Table S5. Primers for WNT5A promoter cloning and E-box A motif mutation.**

| Primer name | Purpose | Forward primer | Reverse primer | $T_m$ |
|---|---|---|---|---|
| WNT5A-promoter | WNT5A promoter cloning | ccgagctcttacgcgtgcaggggctatg ggcgatgca | ccaagcttacttagatcgcagcaactcct gggcttaatattc | 60 |
| WNT5A-ΔE-box A | WNT5A E-box A deletion | ccgagctcttacgcgtgcaggggctatg ggcgatgca | ccaagcttacttagatcgcacgtgagttttt tgatggcaagat | 60 |
| Overlap-1 | WNT5A E-box A point mutation | ccgagctcttacgcgtgcaggggctatg ggcgatgca | tctggagagggaagaaaggagtggatg tttattgcctctc | 60 |
| Overlap-2 | WNT5A E-box A point mutation | gagaggcaataaacatccactcctttcttc cctctccaga | ccaagcttacttagatcgcagcaactcct gggcttaatattc | 60 |

**Supplementary Videos**

**Movie S1. MESP1-mTomato cells emerging and migrating together.**

MESP1-mTomato$^+$ cells first appeared in a scattered manner, then migrated actively, displayed stronger red fluorescence, and converged together to form bigger cell clumps.

**Movie S2. Beating cardiac tissues differentiated from MESP1-mTomato reporter cells.**

MESP1-mTomato reporter cells were induced with 12 μmol/L CHIR for 24 h. 48 h after CHIR removal, 5 μmol/L IWP2 was added for 48 h. After IWP2 removal, cells were cultured in RPMI-1640 / B-27 minus insulin medium for another 2 days before switching to RPMI-1640 / B-27 medium until day 12. Time-lapse movie of beating cardiomyocytes was recorded on day 12.

**Movie S3. Calcium transient in beating foci from MESP1-mTomato reporter cells.**

After 12 days of differentiation, MESP1-mTomato reporter cells derived cardiomyocytes were incubated with 10 μmol/L Fluo-4 AM. Calcium transients were recorded by time-lapse imaging.

**Movie S4. Calcium transient after Isoproterenol treatment.**

Isoproterenol (5 μmol/L) was added to MESP1-mTomato reporter cells derived cardiomyocytes filmed above, Calcium transients were recorded by time-lapse imaging.
